# Cell and tissue reprogramming: Unlocking a new era in medical drug discovery

**DOI:** 10.1016/j.pharmr.2025.100077

**Published:** 2025-06-26

**Authors:** Chandan K. Sen, Andrew J. Friday, Sashwati Roy

**Affiliations:** McGowan Institute for Regenerative Medicine and Department of Surgery, University of Pittsburgh, Pittsburgh, Pennsylvania

## Abstract

Recent advancements in cell and tissue biology have fundamentally changed our understanding of cellular behavior, revealing that both stem and nonstem cells exhibit remarkable plasticity and adaptability. This discovery has paved the way for revolutionary medical drug therapies that leverage cell and tissue reprogramming to repair or regenerate damaged tissues, offering new hope for conditions that were once considered irreversible. Tissue reprogramming involves the activation of specific molecular pathways to convert the function of residual tissue to compensate for the loss of tissue function to aging, trauma, or disease processes. By targeting these pathways, emerging drugs can promote regenerative processes, enabling the restoration of tissue function lost due to aging, injury, or disease. These therapies have shown promising results in preclinical studies addressing a wide range of diseases. Unlike traditional treatments, which focus primarily on managing symptoms, tissue reprogramming therapies offer a dynamic approach that can fundamentally alter cellular states, leading to functional recovery. This review explores the current state of cell and tissue reprogramming, highlighting its potential applications in regenerative medicine and the challenges that must be addressed for successful clinical translation. As our understanding of cellular plasticity continues to evolve, these innovative therapies stand at the forefront of a new era in medicine, with the potential to transform treatment paradigms and significantly improve patient outcomes across a wide range of conditions.

## I. Introduction

The concept of terminal differentiation—where cells reach their final, specialized state—has long shaped our understanding of tissue function. While this idea is relatively modern in scientific literature, the notion that organs serve distinct roles dates back to antiquity. Aristotle, in De Partibus Animalium (350 BC),^1^ pondered the specialized functions of organs, although his insights were later eclipsed by medieval mysticism. The Renaissance reignited anatomical science through the pioneering dissections of Andreas Vesalius and Leonardo da Vinci, who revealed the heart’s central role in circulation, challenging centuries of dogma.^2,3^ These foundational discoveries laid the groundwork for modern biology’s view of tissues as specialized yet remarkably adaptable systems.

Today, we are entering a new era where the plasticity of differentiated cells is no longer a biological curiosity but a therapeutic opportunity. Tissue reprogramming—achieved through small molecules or gene-based delivery of reprogramming factors—is emerging as a powerful strategy at the intersection of pharmacology and regenerative medicine.^4^ Although gene therapy uses genetic material rather than traditional drugs, it aligns with pharmacological principles and is regulated by the US Food and Drug Administration (FDA) as a form of drug therapy.^5^ Under the 21st Century Cures Act, the FDA’s Regenerative Medicine Advanced Therapy (RMAT) designation has accelerated the approval of over a dozen reprogramming-based therapies, with more than 100 candidates in the pipeline.^6–10^

By harnessing the inherent flexibility of cell identity, researchers are developing novel interventions to repair, regenerate, and even enhance tissue function. This review explores the scientific and regulatory landscape of therapeutic tissue reprogramming, highlighting its transformative potential in drug development and personalized medicine.

### A. Tissue differentiation in developmental biology

To understand how organs and tissues become irreversibly specialized, biologists have long studied early embryonic development. A single-cell zygote undergoes 2 symmetrical divisions, followed by an asymmetrical one at the 4-cell stage. In 1817, Christian Pander, studying chick embryos, identified the 3 germ layers—ectoderm, mesoderm, and endoderm—and emphasized their interdependence in development.^11^ He was the first to chronologically describe embryogenesis, earning him the title “Father of Embryology.” Pander’s work challenged the prevailing theory of preformation, which claimed embryos were fully formed from the start, instead supporting the idea of progressive cell differentiation.^12^

Recent studies have traced tissue origins in mammalian embryos, identifying the first signs of differentiation at the 8-cell stage. At this point, cells begin to polarize: outer cells form the trophectoderm (TE), which becomes the placenta, while inner cells form the inner cell mass (ICM). The ICM further differentiates into the epiblast (EPI), which gives rise to the embryo, and the primitive endoderm (PE), which forms the yolk sac.^13, 14^ A key regulator of this process is the transcription factor Cdx2, which is unevenly expressed. Higher Cdx2 levels in outer cells suppress pluripotency genes *Oct4* and *Nanog*, promoting TE fate.^15^ In contrast, inner cells with lower Cdx2 maintain pluripotency through elevated *Oct4* and *Nanog*. Disrupting Cdx2 expression leads to loss of epithelial integrity and failed implantation.^16^ This early polarization highlights one of the first transcription factor—driven decisions in embryonic cell fate.

Within the ICM, the EPI and PE lineages are regulated by distinct transcription factors: Nanog promotes EPI fate, while Gata4/6 drive PE differentiation.^17^ Two main hypotheses explain how these early lineage decisions occur:

1. Positional/induction model—cell fate is determined by spatial positioning.^18^
2. Stochastic model—fate is initially random, with actin-driven sorting later aligning cells into correct positions.^14, 18^

E-cadherin—mediated adhesion and actin cytoskeletal flows help establish apical polarity and mechanosensitive cell contacts.^19, 20^ This polarization and TE differentiation occur between the 8- and 32-cell stages.^21^ During the 8–16-cell stage, the embryo undergoes compaction, a key morphogenetic event where loosely arranged cells form a tightly packed sphere—crucial for lineage segregation.^22^ Following compaction, TE cells contact the uterine epithelium, triggering implantation. Mural TE cells then differentiate into trophoblast giant cells, which invade maternal tissue and form vascular channels, establishing the yolk sac placenta.^20^

Postimplantation marks the onset of gastrulation, a critical phase in mammalian development when the 3 germ layers—ectoderm, mesoderm, and endoderm—are specified.^11^ While ethical constraints limit direct study of human gastrulation, animal models and in vitro systems offer valuable insights. During this stage, epithelial-to-mesenchymal transition (EMT) initiates formation of the primitive streak, which, along with migrating cells, establishes the anteroposterior axis of the EPI—essential for gastruloid formation and tissue patterning.^23^ Together with gastrulating cells, the primitive streak establishes the EPI anteroposterior asymmetric axis essential to gastruloid formation and subsequent tissue specification. In mice, the period from embryonic day (E)8 to E15.5 is crucial for endodermal differentiation into gastrointestinal tissues. With Gata4-mediated mesenchymal signaling, the endoderm forms the foregut, midgut, and hindgut (E8.5), which later develop into the stomach, small intestine, and colon, respectively (E15.5).^24^ Simultaneously, neural crest cells (NCCs) emerge from pluripotent cells along the anteroposterior axis. These multipotent cells interact with ectoderm and mesoderm to form diverse tissues, including cranial, cardiac, trunk, vagal, and sacral structures.^25^ Disruptions in NCC development can lead to neurocristopathies, such as tumors, congenital heart defects, and syndromes.^26^ For example, 3MC syndrome, caused by mutations in COLEC10/11 or MASP1/3, results in craniofacial abnormalities like cleft lip and palate.^27^ In contrast, familial medullary thyroid carcinoma arises from RET mutations in NCC-derived cells, leading to malignant thyroid cancer.^26,28^ These examples highlight how early transcriptional and positional cues shape embryonic development—and how even minor disruptions can result in significant congenital disorders. Collectively, this body of research underscores the precision and complexity of early tissue differentiation.

As development progresses, cells become increasingly specialized, with distinct identities and functions. In 1953, Paul Weiss refined the concept of “differentiation,” defining it as the emergence of visible, specialized cell structures. He described a mostly linear progression from undifferentiated to differentiated to terminally differentiated cells, noting that only the earliest cells are truly undifferentiated.^29^ For example, myoblasts are not yet terminally differentiated like mature muscle cells (myocytes), but they have already committed to a specific developmental path. Once on this path, they can only proliferate or differentiate into myocytes—an irreversible decision. A decade later, François Jacob and Jacques Monod (1963) expanded on this idea, showing that while all cells share the same DNA, they differ in protein expression, which drives their unique functions. This concept—that gene expression, not genetic content, defines cell identity—became a cornerstone of modern developmental biology and our understanding of cell specialization.^30^ The notion that cells can express a different set of proteins and have specialized functions while harboring the same genetic makeup has helped guide the understanding of specialized differentiated cell-states.

### B. Extracellular and microenvironmental influences

By 1976, biologists had recognized that most organs are not only made up of differentiated cells but also contain localized populations of precursor or “-blast” cells. These precursors reside in specialized niches—such as the crypts of Lieberkühn in the small intestine and colon—where they retain the ability to divide and replenish aging or damaged cells.^31^ This process, known as tissue homeostasis, ensures that organs are continuously renewed, with approximately 1.1% of cells replaced daily.^32^ While many tissues maintain their own local precursor cells, some—like circulating blood—rely on a centralized source. As early as 1868, Ernst Neumann discovered that blood cells are replenished by hematopoietic stem cells (HSCs) located in the bone marrow, rather than within the bloodstream itself.^32^ If the progeny of actively dividing blast cells fail to exit the cell cycle after leaving their precursor niche, they may lose the ability to differentiate and instead continue dividing uncontrollably—potentially leading to tumor formation. For example, unchecked proliferation of hepatocytes can result in hepatomas.^31^ Within a tumor microenvironment, even cells that would normally undergo terminal differentiation can be influenced by their surroundings. The immunosuppressive and hypoxic conditions of the tumor niche alter cellular metabolism and promote continued growth rather than maturation.^33,34^ Importantly, this kind of extracellular regulation is not unique to tumors. In all tissues, signals from the extracellular matrix (ECM) and surrounding environment play a critical role in controlling key cellular processes—including differentiation, proliferation, metabolism, growth, homeostasis, and quiescence.^35,36^

During embryogenesis, implantation depends on precise cell—cell and cell—matrix interactions. Key to this process are E-cadherin/*β*-catenin complexes, which regulate cell adhesion and coordinate actin cytoskeletal flow to establish spatial gradients and cell polarity.^19,20^ In parallel, focal adhesions connect embryonic cells to the ECM. These mechanosensitive complexes link the ECM to the actomyosin cytoskeleton, allowing cells to sense and respond to mechanical forces.^19^ Both cell—cell (adherens junctions) and cell—ECM (focal adhesions) are responsive to mechanical cues. These forces are converted into biochemical signals that regulate essential processes such as homeostasis, proliferation, compression, extrusion, and gene expression—notably through pathways like HIPPO signaling, which involves transcription factors YAP, TAZ, and YKI.^37,38^ Disruptions in ECM structure—often due to genetic mutations in collagen genes—can lead to a range of hereditary diseases:

- Collagen type I mutations may cause brittle bones (osteogenesis imperfecta) or excessive bone growth (hyperostosis).
- Collagen type V defects can result in fragile skin.
- Collagen type XVIII mutations are linked to vision loss or degeneration.
- Collagen type IV abnormalities can cause vascular issues and kidney dysfunction.^39^

The ECM plays a crucial role in preventing breast cancer development and progression.^40^ In the mammary gland, the basement membrane (BM) separates the epithelial cells from the surrounding stroma, which contains collagen and adipocytes.^41^ This BM not only maintains epithelial polarity and tissue architecture but also acts as a barrier to tumor formation. When this structure is disrupted, it creates a permissive environment for cancer to progress.^42^ Even when lesions form, they often remain benign as long as the BM remains intact, due to its rigidity and tumor-suppressive properties.^41^ Loss of polarity proteins, such as Scribble, can block apoptosis and disrupt polarity, leading to dysplastic or neoplastic growth. Remarkably, restoring polarity can re-establish BM-mediated tumor suppression.^40^ This illustrates how cell—ECM interactions are essential for maintaining tissue homeostasis and function.^41^

Early evidence of extracellular influence on cell differentiation came from Barker and Walker.^43^ They showed that basal cells in the vaginal epithelium respond to hormone signals in their environment. For instance, estradiol drives these cells to differentiate into squamous epithelium, while progesterone promotes differentiation into mucosal epithelium. However, once cells begin differentiating under one hormone, switching to another does not reverse their fate—demonstrating that differentiation is irreversible once initiated. This discovery laid the groundwork for understanding how hormones, growth factors, cytokines, and other extracellular signals regulate cell fate. It also inspired the idea of using these signals therapeutically—to push malignant cells toward terminal differentiation, potentially halting cancer progression.^35,44,45^

### C. Reversible terminal differentiation

The idea that terminal differentiation might be reversible laid the groundwork for today’s breakthroughs in adult cell reprogramming. A pivotal moment came in 1979, when Leslie G. Lajtha, a pioneer in hematopoiesis research, introduced a powerful framework for classifying cells based on their kinetic behavior. He proposed the following 3 categories:

1. Static cells—fully differentiated and locked into their final state.
2. Transit cells—short-lived intermediates (eg, keratinocytes and myelocytes) that lose proliferative capacity before being eliminated.
3. Stem cells—a more debated group at the time, which Lajtha^46^ argued were not strictly undifferentiated. Instead, he proposed that stem cells could exist in partially differentiated states, yet still retain the ability to self-renew and generate diverse progeny.^46^

The kinetically “static” cells were differentiated and locked into their terminal state. “Transit” cells were defined as populations (eg, keratinocytes and myelocytes) that are short-lived in the body and lose their ability to proliferate before elimination. The third classification of “stem” cells was still under debate at the time as to what defined them. Lajtha^46^ emphasized that the previous definition of stem cells as being an undifferentiated population was not entirely accurate. Rather, stem cells can exist in partially differentiated states, and these cells still exhibit the ability to self-renew and produce multiple differentiated descendants.^46^ Building on this, Gao et al^47^ later refined our understanding of differentiation by distinguishing between irreversibly differentiated and terminally differentiated cells. While both are committed to a specific lineage, irreversibly differentiated cells can still divide, whereas terminally differentiated cells are so metabolically specialized that they permanently exit the cell cycle to sustain their high functional demands.^47^

The foundation of irreversible differentiation theory traces back to Hans Spemann’s groundbreaking experiments with salamanders between 1915 and 1924. In these studies, Spemann discovered what he called the “organizer effect”—a region in the embryo capable of directing the developmental fate of surrounding cells. By transplanting presumptive neuroectoderm into areas destined to become skin, he revealed a critical window during gastrulation when cells transition from being developmentally flexible to irreversibly committed.^48^ This discovery earned him the Nobel Prize in 1935 and laid the groundwork for modern developmental biology. Decades later, in 1958, Sir John Gurdon challenged the permanence of cell fate. He hypothesized that the nucleus of an undifferentiated cell could direct full organismal development. Using *Xenopus* embryos, he demonstrated that even a nucleus from a fully differentiated intestinal epithelial cell could be reprogrammed to generate an entire organism through somatic cell nuclear transfer (SCNT).^49–51^ This was a revolutionary finding: it showed that cellular differentiation does not erase genetic potential, but rather that cell fate is influenced by context and environment, not just lineage. Gurdon’s work gained renewed attention in 1997, when Wilmut et al^52^ famously cloned Dolly the sheep—the first mammal cloned from an adult somatic cell—using the very principles Gurdon had pioneered. Together, these discoveries dismantled the idea of terminal differentiation as a 1-way path and paved the way for today’s advances in cellular reprogramming and regenerative medicine.

The cloning of Dolly the sheep by Wilmut et al^52^ was a turning point in biology. By using differentiated fetal and adult lamb cells in SCNT, Wilmut et al^52^ directly challenged the long-held belief that somatic cell differentiation was irreversible. This landmark experiment captured global attention and shifted scientific consensus toward a revolutionary idea: every cell, even in complex mammals, retains the full genetic potential to become any other cell type—if reprogrammed correctly. Building on this momentum, Hochedlinger and Jaenisch^53^ provided further proof in 2002. Using T cell receptors as lineage markers, they demonstrated that even terminally differentiated immune cells could be reprogrammed through SCNT, reinforcing the idea that cell fate is not fixed.^53^ Inspired by these breakthroughs, Shinya Yamanaka, who had grown up learning about Gurdon’s work, asked a bold question: Could cells be reprogrammed without using embryos at all? In 2006, alongside Kazutoshi Takahashi, Yamanaka screened embryonic stem cells (ESCs) and early embryos for key pluripotency factors. They identified just 4 transcription factors—Oct3/4, Sox2, c-Myc, and Klf4—which could reprogram both embryonic and adult fibroblasts into induced pluripotent stem cells (iPSCs).^54^ This discovery was nothing short of transformative. It marked the first time that fully differentiated cells were reprogrammed back to a pluripotent state without nuclear transfer, proving definitively that terminal differentiation is reversible. From Gurdon’s frogs to Dolly the sheep to iPSCs, the journey of rethinking cell identity had come full circle—opening the door to regenerative medicine, disease modeling, and personalized therapies.

## II. Plasticity and stemness

**Fig. 1.**
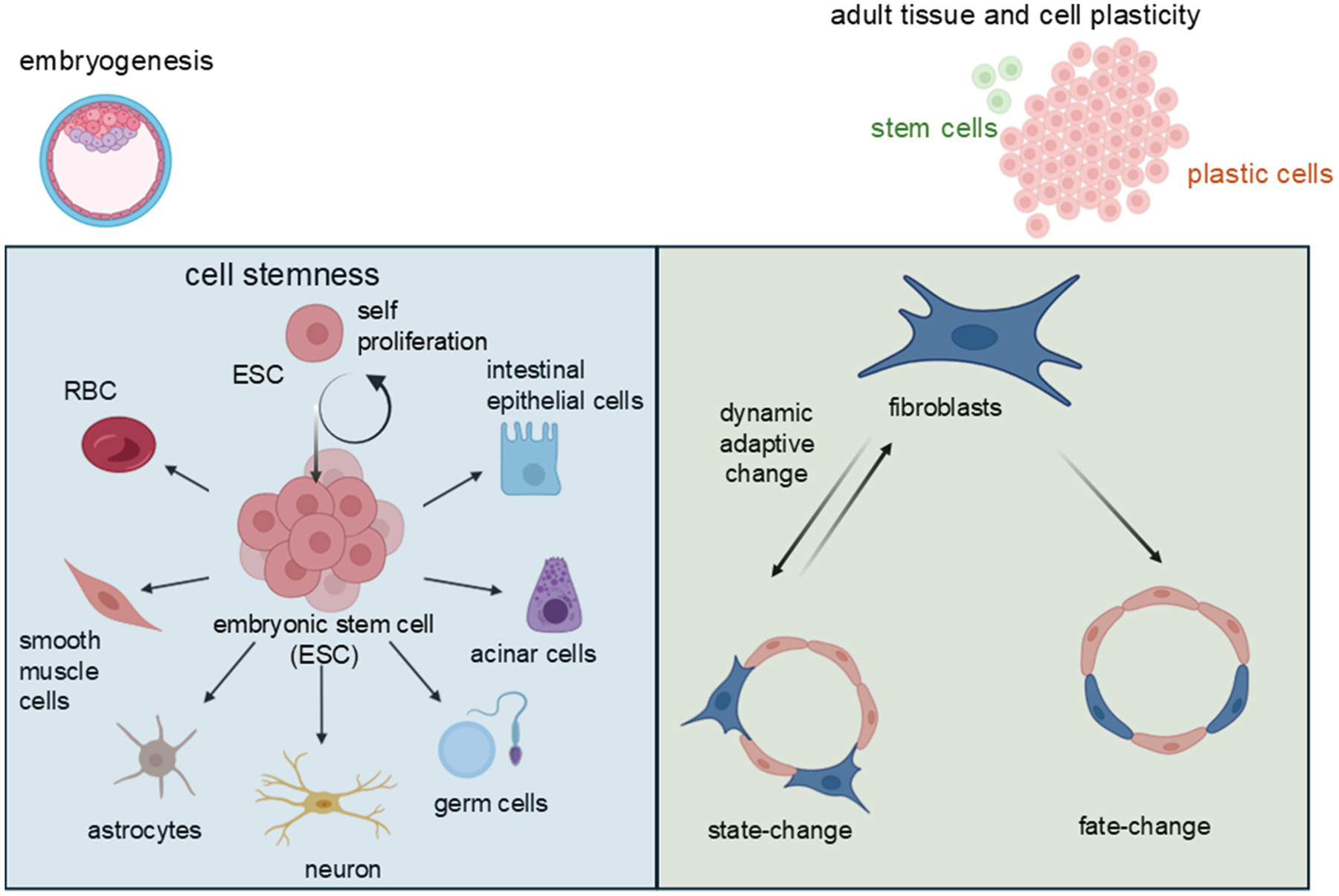
Cell and tissue plasticity during embryogenesis and adult stages of life. The embryonic stage of life features a large, highly versatile pool of stem cells that drives rapid growth and differentiation. In contrast, adult tissues contain a much smaller stem cell pool, but growing evidence suggests that many adult somatic cells retain significant plasticity. This plasticity enables 2 key processes: direct conversion (fate change), interpreted in the past as transdifferentiation, where cells fully relinquish their original identity to adopt a new one with distinct functions; and state change (not appreciated until recently with the advent of scRNASeq approaches, wherein cells partially shed their original traits while acquiring new characteristics and functions. Notably, state changes can be reversible, allowing cells to revert to their original identity under certain conditions. RBC, red blood cell.

Throughout the body, adaptation to environmental cues is vital—not just at the level of the whole organism, but down to individual cells. This adaptability is largely governed by 2 key cellular properties: plasticity and stemness. Cell plasticity refers to a cell’s ability to respond to external signals by altering its identity or behavior. This can involve reversible or permanent changes that may extend beyond what would typically be expected based on the cell’s original lineage.^55^ For example, under certain conditions, a differentiated cell might adopt new functions or even revert to a more primitive state, demonstrating a surprising degree of flexibility. In contrast, stemness is a distinct property. Rather than changing identity, stem cells maintain a stable core identity that allows them to self-renew, regulate quiescence, and generate diverse progeny. These progeny can then differentiate into specialized cell types, supporting tissue regeneration, repair, and homeostasis (Fig. 1).^56–58^ Stemness is not about becoming something else—it is about preserving the potential to become many things in response to the body’s needs. Both plasticity and stemness are most evident during the early stages of embryonic development, when cells are highly responsive and versatile. As development progresses, these capacities become increasingly restricted, and cells gradually commit to specific lineages and functions.^59,60^ Together, these 2 properties—plasticity and stemness—form the foundation of how cells adapt, regenerate, and maintain the dynamic balance of living tissues.

### A. Developmental plasticity

**Fig. 2.**
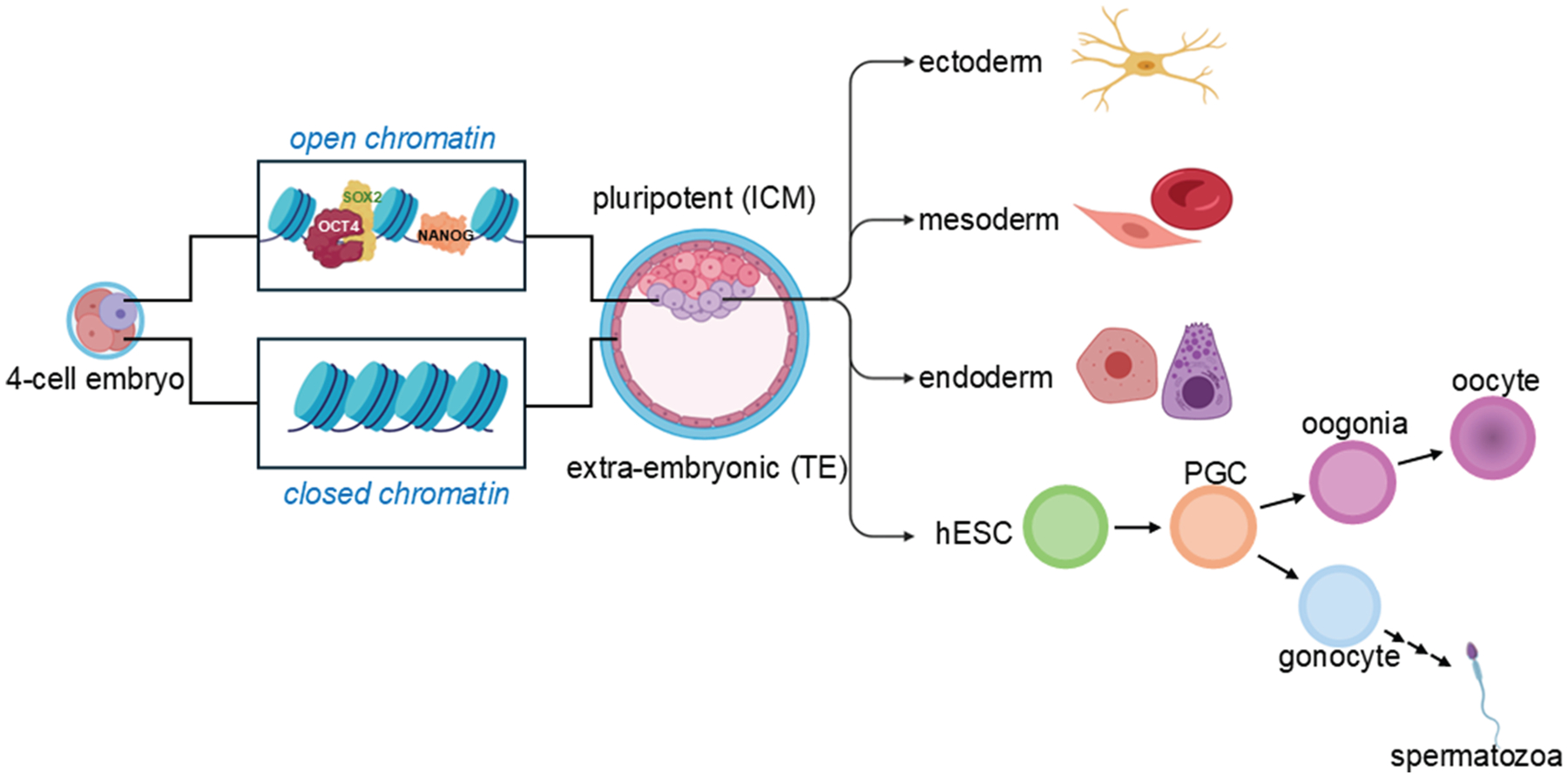
Epigenetic regulation of early embryonic plasticity. Epigenetic mechanisms regulate germ and early embryonic cell plasticity via transcriptional silencing. During the 4-cell stage, cell-fate decisions are made by heterogeneous gene expression. Closed chromatin in the TE is fundamental for lineage commitment, the repression of pluripotency genes, and the establishment of a developmental program tailored to the formation of extraembryonic tissues essential for mammalian embryogenesis. Open chromatin in the inner cell mass is crucial for maintaining a permissive and flexible epigenetic environment that supports pluripotency, developmental plasticity, and the ability to respond to differentiation signals. This dynamic accessibility is a defining feature that distinguishes the ICM from the TE and ensures proper embryonic development. During early embryogenesis, the ectoderm, mesoderm, and endoderm germ layers are established, each giving rise to specific organs and tissues. The totipotent potential of dividing embryonic cells is gradually lost as transcription factors like OCT4, SOX2, and Nanog, which regulate cell lineage potential, are segregated into different cells, thereby specializing them for specific tissue differentiation. hESCs derived from the ICM can initiate germline differentiation, particularly through BMP4-driven signaling, which leads to the formation of primordial germ cells (PGCs). During sex determination, PGCs undergo migration and mitotic/meiotic divisions, ultimately differentiating into oocytes or spermatozoa, completing the germline cycle.

During early embryonic development, cells exhibit remarkable plasticity, allowing them to adapt to changes in their environment and compensate for cell loss by altering their genetic programs and adopting new fates.^61^ For example, in the early blastocyst, if cells from a specific lineage are lost, others can step in—either through asymmetric division or transdifferentiation—to restore balance.^21^ This flexibility is especially pronounced in germ and early embryonic cells, where epigenetic regulation, particularly during periods of transcriptional silencing, plays a key role in maintaining developmental potential.^55,62^ A striking example of this plasticity is seen in the TE and ICM. While TE cells typically arise from the oocyte cortex, ICM cells can compensate for lost TE cells, demonstrating their developmental versatility (Fig. 2).^20^ After implantation, however, cell fate becomes increasingly influenced by positional cues, epigenetic modifications, and cell—cell interactions, which guide cells toward more permanent identities through asymmetric divisions.^63^ As development progresses, the embryo forms the 3 germ layers—ectoderm, mesoderm, and endoderm—each giving rise to specific tissues and organs.^11^ Yet, even at this stage, plastic cells can respond to environmental stressors such as inflammation, injury, or chronic stress by deviating from their original lineage. This can lead to metaplasia, a generally benign shift to another cell type within the same germ layer, or dysplasia, a more disordered change that may progress to neoplasia.^64^

In mouse embryos between E8.5 and E10.5, a niche of intermediate progenitor cells emerges. These cells remain multipotent and plastic within their microenvironment, and upon exiting the niche, they differentiate into organs such as the liver, pancreas, gallbladder, and bile ducts.^65,66^ However, during these critical stages, genetic mutations in precursor cells can lead to serious developmental disorders. For instance, Bamforth-Lazarus syndrome, caused by mutations in FOXE1, results in hypothyroidism, cleft palate, and epiglottic malformations. Similarly, mutations in NOTCH3 in NCCs can cause Cerebral Autosomal Dominant Arteriopathy with Subcortical Infarcts and Leukoencephalopathy, a vascular disorder marked by brain ischemia and reduced blood flow.^26^ These examples underscore the importance of precise regulation of differentiation during early development. Disruptions in these processes can have lasting consequences for organ formation and function.

At the heart of this developmental potential are ESCs, which possess both high plasticity and robust stemness. Derived from early-stage embryos such as blastocysts, ESCs can be cultured as immortal pluripotent cell lines.^67^ In their earliest stages, ESCs are totipotent, capable of giving rise to all adult cell types.^59^ As development continues, this potential becomes more restricted. Key transcription factors—OCT4, SOX2, and Nanog—are gradually segregated into specific lineages, guiding cells toward specialized fates.^21,68^ Through asymmetric divisions and progressive specialization, ESC progeny transition from pluripotent to multipotent states, gradually losing their stemness as they commit to specific tissue lineages.^59, 69,70^

### B. Adult stem cell niche and plasticity

The story of stem cell biology begins in the late 19th century, when Theodor Boveri proposed a radical idea: certain cells in the body—what we now call stem cells—have the unique ability to both self-renew and differentiate into specialized cell types.^71,72^ Fast forward to 1961, when James Till and Ernest McCulloch made a groundbreaking leap. While studying the effects of radiation on mice, they observed that certain bone marrow cells could clonally repopulate the spleen of irradiated animals.^73,74^ Their findings provided compelling evidence that bone marrow cells possess the dual capacity for self-renewal and differentiation, expanding upon Ernst Neumann’s 1868 discovery that bone marrow is the source of terminally differentiated blood cells.^74,75^ This was the first experimental proof that stem cells existed in mammals. Their work built on Ernst Neumann’s 1868 discovery that bone marrow produces blood cells, but Till and McCulloch went further—demonstrating that these cells could both self-renew and differentiate, the 2 hallmarks of stemness. In a follow-up study, they used chromosomal markers to show that individual HSCs could form clonal colonies in vivo, confirming their identity as true stem cells.^76^ This pivotal discovery marked the birth of stem cell research as a distinct scientific field.

At the heart of this field is the concept of stemness—a cellular property distinct from plasticity. While plasticity refers to a cell’s ability to change identity in response to external cues, stemness is about maintaining a stable identity while retaining the potential to proliferate, self-renew, and generate differentiated progeny that support tissue regeneration and homeostasis.^56–58^ Stem cells are unique in that they exhibit both plasticity and stemness, especially during early development.^77^ However, their importance extends far beyond embryogenesis. In adult mammals, stem cells reside in specialized niches within tissues, where they remain mostly dormant until activated by physical or chemical signals.^78^ Some of their progeny stay nearby, providing feedback signals that help regulate the niche environment.^70^ These stem cells are the body’s reservoir for repair, ready to respond to injury, stress, or cellular turnover.^79^

Adult stem cells do not operate in isolation—they reside in specialized niches that provide the support and signals necessary for their function. These niches vary depending on the regenerative demands of the tissue and can be broadly categorized into 3 types^80^:

1. High-turnover niches, such as those in the blood, skin, and intestinal epithelium, where cells are constantly renewed.
2. Basal cell replacement and repair niches, like those in skeletal muscle, which activate in response to injury.
3. Poor regenerative niches, found in tissues like the heart and nervous system, where repair is limited and slow.

**Fig. 3.**
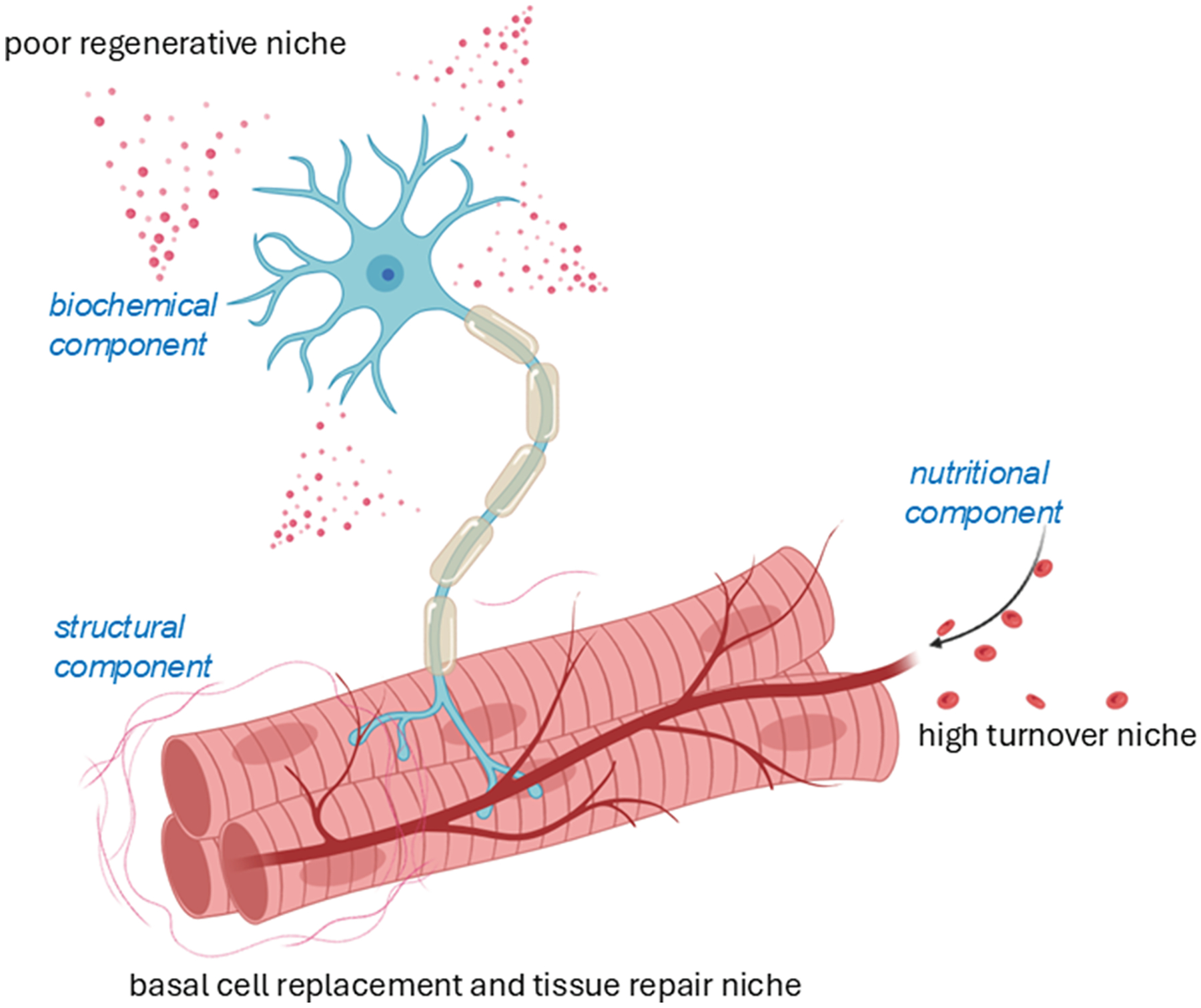
Adult stem cell niches and their regulation. Adult stem cell niches are specialized microenvironments within tissues that regulate the behavior, maintenance, and differentiation of adult stem cells. These niches provide both structural support and signaling cues to maintain tissue homeostasis and facilitate repair processes. Adult stem cell niches are categorized into 3 types based on the regenerative needs of the tissue: (1) high-turnover niches (eg, blood, skin, and intestinal epithelium), which support rapid cell renewal; (2) basal cell replacement and repair niches (eg, skeletal muscle), which enable tissue maintenance and repair; and (3) poor regenerative niches (eg, heart and nervous system), which exhibit limited regenerative capacity. The regulation of adult stem cell niches is a highly coordinated process involving intrinsic factors, local environmental cues, systemic signals, and physical interactions. This intricate network ensures stem cells function properly to maintain tissue homeostasis and respond effectively to physiological demands.

Each of these niches is finely tuned by a combination of biochemical, structural, neural, nutritional, and adhesion molecule signals that maintain tissue homeostasis and coordinate regeneration (Fig. 3).^80,81^ However, the behavior of adult stem cells within these niches is not always predictable. Research on HSCs has shown that even symmetric divisions—which are expected to produce identical daughter cells—can result in progeny that retain clonal memory or deterministic traits from their parent cells.^82^ This suggests that some adult stem cells may carry predetermined biases, potentially limiting their flexibility for therapeutic use.

The microenvironment plays a critical role in regulating these niches. For instance, hypoxia (low oxygen levels) is a common feature in many stem cell niches and helps maintain cells in an undifferentiated, quiescent state.^83^ Additionally, heterologous cell types within the niche—such as supporting stromal or immune cells—communicate with stem cells through cell—cell interactions and cell—ECM signaling. These interactions involve a complex network of hormones, growth factors, receptors, and adherens junctions that collectively guide stem cell behavior.^84^ Together, these insights reveal that stem cell function is not just a matter of intrinsic potential but also of context—a dynamic interplay between the cell and its surrounding environment that determines whether it remains dormant, divides, or differentiates.

The ECM is far more than structural scaffolding—it is a dynamic signaling hub that plays a critical role in regulating stem cell behavior within their niches. One of the key mediators of this communication is a family of receptors known as integrins, particularly *β*1 and *β*3 integrins, which link the ECM to the cytoskeleton of stem cells through pathways involving epidermal growth factor receptor signaling. β1 integrin is especially important. It promotes cell cycle activation via interleukin (IL)-3, facilitates EMT and collagen remodeling through transforming growth factor (TGF)-*β*, and regulates the endosomal trafficking of growth factor receptors.^85^ In the epidermis, high levels of integrins enhance adhesion between stem cells and the ECM, supporting the proliferation of basal stem cells.^86^ Interestingly, this adhesive relationship is counterbalanced by the transcription factor Myc, which suppresses ECM adhesion and promotes stem cell differentiation.^87^

Beyond biochemical signaling, the mechanical properties of the ECM—particularly its rigidity—also influence stem cell fate. For example, in neuroblastomas, increased ECM stiffness is associated with reduced proliferation and lower N-Myc expression, while promoting cellular differentiation.^88^ More broadly, ECM stiffness can direct lineage specification:

- Soft matrices favor neurogenic differentiation.
- Intermediate stiffness supports myogenic differentiation.
- Stiff matrices promote osteogenic differentiation.

This principle is vividly illustrated in specific tissue niches, such as the brain, muscle and bone.^89–91^ In the brain, neural progenitor stem cells are supported by a soft ECM enriched with hyaluronan, a glycosaminoglycan associated with fractones. This specialized ECM not only maintains the stem cell pool but also guides the differentiation and migration of neuronal progenitors toward the olfactory bulb, where they undergo terminal differentiation. Together, these findings highlight how the ECM—through both biochemical signals and mechanical cues—acts as a powerful regulator of stem cell fate, balancing self-renewal and differentiation to maintain tissue integrity and function.

Among the most remarkable examples of tissue regeneration in the human body is the intestinal epithelium, which undergoes complete renewal every 3 to 5 days. This rapid turnover is driven by a small but powerful population of stem cells nestled within the crypts of Lieberkühn—specialized niches located at the base of epithelial invaginations.^92^ Despite each crypt containing only 5–7 stem cells, they sustain the continuous production of all differentiated cell types needed to maintain intestinal function.^93^ These stem cells give rise to a diverse array of specialized cells, each with a distinct role:

- Enterocytes absorb nutrients.
- Goblet cells secrete protective mucus.
- Paneth cells release antimicrobial peptides.
- Tuft cells detect chemical signals and modulate immune responses.
- Microfold cells facilitate immune surveillance.
- Enteroendocrine cells secrete hormones that regulate digestion and metabolism.^94,95^

At the molecular level, this finely tuned system is regulated by transcriptional networks. One key player is SATB2, a chromatin organizer that modulates the activity of transcription factors CDX2 and HNF4A in the colon. SATB2 not only helps maintain the stem cell pool but also influences the plasticity of colonic epithelial cells—enabling them to adopt characteristics of ileal small intestine cells when needed.^96^ This intricate coordination between niche architecture, cellular diversity, and molecular regulation makes the intestinal crypt a model system for understanding how stem cells sustain tissue homeostasis and adapt to physiological demands.

The adult bone marrow is home to one of the most vital and dynamic stem cell systems in the body: HSCs, which are responsible for the continuous replenishment of blood. These stem cells reside in specialized niches that regulate their behavior through a complex interplay of cellular and molecular signals. Two primary niches support HSCs^80^:

- The osteoblastic niche, where HSCs remain in a quiescent, long-term state, and
- The vascular niche, where they are more proliferative and short-lived

The size and activity of these niches are influenced by parathyroid hormone receptor signaling on osteoblasts, as well as adherens junctions involving N-cadherin and *β*-catenin, which anchor HSCs to the bone surface. These osteoblastic cells also secrete RANKL, a growth factor that activates NF-*κ*B signaling, further shaping the niche environment.^94,97,98^ Adding another layer of complexity, bone marrow adipocytes—once thought to be passive—have emerged as highly plastic regulators of the HSC niche. Following myeloablative treatments like radiation or chemotherapy, which deplete bone marrow cells, these adipocytes can dedifferentiate and contribute to the repopulation of HSCs.^99,100^ After bone injury, they may even replace osteolineage cells, although whether this occurs through transdifferentiation or via an intermediate mesenchymal stromal cell state remains under investigation.^101^

While the bone marrow is one of the most well-characterized stem cell niches, similar microenvironments exist in nearly all adult tissues—including the skin, skeletal muscle, intestine, heart, and nervous system. Despite their diversity, these niches share several core features:

- Structural and ligand-based interactions with the ECM,
- Bidirectional signaling to regulate proliferation and differentiation,
- Immune cell—mediated control of inflammation, and
- A hypoxic microenvironment that helps preserve stem cell quiescence and potency.^102^

Thanks to their unique combination of stemness, plasticity, and their central role in tissue repair and regeneration, adult stem cell niches are now at the forefront of regenerative medicine. Several therapies based on these principles have already advanced to clinical trials, offering hope for treating a wide range of degenerative diseases and injuries.^103–109^

### C. Stemness and plasticity in nonstem adult cells

For much of the 20th century, the ability to self-renew and differentiate—known as stemness—was believed to be the exclusive domain of stem cells. However, a growing body of research has revealed a more dynamic and flexible view of cell identity, showing that even nonstem adult somatic cells can exhibit remarkable plasticity under certain conditions. This discovery has reshaped our understanding of tissue regeneration and opened new frontiers in in vivo reprogramming. One of the earliest glimpses into this hidden potential came in 1974, when Eguchi et al^110^ demonstrated that iris pigment cells in newts could transform into lens cells following lens removal—without reverting to a pluripotent state. This process, known as transdifferentiation, suggested that even mature, differentiated cells retain a latent capacity to change fate.^110^ Interest in cellular plasticity surged again in 1998, when Thomson et al^111^ successfully isolated human ESC (hESCs), reigniting questions about how cell fate is determined and whether it can be reversed. This curiosity culminated in a landmark breakthrough in 2006, when Shinya Yamanaka and Kazutoshi Takahashi reprogrammed adult skin cells into iPSCs using just 4 transcription factors. Their work proved that mature somatic cells could be reverted to a stem-like state, fundamentally challenging the belief that only stem cells possess stemness.^54^ This evolving view of cell identity has profound implications. It suggests that stemness and plasticity are not fixed traits, but rather context-dependent properties that can be activated in response to environmental cues. In fact, many nonstem adult cells can adopt stem-like behaviors during tissue injury, inflammation, or stress, contributing to regeneration and repair.^112^

A compelling example of this is found in the central nervous system (CNS). Astrocytes, a type of glial cell, have been shown to retain stem-like properties in the subventricular zone (SVZ) of the adult mouse brain. These specialized astrocytes can act as neural precursors, generating new neurons under certain conditions.^113–115^ However, the extent of their plasticity remains debated, as some studies suggest that *Neurod1* gene transfer alone is insufficient to induce full neuronal conversion.^116,117^ Following neural injury, both stem cells and astrocytes in the SVZ are activated to proliferate and differentiate, aiding in regeneration.^114,115,118^ Although the CNS is considered a poor regenerative environment (Fig. 3), it still harbors responsive cells and signaling molecules that promote neurovascular repair. For instance, in ischemic stroke models, increased expression of vascular endothelial growth factor (VEGF) and decreased angiopoietin 1 support angiogenesis, neuron survival, and repair during the subchronic phase of injury.^119,120^ VEGF expression is further enhanced by mesenchymal stem cells (MSCs), endothelial progenitor cells, and neural stem cells (NSCs), which release gaseous signaling molecules like NO and CO to promote neuroprotection and regeneration. Within days of cerebral artery occlusion, VEGF stimulates neurogenesis and angiogenesis in the dentate gyrus and SVZ, partly by upregulating brain-derived neurotrophic factor (BDNF)—all without crossing the blood—brain barrier (BBB).^120^ In the peripheral nervous system (PNS), regeneration is supported by multipotent vascular stem cells, which differentiate into perineural and perivascular cells, reinforcing the blood—nerve barrier.^121^ Even in diabetic wounds, where vascularization and nerve function are impaired, conductive hydrogels have been used to stimulate Ca^2+^ signaling and activate PI3K/AKT/MEK/ERK pathways, promoting neurovascular regeneration.^122^ Together, these discoveries reveal a powerful truth: the capacity for regeneration is not limited to classical stem cells. Many somatic cells, once thought to be terminally fixed in their roles, can be reawakened to support healing and renewal. This paradigm shift is transforming regenerative medicine, offering new hope for treating injuries and diseases by harnessing the body’s own cellular potential.

Cellular plasticity is a cornerstone of development, immune defense, and tissue regeneration. While traditionally associated with stem cells, this remarkable ability to adapt, reprogram, and shift identity is increasingly recognized in many nonstem somatic cells—especially in response to environmental cues and injury. A striking example of this adaptability is found in the innate lymphoid cells (ILCs), a family of immune cells that mirror the functions of adaptive T cells but act more rapidly and flexibly. ILCs are categorized into subtypes that resemble T helper and cytotoxic cells: ILC1s (Th1-like), ILC2s (Th2-like), ILC3s (Th17-like), natural killer (NK) cells (CD8-like), and lymphoid tissue-inducer cells, which guide lymphoid organ development.^123,124^ What makes ILCs particularly fascinating is their plasticity—the ability to transition between subtypes in response to cytokine signals. For instance, IL2 and TGF-*β* can drive an irreversible transition from NK cells to ILC1s, while other transitions (eg, ILC1 ↔ ILC2 ↔ ILC3) are reversible and regulated by cytokines like IL-12, IL-4, IL-1*β*, IL-23, and IL-18.^123–127^ In disease contexts, such as Crohn disease or chronic obstructive pulmonary disease, ILC2s can shift toward an ILC1-like phenotype, while *Candida albicans* exposure can push ILC2s toward an ILC3-like state.^125,128^

This dynamic behavior reflects a broader concept known as graded plasticity—the idea that cells exist on a spectrum of flexibility. Rather than being locked into fixed identities, many cells retain varying degrees of potential to re-enter the cell cycle, transdifferentiate, or even dedifferentiate. For example, Schwann cells in the PNS can revert to a progenitor-like state after nerve injury,^123,125,128–130^ and adipocytes from human liposuction samples have been shown to dedifferentiate into fibroblast-like cells in vitro, only to re-differentiate into fat cells under the right conditions.^131^ Similarly, bone marrow adipocytes can contribute to osteogenesis after injury, either directly or through an intermediate MSC state.^101^

Nowhere is this plasticity more evident than at the site of injury, which acts as a cradle for regeneration. When tissues are damaged, nearby cells activate their latent potential to adapt, repair, and regenerate. This concept dates back centuries—Lazzaro Spallanzani and Wilhelm Roux observed limb regeneration in salamanders, laying the foundation for our understanding of injury-induced cellular reprogramming.^132–134^ In the CNS, despite its limited regenerative capacity, injury triggers a cascade of responses. In ischemic stroke models, for instance, increased expression of VEGF and angiopoietin 1 promote angiogenesis, neurogenesis, and neuron survival.^119^ These effects are amplified by MSCs, endothelial progenitors, and NSCs, which release NO and CO to stimulate repair.^120^ Even in challenging conditions like diabetic wounds, innovative therapies such as conductive hydrogels can stimulate Ca^2+^ signaling and activate PI3K/AKT/MEK/ERK pathways, promoting neurovascular regeneration.^122^ Together, these discoveries reveal a powerful truth: cellular plasticity is not an exception—it is a fundamental feature of life. Whether in immune cells, adipocytes, glia, or fibroblasts, the ability to adapt and transform is central to how the body heals, protects, and evolves. As we continue to uncover the mechanisms behind this flexibility, we move closer to unlocking new regenerative therapies that harness the innate potential of the body’s own cells.

**Fig. 4.**
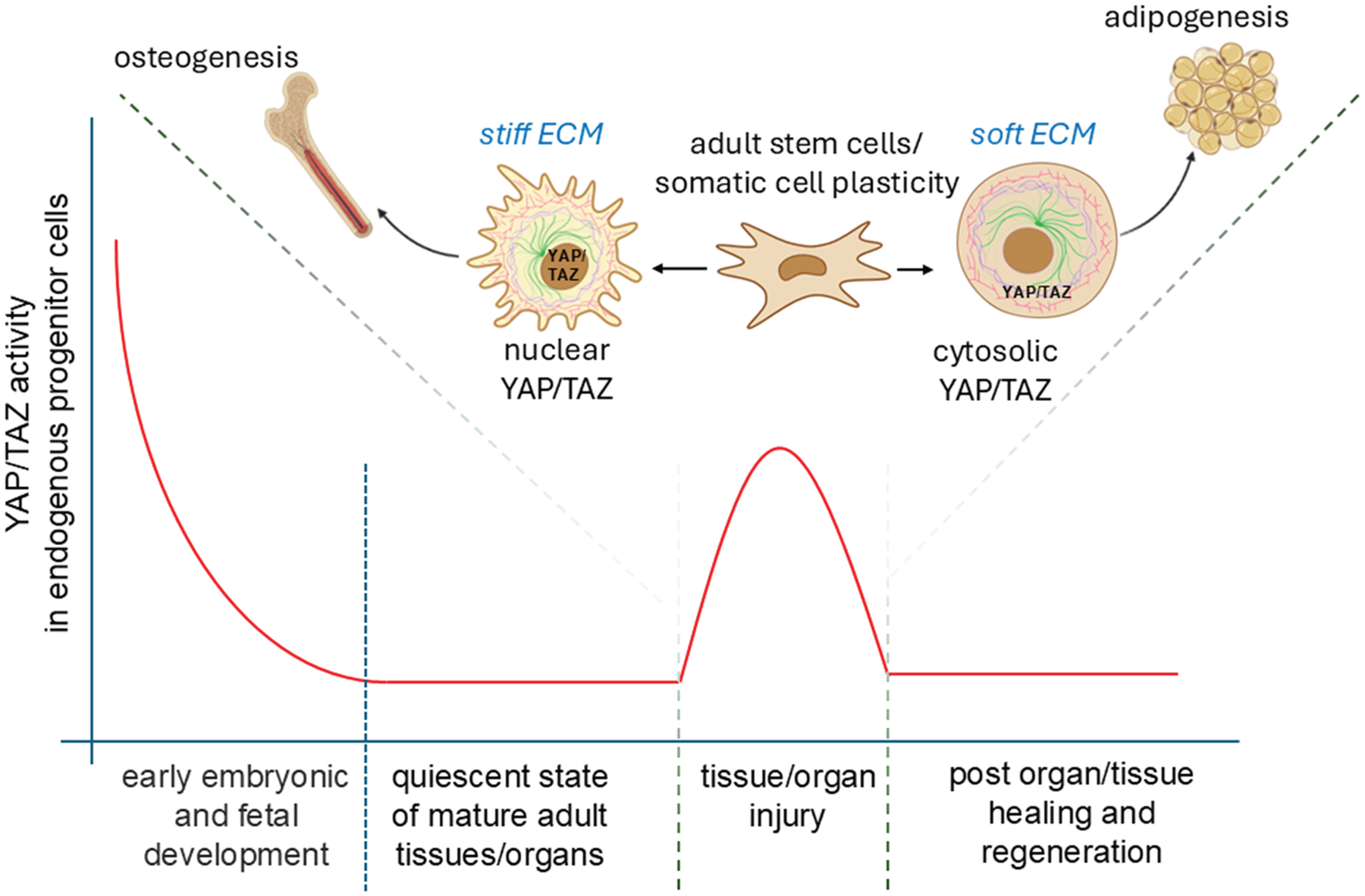
Plasticity and stemness in adult nonstem cells following injury. The YAP/TAZ pathway, key components of the Hippo signaling cascade, plays the role of master regulator in regulating the plasticity of adult somatic cells by influencing their ability to dedifferentiate, transdifferentiate, or adopt a more stem cell—like state. During embryonic and fetal stages, YAP/TAZ activity is elevated but gradually decreases after birth, ultimately reaching basal levels in quiescent adult tissues. Following injury, YAP/TAZ expression transiently increases in tandem with the mobilization and proliferation of adult stem/progenitor cells, then returns to baseline upon completion of tissue repair. MSC lineage commitment is strongly influenced by YAP/TAZ localization, which is regulated by mechanical cues. On stiff substrates, integrin clustering and focal adhesion assembly enhance F-actin polymerization and stress fiber formation, leading to increased cell spreading and nuclear translocation of YAP/TAZ, thereby promoting osteogenesis. Conversely, soft substrates reduce focal adhesion and stress fiber formation, favoring cytosolic retention of YAP/TAZ and driving adipogenesis.

**Fig. 5.**
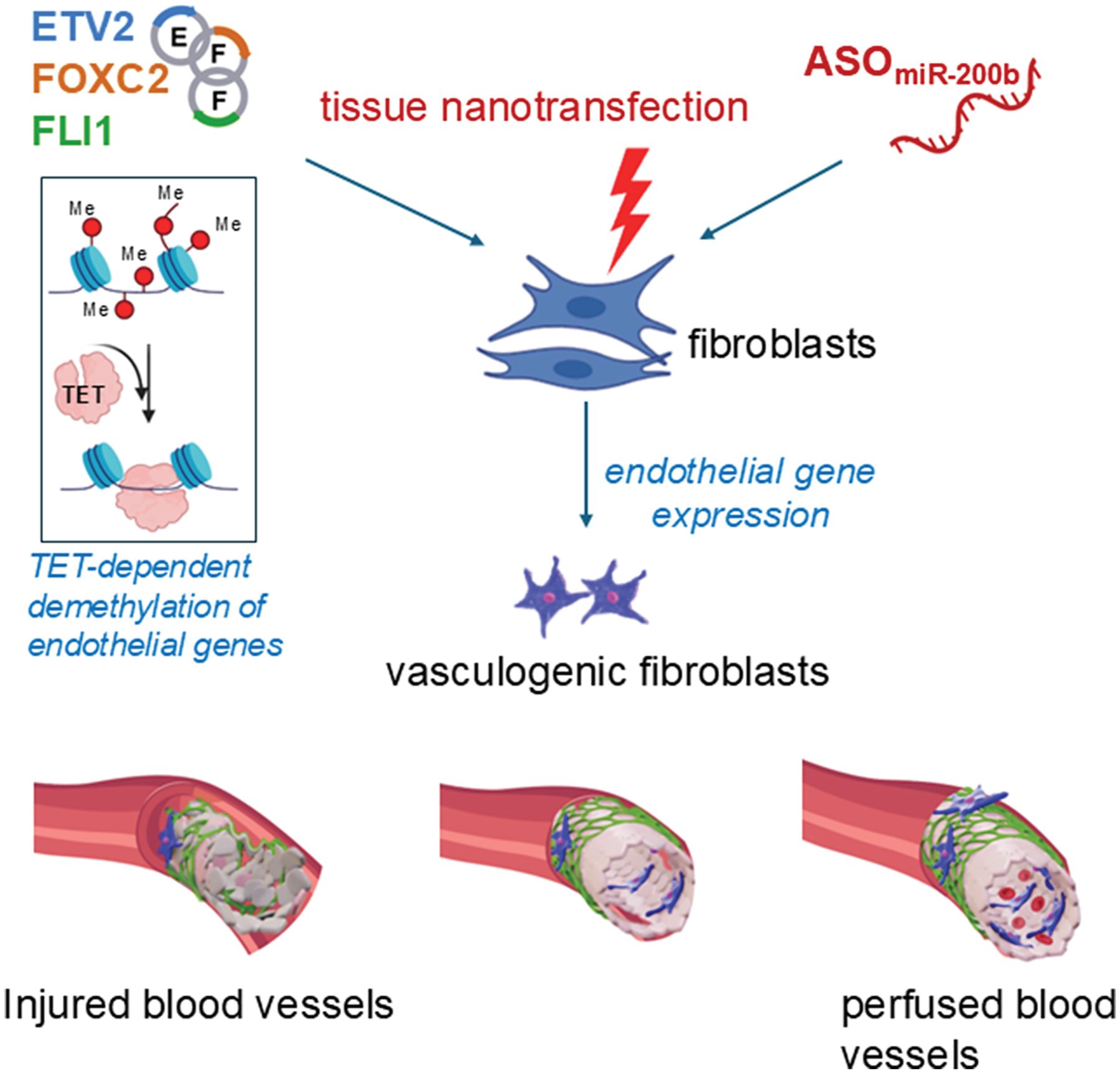
TNT rescues tissue ischemia by inducing the formation of vasculogenic fibroblasts only under ischemic conditions—an example of state change of fibroblasts. Human dermal fibroblasts when transfected with anti—miR-200b oligonucleotide demonstrated emergence of a vasculogenic subset with a distinct fibroblast transcriptome and blood vessel forming function in vivo in a FLI1-dependent manner. Vasculogenic fibroblasts can be also produced by TNT-based delivery of EFF plasmids. TET-dependent demethylation of endothelial genes in fibroblasts is involved in vasculogenic fibroblast formation.

In tissues with high turnover rates—such as the intestinal epithelium and skin—the capacity for cellular plasticity extends beyond stem cells. Even fully differentiated cells can revert to more primitive states to support wound healing and tissue regeneration. In the intestine, for example, epithelial cells not only remodel the ECM after injury but also activate the YAP/TAZ signaling pathway (YAP and TAZ). This activation drives cells into a fetal-like, regenerative state, enhancing repair (Fig. 4).^135,136^ YAP/TAZ signaling plays a similarly critical role in other tissues. In the lungs, it regulates the transition of alveolar epithelial type 2 cells into type 1 cells following injury.^137^ In the skin, YAP/TAZ influence keratinocyte self-renewal, proliferation, and differentiation, particularly through the transcription factor KLF4, which is essential for maintaining barrier function and coordinating wound healing.^138^ During skin repair, migrating keratinocytes and dermal papilla fibroblasts collaborate to rebuild the BM.^86^ Interestingly, inhibiting YAP with verteporfin has been shown to enhance fibroblast-mediated regeneration, improving mechanical strength and reducing scarring.^139^ Injury itself elicits reprogramming cues, prompting fibroblasts to adopt a vasculogenic phenotype. These reprogrammed cells rapidly contribute to new blood vessel formation, a key step in effective skin regeneration (Fig. 5).^112,140^

Beyond signaling pathways, biomaterials can also activate regenerative responses. One such material, akermanite—a calcium silicate—based bioceramic—has been shown to enhance epidermal stemness. In vitro, akermanite increases stem cell markers, boosts proliferation, and promotes migration. In mouse models, topical application on burns significantly improved reepithelialization and healing.^141^ Akermanite also enhances osteogenic potential in bone marrow stromal cells, promoting angiogenesis and bone regeneration in models of osteoporosis.^142^

Hormonal regulation further influences stemness. For instance, estrogen enhances bone marrow stromal cell stemness via the SATB2 pathway, supporting osteogenesis.^143^ Similarly, in the heart, a PDGFR inhibitor has been shown to prevent endothelial-to-mesenchymal transition after myocardial infarction (MI), improving recovery by blocking the PDGF/NF-*κ*B/HIF-1*α* pathway.^144^ Together, these findings illustrate a powerful theme: stemness and plasticity are not fixed traits confined to stem cells, but dynamic properties that can be activated in differentiated cells through injury, signaling molecules, or biomaterials. This understanding is reshaping regenerative medicine, offering new strategies to mobilize the body’s own cells for healing and repair.

## III. Tissue and organ plasticity

Tissue and organ plasticity elevate the concept of cellular plasticity to a broader biological scale, involving coordinated changes across multiple cell types, the ECM, and tissue architecture. This form of plasticity enables organs to adapt to injury, environmental shifts, and physiological demands—playing a vital role in maintaining homeostasis and ensuring survival. A classic example is the liver, renowned for its regenerative capacity since ancient times. The myth of Prometheus, whose liver regrew daily, and the landmark 1931 study by Higgins and Anderson on rat liver regeneration, both highlight this extraordinary ability.^145,146^ Another example is the skin, noted as early as the 16th century by Ambroise Paré for its self-repairing nature.^147^ These examples illustrate how tissue and organ plasticity are essential for dynamic repair and regeneration, extending the regenerative potential of individual cells to entire systems.

### A. Liver plasticity

Tissue and organ plasticity extend the concept of cellular plasticity to a broader scale, involving coordinated changes among multiple cell types, the ECM, and tissue architecture. This adaptability is essential for maintaining homeostasis and responding to injury or physiological stress. A prime example is the liver, renowned for its regenerative capacity. During murine embryogenesis, HSCs migrate to the fetal liver, where they proliferate before relocating to the spleen and eventually the bone marrow for lifelong hematopoiesis.^148^ In newborn mice, both the bone marrow and spleen are active hematopoietic sites. Remarkably, after splenectomy, the liver can resume hematopoietic activity, demonstrating its plasticity in compensating for organ loss.^149^ This regenerative ability is further highlighted by the liver’s response to partial resection, where it can regenerate up to 100% of its original mass. This process is driven not only by hepatic progenitor cells but also by mature hepatocytes, which can undergo dedifferentiation and transdifferentiation to support regrowth.^150^ This regeneration is driven not only by hepatic progenitor cells but also by fully differentiated hepatocytes that can undergo dedifferentiation and transdifferentiation.^136^

Beyond hematopoiesis, the liver also compensates for gallbladder loss. In both dogs and cats, removal of the gallbladder (cholecystectomy) prompts the hepatobiliary ducts to take over its functions—resorbing fluid and concentrating bile—highlighting the liver’s role in maintaining digestive function.^151–153^ At the cellular level, hepatic stellate cells exemplify liver plasticity. These versatile cells contribute to growth, immunity, inflammation, and metabolic regulation.^154^ After liver injury, hepatocytes may become either hyperactive and proliferative or hypertrophic with reduced metabolism, both supporting regeneration.^129,155^ However, chronic damage—such as from viral infection, alcohol, or inflammation—can activate stellate cells to transdifferentiate into myofibroblast-like cells, promoting fibrosis and impairing liver function.^154^ In chronic liver disease, hepatocyte plasticity is further revealed through transdifferentiation into cholangiocytes, a process regulated by PI3K-AKT-mTOR signaling in response to insulin. This has been observed in patients with metabolic dysfunction—associated steatotic liver disease, underscoring the liver’s adaptive capacity even under pathological conditions.^156^

### B. Skin plasticity

The story of skin plasticity begins in the Renaissance, when pioneering anatomists like Andreas Vesalius, Ambroise Paré, and Gaspare Tagliacozzi first explored the skin’s remarkable ability to heal and regenerate. Through their early experiments with skin flaps and grafts, they laid the foundation for what would eventually become modern reconstructive surgery.^157,158^ Centuries later, in the 19th and 20th centuries, scientific understanding of the skin took a transformative leap. It was discovered that the skin was not just a protective barrier—it was a dynamic, self-renewing organ. At its core were dermal and epidermal stem cell niches, with basal keratinocytes identified as the key drivers of repair and regeneration.^159^ This breakthrough redefined the biology of skin and revolutionized dermatology and plastic surgery. The culmination of this progress came in 1980, when scientists successfully performed the first autologous graft using cultured epidermal keratinocyte sheets to treat a patient with third-degree burns—a milestone that marked the beginning of regenerative skin therapy.^160^

Skin plasticity is a dynamic process driven by specialized stem cells and responsive somatic cells that work together to maintain homeostasis and repair damage. Within the epidermis and hair follicles, distinct niches house epidermal stem cells essential for skin renewal and wound healing.^161^ These stem cells can differentiate into various epidermal lineages, with their fate guided by local microenvironments in the hair follicle, interfollicular epidermis, and sebaceous glands.^162^ The regenerative potential of these cells was confirmed in studies showing that interfollicular epidermal stem cells could survive UV-induced TP53 damage and continue to self-renew.^163^ During injury, these cells migrate to the wound site and differentiate into keratinocytes, fibroblasts, and other skin cell types to restore tissue integrity.^164^ Importantly, plasticity in the skin is not limited to stem cells. Fibroblasts can enter an injury-induced state, adopting vasculogenic functions that support new blood vessel formation.^112^

Myofibroblasts, which emerge during wound healing, secrete ECM and promote tissue contraction. These cells are plastic themselves—adipocytes can convert into myofibroblasts, and under certain conditions, myofibroblasts can revert to adipocytes, especially in large wounds via BMP signaling.^164^ Key signaling pathways such as YAP/TAZ, NPGPX, and Wnt orchestrate these regenerative processes. While Wnt dysregulation can lead to fibrosis, NPGPX upregulation has been shown to improve healing in diabetic wounds.^165–167^ Modulating these pathways holds promise for reducing scarring and enhancing recovery in patients with burns and chronic skin injuries.^168,169^

Advances in skin plasticity have shown that differentiated skin cells can be reprogrammed to gain stem cell—like properties, a concept rooted in Takahashi and Yamanaka’s work, where human adult dermal fibroblasts were first reprogrammed into stem cell-like cells.^170^ Dermal fibroblasts also exhibit plasticity, allowing for direct transdifferentiation into other cell types, such as adipocytes or neural cells, under specific conditions.^171,172^ These discoveries are paving the way for regenerative therapies, including engineered skin grafts and treatments for degenerative skin diseases.

The skin also functions as an active immune organ, where immune cells like macrophages and T cells play critical roles in modulating inflammation and repair. Macrophage plasticity, influenced by signals from keratinocytes, adipocytes, and innate immune cells, is essential for wound healing.^173^ In diabetic wounds, impaired transition from proinflammatory M1 to prohealing M2 macrophages delays healing.^174,175^ Similarly, T cell dysfunction, driven by epigenetic changes or misregulated FOXP3 and CD18, contributes to autoimmune skin disorders.^176^ Together, these insights reveal the skin as a highly adaptive organ—capable of reprogramming, regeneration, and immune coordination—making it a powerful model for studying plasticity and a promising target for therapeutic innovation.

### C. Plasticity of the nervous system

Neurons and glial cells, which make up the CNS and PNS, exhibit remarkable plasticity. This cellular adaptability allows them to respond dynamically to developmental cues, injury, degeneration, and regeneration throughout life. In the CNS, neuronal plasticity is exemplified by the role of intracellular calcium (Ca^2+^) in regulating growth cone activity, synaptic outgrowth, and synaptic plasticity. Long-term potentiation and long-term depression—key mechanisms of synaptic modification—are tightly linked to fluctuations in Ca^2+^ levels.^177,178^ In aging mammals, long-term depression has been associated with synaptic loss and cognitive decline, highlighting Ca^2+^-dependent pathways as potential therapeutic targets to prevent synapse degeneration.^178^ In the PNS, Schwann cells demonstrate injury-induced plasticity by shifting from a myelinating role to one that supports axonal regeneration. Upon nerve damage, these cells undergo genetic reprogramming to initiate Wallerian degeneration, clear myelin debris, recruit macrophages, and promote angiogenesis.^179^ They also secrete trophic factors such as nerve growth factor and BDNF and proliferate to generate promyelinating progeny.^179,180^ These regenerative capabilities make Schwann cells promising targets for therapies aimed at enhancing nerve repair.^181–183^

Sympathetic neuronal plasticity also responds to immune activation and inflammation. In lymphoid tissues, axonal degeneration and regeneration are modulated by neurotrophins and semaphorins during inflammatory responses^184,185^ Recent findings reveal a feedback loop in which inflammation-activated *γδ* T cells promote axon degeneration, impair norepinephrine release, and enhance T cell activation.^186^ Additionally, during fasting, sympathetic plasticity supports glucose homeostasis by strengthening adrenal synapses via neuropeptide Y release.^187^ Mild stress or stimulation can trigger hormetic responses in the nervous system, promoting resilience and neuroprotection. These adaptive responses leverage neural plasticity to help the system cope with repeated challenges.^188^ In humans, hormesis appears to support cytoprotective mechanisms and may influence NSC proliferation and differentiation.^189–192^ Adaptive hormetic responses may regulate NSC proliferation and differentiation.^193^ In the adult mammalian brain, NSC niches such as the SVZ and subgranular zone are essential for neurogenesis.^80,194^ The SVZ, in particular, receives trophic signals from cerebrospinal fluid to regulate NSC maintenance. Following brain injury, such as stroke, VEGF signaling promotes vascular repair and the release of CXCL12, which recruits neuronal precursors from the SVZ to aid in neural recovery.^195^

Brain plasticity is continuously shaped by environmental stimuli. For instance, visual training increases blood oxygenation in the visual cortex and fosters new synaptic connections.^196^ Structural and molecular changes—such as dendritic remodeling, gap junction alterations, dopamine receptor regulation, enzymatic shifts, and calcium signaling—underlie this plasticity.^197–199^ Chronic exposure to psychostimulants can induce both structural and synaptic adaptations, which may be mitigated by dopaminergic inhibitors.^200^ Extreme plasticity is observed in cases of brain injury or surgical interventions like hemispherectomy, where the remaining hemisphere can reorganize to assume functions such as language and motor control. This capacity is especially pronounced in children under 6 years of age.^201–203^ While neurogenesis appears largely restricted to early development in humans,^204^ some evidence suggests limited adult neurogenesis under experimental conditions. For example, studies using bromodeoxyuridine (BrdU) labeling and ^14^C birth dating—leveraging nuclear bomb test fallout—have provided indirect evidence of adult neurogenesis, estimating that approximately 0.004% of dentate gyrus neurons are renewed daily.^205–207^ However, these methods have limitations, including difficulty identifying young neurons and progenitors. Immunostaining studies have failed to detect markers of young neurons (eg, DCX^+^TUJ1^+^ and DCX^+^PSA^−^NCAM^+^) in adult hippocampal tissue, challenging the notion of significant adult neurogenesis.^208^ Indirect evidence of plausible neurogenesis has been noted in BrdU-injected adult human brain. Limitations in the methodological approach including but not limited to the use of postmortem tissues complicates data interpretation.^209^ ^14^C birth dating of NeuN^+^ nuclei, a result of nuclear bomb testing, provided interesting insight into neurogenesis in the adult human brain. Encouragingly, it is estimated that 0.004% of neurons in the dentate gyrus are renewed each day. Adult-born neurons appear to have shorter lifespans than neurons produced during early development. ^14^C birth dating, however, is limited in its ability to identify young neurons and progenitors. In disagreement with the findings from the ^14^C birth dating observations, direct immunostaining studies show that DCX^+^TUJ1^+^ young neurons present at 3 weeks of human age were not detectable in adult granule cell layer, nor were DCX^+^PSA^−^NCAM^+^ young neurons present in granule cell layer of adult hippocampi.^208,210^ Despite these uncertainties, physical exercise has emerged as a promising nonpharmacological strategy to enhance neuronal plasticity, likely through BDNF upregulation.^211^ While definitive conclusions remain elusive,^212^ the possibility of adult neurogenesis in the human brain remains open, warranting further investigation into its extent and functional relevance.

### D. Muscle plasticity

Skeletal muscle is a highly plastic tissue, constantly remodeling in response to changes in physical activity and mechanical load. Depending on the demands placed on the body, muscle fibers can either grow (hypertrophy) or shrink (atrophy). After physical exertion, muscles typically undergo transcriptionally regulated hypertrophy, marked by an increase in sarcomere number and fiber diameter.^213,214^ In contrast, prolonged inactivity—such as during bed rest or spaceflight—leads to muscle atrophy, characterized by reduced cross-sectional area, decreased voluntary contraction strength, and diminished oxidative capacity due to capillary loss.^215^ This balance between hypertrophy and atrophy is partly regulated by the microvascular endothelium through Dll4-Notch2 signaling, which influences catabolic pathways.^216^ In pathological conditions like cerebral palsy, children with muscle contractures show longer sarcomere lengths and a reduced number of muscle stem cells (MuSCs) compared with healthy individuals.^217^ Beyond structural changes, skeletal muscle also undergoes metabolic adaptations in response to various factors, including exercise, dietary fat intake, hypoxia, thermal stress, and aging. These adaptations enhance the muscle’s ability to meet the body’s changing energy demands.^215,218^ Such metabolic plasticity allows the body to adapt to the physical demands of its environment.

At the neuromuscular junction (NMJ), plasticity is evident in the regulation of motoneuron excitability and synaptic vesicle release, primarily mediated by acetylcholine signaling. This process converts electrical signals into muscle contractions. NMJ plasticity allows for both structural and functional adjustments based on activity levels: increased use enhances synaptic transmission, while disuse impairs it and reduces muscle performance.^219^ Contrary to earlier beliefs that NMJs remain stable in adulthood, research shows they continue to remodel throughout life. However, this remodeling often leads to a decline in neuromuscular transmission and function with age.^220^

In neuromuscular disorders such as myasthenia gravis, the reduced long-term efficacy of cholinesterase inhibitors may be linked to compensatory homeostatic plasticity triggered by altered postsynaptic potentials.^221^ Similarly, in conditions like amyotrophic lateral sclerosis (ALS), Lambert-Eaton myasthenic syndrome, and age-related sarcopenia, impaired NMJ plasticity contributes to progressive muscle degeneration, reduced muscle mass, and increased fatigue.^222^

### E. Adipose plasticity

Adipose tissue, primarily made up of adipocytes, is a highly plastic and dynamic tissue best known for storing fats and lipids for energy. However, its functions extend far beyond energy storage—it also plays key roles in regulating immune responses, appetite, thermoregulation, and insulin sensitivity. There are several types of adipose tissue, each with distinct functions:

- White adipose tissue (WAT) stores energy in the form of triglycerides and releases free fatty acids when needed.^223^ It can expand through
  - Hypertrophy (increased cell size)
  - Hyperplasia (increased cell number).

This expansion depends on factors like tissue location and lipid availability.^224^ However, excessive WAT accumulation can lead to lipotoxicity, impairing insulin function and increasing the risk of diabetes.

- Brown adipose tissue and beige adipose tissue specialize in thermogenesis, generating heat by burning energy through uncoupling protein 1.^225,226^ A key example of adipose plasticity is the “browning” of WAT, where cold exposure triggers the formation of beige adipocytes. These can arise from the following:
  - Differentiation of precursors
  - Proliferation of existing beige cells
  - Reversible conversion of white adipocytes

This transformation is regulated by PPARγ and SIRT1, enabling the body to adapt to cold environments.^227,228^

- Pink adipose tissue is a recently identified type that forms during pregnancy and lactation. It results from the transdifferentiation of WAT into milk-producing mammary alveolar cells.^229,230^

Adipose tissue plasticity is also being explored for therapeutic applications. *β*3 adrenergic receptor agonists are under investigation for their ability to stimulate fatty acid oxidation in diabetic and obese patients.^223,231^ Moreover, adipose-derived stromal cells have shown the potential to differentiate into various cell types, including cardiomyocyte-like cells,^232^ osteoblasts,^233^ insulin-producing cells,^234^ fibroblasts,^235^ and vascular endothelial cells.^236^

### F. Tissue plasticity in response to organ failure

When an organ begins to fail, the body can activate compensatory mechanisms at both the cellular and tissue levels to maintain homeostasis. This highlights the remarkable plasticity not only of individual cells but also of entire tissues and organs. A striking example is seen in kidney failure, where the skin partially compensates for the loss of renal function. Normally, the kidneys regulate water and salt balance, remove metabolic waste, and maintain acid—base homeostasis.^237^ However, in patients with kidney failure, nitrogenous waste products such as urea have been detected on the skin surface,^238–241^ suggesting that sweat glands can assist in waste excretion. Sweat contains substances like NaCl, urea, and ammonia, indicating that sweat glands serve functions beyond thermoregulation.^242^ Anatomically, sweat glands and kidney nephrons share similarities, including a filtration component and a duct system for waste removal.^243^ Historically, diaphoresis (sweating) has been used therapeutically to eliminate bodily impurities. Supporting this, studies show that serum urea levels in dialysis patients decrease during summer months, likely due to increased sweating.^242,244^ In fact, urea concentrations in sweat can be up to three times higher than in serum, and up to 50 times higher in uremic patients.^238^ This suggests a potential therapeutic strategy: enhancing sweat gland function to aid in urea excretion. Pilocarpine hydrochloride, a muscarinic receptor agonist, can induce sweating and may be used to promote this process.^243^ Developing pilocarpine-mimetic drugs could harness sweat gland plasticity to support waste removal in kidney failure. Beyond compensation, the body also activates endogenous regenerative mechanisms to preserve function and minimize damage. In partially damaged organs, surviving cells may undergo hypertrophy (increase in size) or hyperplasia (increase in number) to compensate for lost tissue.^245–247^ For example, in the kidneys, remaining nephrons enlarge to maintain filtration capacity after injury.^248–250^ Similarly, in the brain, some patients show spontaneous recovery after ischemic stroke, even without clinical intervention.^251^ This recovery is partly driven by NSC niches in the SVZ and subgranular zone. These regions respond to VEGF and CXCL2 signaling, which recruit neuronal precursor cells to support neural repair.^195^

### G. Neoplasticity: plasticity and stemness gone awry

Cellular plasticity and stemness are fundamental biological properties that allow cells to adapt to environmental cues, regenerate damaged tissues, differentiate into specialized cell types, and maintain tissue homeostasis.^55–58,60,64^ However, when these tightly regulated processes become dysregulated, they can give rise to abnormal cell growth and tissue architecture—hallmarks of neoplasia. Neoplastic growth, which can occur in virtually any tissue or organ, may be benign or malignant. Malignant tumors are particularly dangerous due to their ability to invade surrounding tissues and metastasize. Many cancers originate from stem cells or highly plastic progenitor cells, which, under normal conditions, are responsible for tissue renewal and repair. However, even differentiated cells can acquire stem-like properties under certain pathological conditions, contributing to tumor initiation and progression.^252,253^

In healthy tissues, stem cell niches maintain a delicate balance between dormancy and activation. These niches produce progenitor cells in response to specific extracellular signals, ensuring tissue integrity and repair.^79^ Immune cells within these microenvironments play a dual role: they help eliminate cells with precancerous changes, but chronic inflammation—often due to persistent injury or infection—can disrupt this balance. This altered signaling environment may exhaust stem cell reserves and promote tumorigenesis.^254,255^ The immune system’s paradoxical role in cancer is well documented. While it can suppress early tumor formation by clearing abnormal cells, it can also support tumor growth once cancer is established by creating an immunosuppressive microenvironment.^256,257^

Additional factors that drive neoplastic transformation include genetic mutations, environmental stress, and proangiogenic signaling.^255^ Chronic inflammation, which mimics the tissue repair process, can stimulate angiogenesis—an essential step in tumor development.^258, 259^ Tumors require a blood supply to grow beyond a minimal size (approximately 1 mm in diameter), and they achieve this by either inducing new blood vessel formation or hijacking existing vasculature.^260,261^ Key angiogenic factors involved in this process include VEGF, TGF-*β*, TGF-*α*, fibroblast growth factor (FGF), granulocyte-colony—stimulating factor, and tumor necrosis factor-*α*.^262^ These molecules help neoplastic cells survive under hypoxic conditions and support continued tumor growth and invasion.^33^

Neoplastic growth is not solely driven by the self-regulation of cancerous cells—it also depends heavily on the surrounding tumor microenvironment. Various noncancerous cells contribute to tumor development and progression by providing structural support, signaling molecules, and metabolic resources. For instance, endothelial cells play a critical role in angiogenesis, supplying tumors with oxygen, nutrients, and paracrine factors that promote growth and modulate immune responses.^258,259,263^ Other components of the tumor microenvironment, such as cancer-associated fibroblasts and infiltrating immune cells, can either suppress or promote tumor growth depending on the context and signaling dynamics.^252,253,264–267^

While many neoplastic cells originate from stem cells due to their inherent plasticity and self-renewal capacity, normal somatic cells can also become cancerous. This transformation often results from genetic mutations or disruptions in the normal cell cycle, frequently triggered by carcinogenic exposures.^268–272^ These mutations contribute to the acquisition of the well-known “Hallmarks of Cancer,” including genomic instability, resistance to apoptosis, sustained proliferation, and angiogenesis.^252,253^ Importantly, tumor progression is not always linear. Owing to high mutation rates and clonal diversity, each tumor lineage may evolve differently, requiring individual tracking to fully understand its behavior and therapeutic vulnerabilities.^273,274^ Another pathway to neoplastic transformation involves disruptions in the cell cycle, such as endomitosis—a process in which cells replicate their DNA without completing mitosis, resulting in polyploid cells with multiple nuclei.^275^ Polyploidy is frequently observed in human tumors and is associated with chemoresistance.^276^ While polyploid cells can arise naturally—such as during liver development—they may also result from DNA damage caused by carcinogens.^277–279^ These polyploid cells can undergo a rare form of division known as neosis, producing smaller, proliferative daughter cells that contribute to tumor growth.^280^ This process illustrates how even differentiated somatic cells, under stress or mutation, can revert to a proliferative, stem-like state—fueling neoplastic transformation and tumor progression. Thus, while cell plasticity and stemness are essential for tissue repair and regeneration, their dysregulation can lead to cancer. Stem-like cells, chronic inflammation, immune modulation, and angiogenesis, all contribute to the transformation of normal tissue into malignant tumors. Understanding these mechanisms is crucial for developing targeted therapies that can disrupt cancer at its roots.

## IV. Cell-state and cell-fate regulation

In early mammalian embryogenesis, the establishment of cell fates and tissue patterning is orchestrated through a combination of cellular plasticity, positional cues, and cell—cell signaling. These mechanisms guide cells toward specific lineages, ensuring proper development and tissue organization.^21,61^ At the heart of this process lies the distinction between cell state and cell fate—a foundational concept in developmental biology and regenerative medicine. A cell state refers to a cell’s current functional and molecular profile, including gene expression patterns, protein production, morphology, motility, and behavior.^281^ In contrast, a change in cell fate involves a more profound transformation: the loss of the original identity and the acquisition of new genetic markers, structure, and function that define a different cell type. Cell states and fates can shift naturally during development or disease or be manipulated experimentally through cellular reprogramming. This plasticity—enabled by stemness and environmental responsiveness—allows cells to adapt, regenerate, or even reverse their differentiation under certain conditions.

In simpler organisms like *Caenorhabditis elegans*, cell fate decisions are well characterized and often follow a binary model, where 2 competing signaling pathways determine a cell’s developmental trajectory.^282–284^ Activation of a pathway commits the cell to a specific fate, while suppression leads to an alternative outcome. However, in more complex organisms such as mammals, cell fate decisions are not strictly binary. Advances in single-cell transcriptomics have revealed that populations once thought to be homogeneous actually exhibit significant heterogeneity and plasticity.^285^ Moreover, analyses of gene expression dynamics and pseudotime trajectories in differentiating cells have uncovered the existence of transitional states—intermediate phases where cells are partially differentiated but not yet committed to a final fate.^286^ These transitional cells may even revert to earlier states, highlighting the fluidity of cell identity. Understanding these nuances is critical for advancing regenerative therapies and developmental biology. Grasping how cells transition between states and fates enables better manipulation of cell behavior for therapeutic purposes or understand how dysregulation leads to a wide range of diseases.

Understanding how cells transition between different states and fates is central to the science of tissue development, regeneration, and disease. Recent breakthroughs in single-cell transcriptomics, lineage tracing, and fate mapping have revolutionized this field by enabling researchers to observe cellular behavior at unprecedented resolution.^287–291^ These technologies have uncovered the existence of transient and intermediate cell states, revealing that cells with distinct transcriptional profiles can still converge on a shared fate identity.^292^ This complexity is shaped by multiple factors, including the tissue microenvironment, regional signaling cues, and the cell’s current state.^293^ These influences contribute to transcriptional heterogeneity, even within seemingly uniform cell populations. For example, HSCs exhibit multipotency during postnatal development, giving rise to diverse lineages such as myeloid, erythroid, and lymphoid cells.^294^ However, disruptions in cell cycle regulation, ECM interactions, or transcriptional control can derail this process, leading to diseases like cancerous myelofibrosis.^295^ Importantly, changes in cell state and fate are not limited to stem cells. In the dermis, for instance, specific fibroblast subpopulations such as Prrx1+ fibroblasts are involved in ECM production during wound healing and in the stromal response to tumors.^296,297^ These fate decisions are often regulated by microRNAs, which act as fine-tuners of gene expression during tissue repair and regeneration.^298–300^ In the context of cardiovascular disease, microRNAs also play pivotal roles. For example, miR-21 has been implicated in the transdifferentiation of cardiac fibroblasts into myofibroblasts, a process that contributes to fibrosis and heart failure.^301–304^ Similarly, during atherosclerosis, vascular smooth muscle cells undergo multiple transitional states, adopting inflammatory macrophage-like or myofibroblast-like phenotypes that contribute to plaque formation.^305,306^ These insights—made possible by high-resolution single-cell sequencing and advanced bioinformatic tools—underscore the importance of understanding cell state dynamics. They reveal that cell fate is not a fixed endpoint but a dynamic adaptive process influenced by internal programs and external signals. This evolving view is reshaping how we approach regenerative medicine, disease modeling, and therapeutic reprogramming.

Pioneer transcription factors are a unique class of proteins that play a foundational role in regulating cell fate. Unlike most transcription factors, which require open chromatin to access DNA, pioneer factors can bind directly to their target DNA sequences within closed or condensed chromatin. This ability allows them to “unlock” silenced regions of the genome, making them accessible to other transcriptional machinery and initiating major changes in gene expression. The significance of pioneer factors became widely recognized with the discovery of the OSK factors—Oct3/4, Sox2, and Klf4—by Takahashi and Yamanaka in 2006.^54^ These factors were shown to reprogram somatic cells into iPSCs, a breakthrough that transformed regenerative medicine. Since then, additional pioneer factors have been identified, including FoxA, Ascl1, Pax7, PU.1, Gata4, and p53.^307,308^ These transcription factors function by suppressing somatic enhancers and activating pluripotency-associated enhancers in previously inaccessible chromosomal regions.^309,310^ Through this mechanism, they initiate the reprogramming of cell identity. By manipulating pioneer factors and other transcriptional regulators, scientists can reprogram cells in 2 main ways:

- Indirect reprogramming, where cells are first converted into a pluripotent state before being directed toward a specific lineage.
- Direct reprogramming (transdifferentiation), where one somatic cell type is converted directly into another without passing through a pluripotent stage.

Both approaches hold significant therapeutic potential, offering strategies for tissue regeneration, disease modeling, and personalized medicine.^311^ Altering cell fates through indirect (pluripotent intermediate) or direct (transdifferentiation) can be harnessed to drive therapeutic changes (detailed in Indirect reprogramming for cell-based and tissue-based therapies and Direct reprogramming for cell-based and tissue-based therapies).

## V. Drug screening and disease modeling

**Fig. 6.**
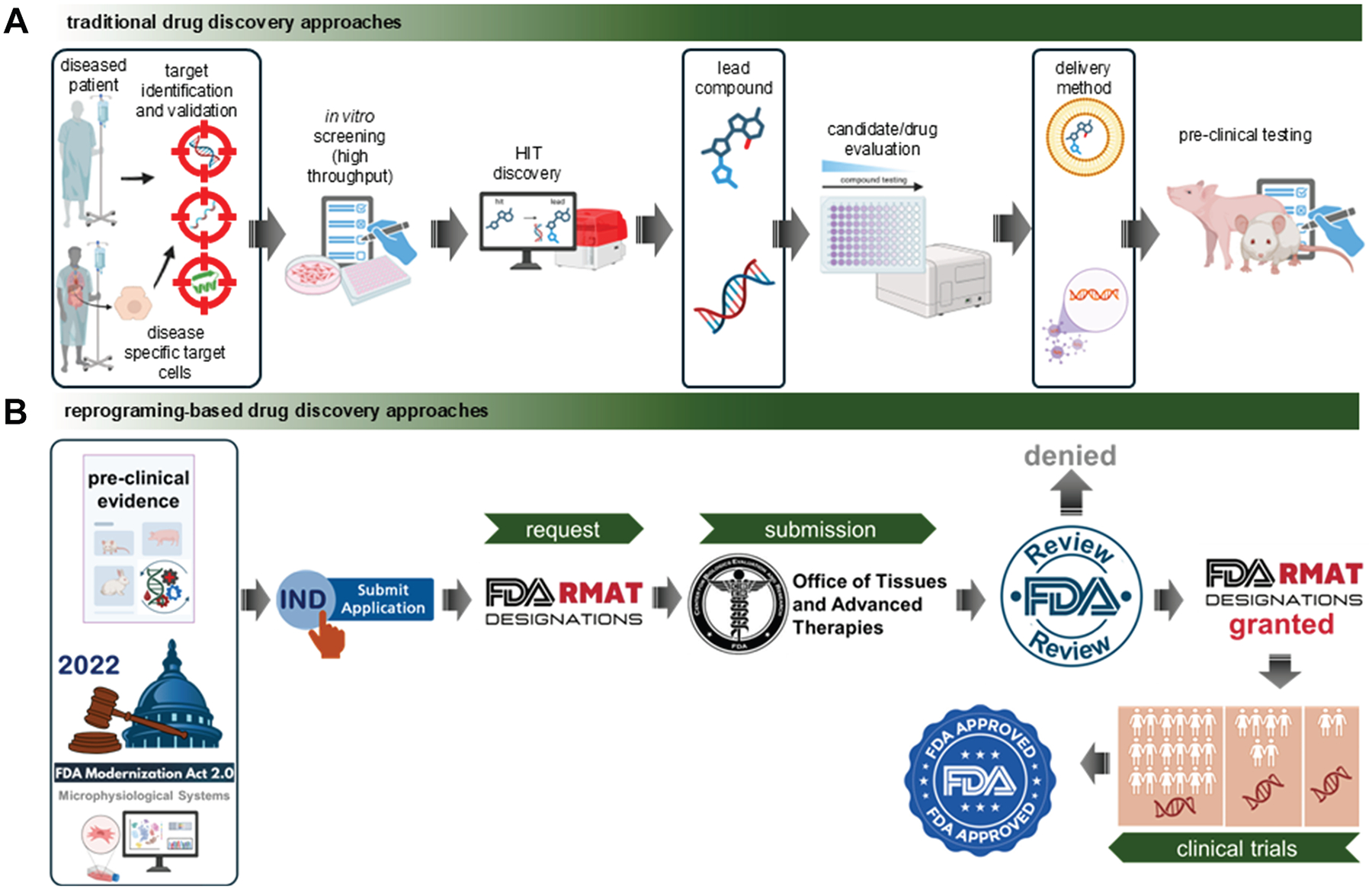
Traditional and reprogramming-based drug discovery approaches. Traditional drug discovery has relied on in vitro screening to identify potential hit compounds that are likely to have the desired effect. These compounds are evaluated by in vitro assays before preclinical testing in animal models. However, the future is shifting toward complimentary methods like MPSs, computer modeling, and AI-driven simulations. While these emerging technologies are transforming drug discovery, in many cases, animal testing remains a necessary component for validating complex biological responses that current models cannot fully replicate. These approaches are increasingly used together, with animal studies providing critical insights where alternative methods still face limitations, ensuring both scientific rigor and ethical progress. Reprogramming of cells and tissues is revolutionizing drug discovery by enabling the creation of highly accurate, patient-specific models of human disease. These models allow the study of disease mechanisms and screen potential therapies in a human-relevant context, improving predictive power and reducing reliance on animal models. At the same time, advances in cellular and tissue reprogramming—particularly in vivo—are giving rise to a new class of therapeutics, where cells within the body are directly converted or repurposed to restore function, offering transformative potential for regenerative medicine and chronic disease treatment. Gene and small molecule therapies aiming at tissue repair and regeneration have been designated by the FDA as RMAT. Confidence in reprogramming-based drug discovery has led to legislative changes in the US FDA to bypass the use of preclinical animal testing before FDA review of RMAT candidates for clinical trials. HIT, compound that displays desired biological activity towards a drug target and reproduces this activity when retested; IND, investigational new drug.

The landscape of drug discovery and disease modeling is undergoing a profound transformation, accelerated by the passage of the FDA Modernization Act 2.0. This landmark legislation, enacted in late 2022, officially removed the federal requirement for animal testing in preclinical drug development, allowing the use of scientifically validated alternatives such as cell-based assays, organoids, and computational models (Fig. 6).^312^ This shift reflects growing recognition that traditional animal models often fail to accurately predict human responses, with more than 90% of drugs that succeed in animal trials ultimately failing in human clinical studies due to issues of safety or efficacy. In this evolving regulatory and scientific environment, cell and tissue reprogramming has emerged as a powerful tool in pharmacological research and therapeutic development. By harnessing the plasticity of cells, reprogramming strategies enable the transformation of one cell type into another, offering novel ways to model diseases and test drugs in systems that closely mimic human biology. These approaches go beyond symptom management, targeting the root causes of disease at the cellular level.

**Fig. 7.**
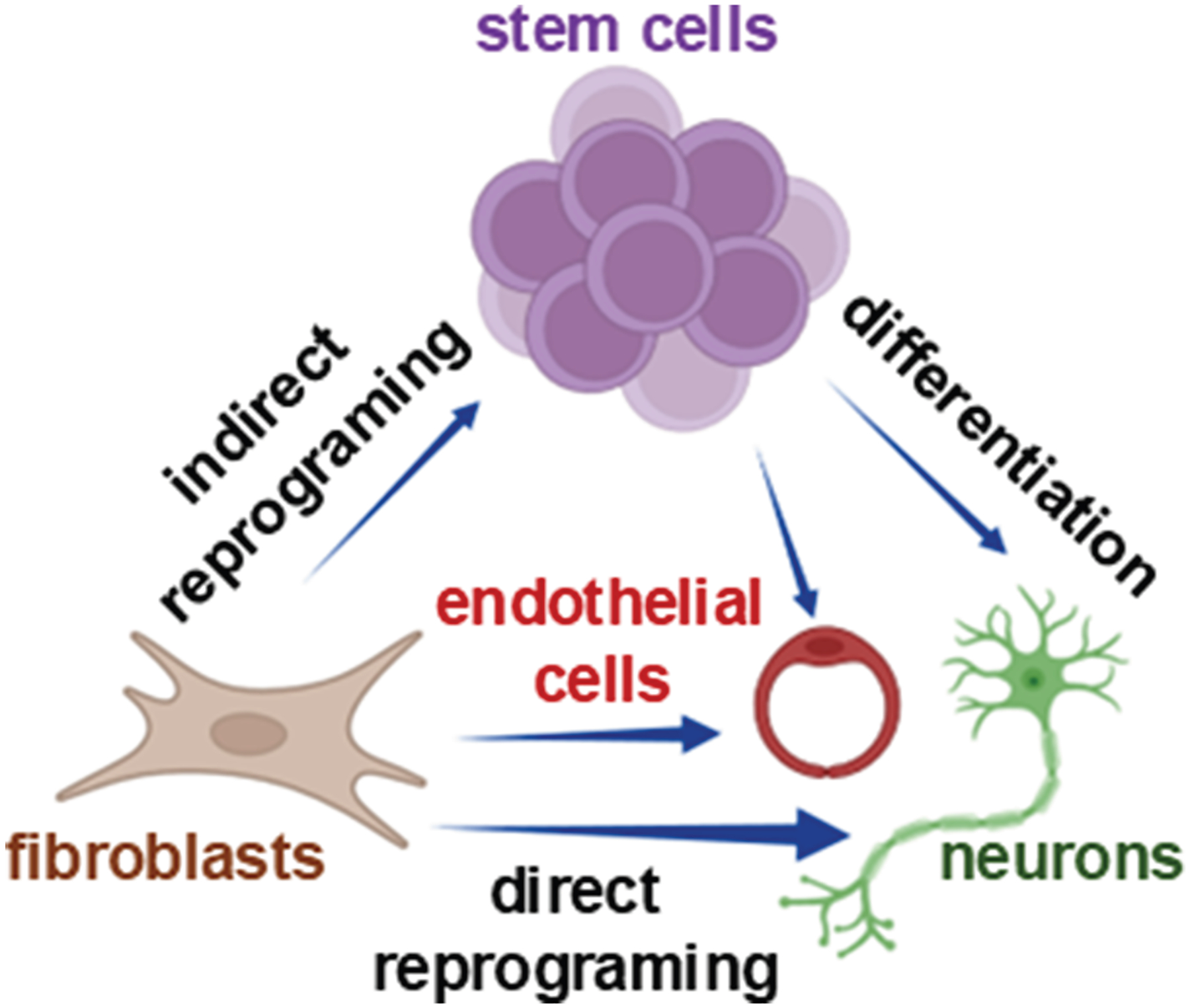
Indirect and direct reprogramming pathways. Indirect reprogramming involves the conversion of mature cells (eg, fibroblasts) into an intermediate iPSC. iPSCs are then treated with reprogramming factors to differentiate into a desired target cell type (eg, endothelial cells and neurons). Direct reprogramming, or transdifferentiation, methods harness reprogramming factors to convert mature cells into target cell types without inducing a pluripotent intermediate state. Direct reprogramming is considered safer than indirect reprogramming because it bypasses the generation of intermediary pluripotent stem cells, which are inherently unstable and difficult to control in vivo. This reduces the risk of unintended differentiation, tumor formation, or other adverse effects, making direct reprogramming a more targeted and predictable therapeutic strategy.

Reprogramming techniques fall into 2 main categories: indirect and direct. Indirect reprogramming involves converting mature cells into iPSCs, which can then be differentiated into virtually any cell type. This method provides a versatile platform for modeling complex diseases and screening potential therapies. In contrast, direct reprogramming—previously discussed as transdifferentiation—bypasses the pluripotent stage, converting one specialized cell type directly into another or into a functional intermediate state (Fig. 7). This approach offers a faster and potentially safer route to generating disease-relevant cells. These reprogrammed cells are increasingly used to build ex vivo human disease models, which offer a more accurate and ethical alternative to animal testing. Such models not only improve the predictive power of drug screening but also align with the FDA’s new regulatory framework that encourages the adoption of new approach methodologies.^313^ As research continues to uncover the mechanisms behind cellular reprogramming and refine these technologies, the potential to develop highly targeted, effective, and personalized therapies is expanding rapidly. Together with supportive policy changes, these scientific advances are ushering in a new era of precision medicine—one that is more human-relevant, efficient, and ethically responsible.

The FDA Modernization Act 2.0 authorizes the use of nonanimal alternatives—such as in vitro and in silico models—for investigational new drug applications, effectively removing the statutory requirement for animal testing before human clinical trials. This marks the ushering in of a new horizon in drug development by pioneering a shift away from traditional animal testing. In a landmark move, the agency is now endorsing a suite of validated alternative methodologies that promise to revolutionize how drugs are evaluated for safety and efficacy. Leading this transformation are microphysiological systems (MPSs)—advanced models designed to emulate specific aspects of human physiology, though they often lack components related to immune surveillance. These systems are rapidly emerging as the gold standard for drug validation, offering a more accurate, ethical, and efficient approach to testing. MPS technologies are being developed to support the use of laboratory-grown human organoids and organ-on-chip platforms.^314^ By using patient-derived cells, it is possible to reprogram tissues that carry the genetic and epigenetic signatures of specific diseases.^315,316^ This allows MPS to model not just healthy physiology but also disease-specific pathophysiology, making them powerful tools for studying complex conditions like cancer, Alzheimer disease (AD), or rare genetic disorders.^317,318^ These advanced tools, especially when incorporating immune surveillance, closely mimic human biological functions and disease-specific processes, allowing researchers to evaluate drug responses in a physiologically relevant context. This paradigm shift not only enhances predictive accuracy but also accelerates the path from discovery to treatment—marking a bold step into the future of medicine. Collectively, these diverse applications underscore tissue reprogramming as a transformative force in modern biomedicine—bridging regenerative therapy, disease modeling, and precision drug development across a wide spectrum of pathologies.

From a drug discovery perspective, cellular and tissue reprogramming represents a targeted therapeutic approach that combines gene therapy and/or small molecule treatments to alter cell identity and function. According to current regulatory standards, these therapies fall under the category of advanced therapy medicinal products (ATMPs)—a diverse class of biopharmaceuticals that includes gene, cell, and tissue-engineered therapies. Although still in the early stages of clinical adoption, reprogramming-based therapies are beginning to enter the market. In recent years, the number of approved ATMPs has grown rapidly, particularly for rare cancers, genetic disorders, and other debilitating diseases. Most of these therapies have received conditional authorization, reflecting both their promise and the need for further validation. However, they are among the most expensive treatments available, due in part to the complex and individualized manufacturing processes involved, such as autologous cell production.^319^ This complexity presents significant challenges for scaling these therapies for widespread clinical use. To overcome these barriers, nonviral, direct in vivo tissue reprogramming (Table 1)^112,140,172,320–352^ is emerging as a promising alternative. This approach requires minimal infrastructure and is expected to be more scalable and cost-effective. As healthcare systems face increasing pressure from the rising prevalence of chronic diseases, ensuring that ATMPs are economically sustainable is critical to making these innovations broadly accessible and viable for long-term use.

Tissue reprogramming is also fundamentally reshaping the drug development pipeline by enabling the creation of dynamic, disease-relevant models that more accurately reflect human pathophysiology. This shift is particularly impactful in metabolic diseases such as obesity and diabetes mellitus, where chronic inflammation and cellular dysfunction disrupt systemic homeostasis. For example, reprogramming proinflammatory macrophages into anti-inflammatory phenotypes or converting white fat precursors into beige adipocytes offers novel therapeutic strategies.^353^ In diabetes, converting nonendocrine pancreatic cells into insulin-producing *β*-like cells provides a promising avenue for restoring glycemic control, including in cases of drug-induced or cancer therapy—related hyperglycemia.^354^ Beyond metabolic disease, tissue reprogramming offers critical insights into immune evasion in oncology. In pancreatic ductal adenocarcinoma, for instance, therapeutic pressure can reprogram the tumor microenvironment into an immunosuppressive state. Activation of the STING pathway may paradoxically promote IL-35—producing regulatory B cells, which suppress NK cell activity, thereby undermining the efficacy of immunotherapies.^355–357^

Tissue reprogramming is also emerging as a powerful tool in the treatment of ischemic diseases. Recent advances show that dermal fibroblasts can be reprogrammed into vasculogenic fibroblasts (VFs) through modulation of microRNAs (eg, miR-200b) or transcription factors (eg, Etv2, Foxc2, and Fli1) delivered via tissue nanotransfection (TNT). These VFs exhibit endothelial-like properties, contributing to neovascularization and enhancing tissue perfusion and wound healing in ischemic and diabetic contexts. Mechanistically, this reprogramming is driven by TET-mediated epigenetic remodeling, which is suppressed in diabetes but can be restored through TNT, leading to increased 5-hydroxymethylcytosine levels and reactivation of endothelial gene expression.^140^ These findings not only highlight the therapeutic potential of reprogramming in vascular regeneration but also establish reprogrammed fibroblasts as a novel platform for screening proangiogenic compounds and understanding ischemic tissue repair mechanisms.

Another major advancement is the ability to reprogram adult somatic cells into iPSCs. These patient-specific cell lines can be differentiated into various tissue types, enabling personalized disease modeling for conditions such as neurodegenerative diseases, cardiac disorders, and rare genetic syndromes. This allows for tailored drug screening, where therapies can be tested on cells genetically identical to the patient.^358–363^ Reprogrammed cells are also used to generate organoids—miniature, 3-dimensional (3D) models that mimic the structure and function of human organs. These organoids provide a more accurate representation of human physiology than traditional 2-dimensional (2D) cultures, enabling high-throughput drug screening in a context that better predicts in vivo responses.^364–367^ Together, these advances position tissue reprogramming as a transformative force in modern biomedicine, bridging regenerative therapy, disease modeling, and precision drug development across a wide spectrum of pathologies.

### A. Indirect reprogramming

In 1998, Thomson et al^111^ made a landmark contribution to stem cell science by successfully deriving hESCs from blastocysts using embryos obtained through in vitro fertilization. While this breakthrough opened new frontiers in regenerative medicine, it also ignited intense ethical and religious debates over the use of human embryos in research. These concerns led to a federal ban in the United States on the use of human embryos in government-funded research, a restriction that has been in place since 1996.^368,369^ A major turning point came in 2006, when Shinya Yamanaka and Kazutoshi Takahashi identified a set of 4 transcription factors—Oct3/4, Sox2, c-Myc, and Klf4 (collectively known as OSKM)—that could reprogram adult somatic cells back into a pluripotent state, creating what are now known as iPSCs.^54^ Just a year later, they demonstrated this technique using human adult dermal fibroblasts.^170^ Around the same time, Thomson’s group developed an alternative reprogramming cocktail—Oct4, Sox2, Nanog, and Lin28—to generate iPSCs from human fibroblasts.^370^ Given the complexity of mammalian gene regulation, which involves more than 1600 transcription factors,^371^ the ability to reprogram a fully differentiated somatic cell using just 4 factors is extraordinary. In the protocol by Yamanaka, the transcription factor c-Myc plays a pivotal role by loosening tightly packed chromatin, thereby enabling the OSKM factors to initiate a mesenchymal-to-epithelial transition and promote cell proliferation.^372^ This discovery fundamentally challenged the classical view of cell differentiation described by Conrad Waddington’s epigenetic landscape, which portrays cell fate as a 1-way trajectory toward increasing specialization.^373^ The ability of OSKM factors to reverse this process—reprogramming differentiated cells to a pluripotent state—represents a paradigm shift in our understanding of cellular identity and plasticity.

Since Yamanaka’s groundbreaking discovery, there has been remarkable progress in the ex vivo and in vitro indirect reprogramming of various adult somatic cell types into iPSCs.^374–377^ This technology has significantly advanced disease modeling and drug screening, enabling researchers to study cell—cell interactions and molecular signaling in scalable culture systems that closely mimic the function and transcriptomic profile of primary human cells.^378^ One of the key advantages of iPSC-based models is that they bypass the ethical concerns associated with ESCs and eliminate species-specific differences that often limit the translational relevance of animal models.^369,379^ The typical iPSC workflow for drug discovery involves several steps: collecting patient-derived somatic cells, expanding them in culture, reprogramming them into iPSCs, selecting and expanding iPSC clones, differentiating them into disease-relevant cell types, and finally characterizing disease phenotypes and drug responses through epigenetic profiling, gene expression analysis, and functional assays.^380^

Indirect reprogramming via iPSCs requires ex vivo manipulation, where cells are first reverted to a pluripotent state and then differentiated into the desired lineage before being introduced into the recipient. However, this process raises safety concerns, particularly regarding genotoxicity and insertional mutagenesis when viral vectors are used for gene delivery.^381,382^ In response, development of nonintegrating and nonviral reprogramming methods to mitigate these risks is warranted. Another challenge is the potential for immune rejection of iPSC-derived cell therapies. To address this, efforts are underway to establish iPSC haplobanks—repositories of iPSCs from human leukocyte antigen (HLA)—homozygous donors—to improve compatibility and reduce immune responses in clinical applications.^381,383,384^ Despite these challenges, iPSC-based systems offer powerful platforms for in vitro pharmacological studies, including pharmacokinetics, pharmacodynamics, and toxicity testing, while preserving the genetic background of disease throughout the differentiation process.^385^ These models have been integrated into advanced platforms such as organ-on-a-chip, multiorgan systems, and 3D organoids, enabling more physiologically relevant drug testing. For example:

- iPSC-derived cardiac models have been used to assess the cardiotoxicity of antidepressants.^386^
- Hepatopancreatic organoid systems have helped elucidate metabolic interactions and molecular networks.^387^
- iPSC-based platforms have supported therapeutic drug development against malaria parasites.^388^
- miRNA biomarkers have been identified for doxorubicin-induced cardiomyopathy using iPSC-derived cardiac tissues.^389^

Together, these advances underscore the transformative potential of iPSC technology in personalized medicine, regenerative therapy, and precision drug development.

A landmark early application of iPSC technology in disease modeling focused on spinal muscular atrophy (SMA), a severe inherited neurological disorder with high infant mortality.^390^ Ebert et al^391^ reprogrammed fibroblasts from a patient with SMA and an unaffected parent into iPSCs using lentiviral delivery of the transcription factors OCT4, SOX2, NANOG, and LIN28.^391^ These iPSCs were then differentiated into motor neurons, revealing a reduced number of motor neurons and lower levels of SMA protein in patient-derived cells compared to controls. Drug screening using this model identified valproic acid (VPA) and tobramycin as potential treatments. However, VPA failed in clinical trials, and tobramycin still requires further toxicity evaluation before clinical application.^392,393^ Since then, iPSC-based disease modeling has expanded significantly to include a wide range of neurological disorders, such as Parkinson disease (PD),^394^ ALS,^395^ and multiple sclerosis.^396^ These models have also been instrumental in drug discovery efforts for these conditions.^397–399^ For example, a microfluidics-based platform has been developed to screen drugs using iPSC-derived motor neurons, allowing precise and controlled exposure to candidate compounds.^400^ This integration of iPSC technology with organ-on-a-chip systems has led to the development of advanced models for various tissues, including: brain,^401–403^ liver,^404^ retina,^405^ heart,^406–408^ neurons,^409^ and intestine.^410^ These platforms combine the physiological relevance of iPSC-derived cells with the precision of microfluidics, enhancing both drug screening and therapeutic development. Another early and influential use of iPSCs was in modeling cardiac long-QT syndrome (LQTS) type 1, a genetic disorder that causes ventricular arrhythmias and sudden cardiac death.^411^ iPSCs were generated from dermal fibroblasts of both healthy individuals and LQTS-affected family members using lentiviral vectors encoding OCT3/4, SOX2, KLF4, and c-MYC.^412^ The resulting cardiomyocytes exhibited distinct action potential profiles, and a specific KCNQ1 mutation was identified, which responded to *β*-blocker therapy. More recently, a dual KCNQ1/TRPM4 mutation model using iPSCs identified verapamil as a potential treatment.^413^ Comparative iPSC models for LQTS types 2 and 3 have also been developed, enabling tailored therapeutic strategies.^414,415^ Recent research has further enhanced the utility of iPSC-derived cardiomyocytes (iPSC-CMs) by improving their maturation through electrostimulation and small molecule treatments.^416,417^ These advances have expanded their application to modeling a range of cardiac conditions, including: hypertrophic cardiomyopathy,^418^ single-ventricle congenital defects,^419^ dilated cardiomyopathy,^420^ and proarrhythmic risk.^421^

Retinal diseases such as Vogt-Koyanagi-Harada (VKH) syndrome, oculocutaneous albinism (OCA), age-related macular degeneration (AMD), and various inherited retinal diseases (IRDs) are characterized by dysfunction of the retinal pigment epithelium (RPE)—a critical layer of cells that supports retinal health.^422–424^ These conditions can lead to progressive vision loss and, in severe cases, legal blindness. Currently, only 1 gene-replacement therapy is approved for IRDs, and it applies to fewer than 2% of patients, underscoring the urgent need for better disease models and drug screening platforms.^425^ To address this gap, ongoing efforts focus on iPSCs to develop patient-specific, biologically relevant RPE models for studying disease mechanisms and testing potential treatments.^426–429^ In a study on OCA, iPSCs were generated from peripheral blood mononuclear cells (PBMCs) of a patient with OCA type 1 using episomal plasmids expressing OCT4, SOX2, c-MYC, and KLF4.^430^ These iPSCs were then differentiated into RPE cells using an embryoid body—based protocol with SMAD and Wnt pathway inhibition, resulting in an OCA-specific RPE cell line.^427^ Similarly, in an AMD study, patient-derived iPSCs were created using Sendai virus transduction and differentiated into RPE cells. These cells were screened with a small library of therapeutic compounds, leading to the identification of 5-aminoimidazole-4-carboxamide ribonucleotide, synthetic analog of adenosine monophosphate and metformin as agents that improved mitochondrial function—an important factor in AMD pathology.^431,432^ For VKH syndrome, RPE dysfunction is considered an early indicator of this multiorgan autoimmune disease. Researchers generated iPSCs from PBMCs of a patient with VKH, using plasmids encoding OCT3/4, SOX2, KLF4, LIN28, and MYCL, and differentiated them into RPE cells that retained disease-specific genetic features.^428^ A genome-wide association study had previously identified EGR2 as a key gene associated with VKH.^433^ Using this insight, a high-throughput virtual screen of 1.39 million compounds was conducted, leading to the discovery of CQMU98, a compound that restored RPE barrier function in the VKH iPSC model—demonstrating the power of iPSC-based platforms for drug discovery. These advances are especially promising given the large number of incurable IRDs.^425^ iPSC-derived RPE models are now being used to study a wide range of retinal diseases, including retinitis pigmentosa,^434,435^ Leber congenital amaurosis,^436,437^ choroideremia,^438^ gyrate atrophy,^439^ Stargardt disease,^440^ and best vitelliform macular dystrophy.^441^ Together, these studies highlight the growing role of iPSC technology in advancing our understanding of retinal diseases and accelerating the development of personalized, effective therapies.

Traditional drug discovery methods that rely on animal models are often slow, expensive, and limited in their ability to predict human-specific responses (Fig. 6). In contrast, iPSC models offer a powerful alternative, enabling high-throughput drug screening with greater specificity and relevance to human biology. For example, an iPSC-derived cardiac organoid has been developed that closely mimics the pathology and gene expression profile of an infarcted human heart. This model was sensitive enough to detect the cardiotoxic effects of doxorubicin, a common chemotherapy drug.^442^ Similarly, human iPSC (hiPSC)—derived cardiomyocytes have been used to screen for cardiotoxicity of anticancer agents, including kinase inhibitors and trastuzumab, in vitro.^443^

A major innovation in the field is the development of chemically iPSCs (CiPSCs), which offer a safer and more efficient alternative to traditional reprogramming methods that use viral vectors. The first CiPSCs were generated in 2013 using a cocktail of 7 small molecules, achieving a reprogramming efficiency of 0.2% over 40 days—10 times higher than the original OSKM method.^54,444^ This approach has since been refined into a 4-stage protocol (fibroblast → stage I → epithelial-like → stage 2 → intermediate plastic state → stage 3 → embryonic endoderm—like → stage IV → iPSC) that guides cells through intermediate states using different chemical cocktails, reaching efficiencies of up to 2.56% in 50 days.^445^ More recently, a 3-stage protocol has further improved efficiency to 31% for adipose-derived stem cells and 4.27% for skin fibroblasts within 30 days.^446^ This method enhances safety and efficiency by avoiding viral integration.

Avoiding viral integration is critical, as inserting foreign genetic material into the host genome can lead to mutations, aberrant protein expression, oncogenesis, autoimmune reactions, or cell death.^447–449^ Chemical reprogramming enhances safety by eliminating these risks while offering greater control over reprogramming conditions. Small molecules used in CiPSC protocols can replace traditional Yamanaka factors. For instance: forskolin can substitute for OCT4, RepSox for SOX2, BMP4 for KLF4, and vitamin C for c-MYC.^444,450,451^

The effectiveness of CiPSCs has been validated through transcriptomic profiling and pluripotency marker expression.^452^ Although using multiple compounds can complicate the identification of specific cellular effects due to potential off-target actions, ongoing research is optimizing these protocols for greater precision and safety. One fully chemical-based cocktail has been shown to replace all OSKM factors in reprogramming mouse embryonic fibroblasts (MEFs). This cocktail includes the following:

- VPA—a histone deacetylase inhibitor
- CHIR99021—a GSK3 inhibitor
- RepSox—an ALK5 inhibitor
- Parnate—a lysine demethylase 1 inhibitor
- Forskolin—an adenylyl cyclase activator
- DZNep—a histone methyltransferase inhibitor
- TTNPB—a retinoic acid analog^444^

Further refinement using BrdU led to a minimal 4-chemical cocktail—BrdU, CHIR99021, RepSox, and forskolin—which was sufficient to generate stable iPSCs.^453^ Additional small molecule cocktails have been developed to reprogram cells from various sources: urine-derived cells,^454^ intestinal epithelial cells,^455^ MEFs,^456^ adipose-derived mesenchymal stromal cells, and human adult skin fibroblasts.^445^ These efforts often focus on enhancing the efficiency of genetic reprogramming or improving the maturation of differentiated cell products.

Small molecules used in reprogramming cocktails span a wide range of biochemical and pharmacological functions, including (Table 2)^457–536^: phytochemicals (eg, curcumin, salidroside, and forskolin), epigenetic modifiers (eg, VPA, 3-Deazaneplanocin A, inhibitor of both S-adenosylhomocysteine (AdoHcy) hydrolase and EZH2, a histone methyltransferase, and trichostatin A), kinase inhibitors (eg, RepSox, CHIR99021, and baricitinib), and kinase activators (eg, metformin, 8-Br-cAMP, and phorbol 12-myristate 13-acetate).^537^ These compounds vary in structure and function—for example, histone methylation modifiers often contain nitrile or halogen groups, while acetylation modifiers tend to feature ketone groups. The use of small molecule cocktails to generate iPSCs (Table 2) offers a versatile and scalable platform for disease modeling, drug screening, and personalized therapy development. These tools are helping researchers better understand disease mechanisms and identify patient-specific treatments with greater precision.

Advances in computational biology have significantly enhanced the utility of iPSCs in drug discovery. One key application is the use of in silico modeling to analyze the genetic profiles of iPSCs and their differentiated derivatives, helping to identify small molecules that can regulate cell fate and reprogramming.^538^ Progress in this area has been accelerated by integrating multiple technologies. For example, researchers have combined nanodot array platforms—which generate measurable nanotopographies during iPSC differentiation—with gene expression profiling and in silico connectivity map analysis to identify compounds that influence cell behavior.^539^ In another study, iPSC-CMs were paired with computational simulations of ligand—membrane interactions to identify patient-specific therapeutic compounds, focusing on optimizing safety, efficacy, and potency.^540^ The integration of artificial intelligence (AI) and machine learning (ML) is further transforming in silico drug discovery. These tools enable high-throughput screening of combinatorial chemical libraries to identify compounds capable of inducing cellular reprogramming.^541^ AI and ML can also predict synergistic effects between compounds, reducing the time and cost associated with traditional in vitro and animal model testing. Beyond screening, AI and ML are being used to analyze large-scale biological datasets to predict drug safety and efficacy.^542^ For instance, knowledge graphs and ML algorithms can define meta-paths that link molecular targets, signaling pathways, and tissue-specific disease mechanisms—providing early insights into potential therapeutic strategies.^543^ AI is also playing a growing role in drug design, particularly in predicting pharmacodynamic properties that influence the safety and toxicity of gene therapies, antibodies, oligonucleotides, and vaccines.^543^ More recently, generative pretrained transformer models—originally developed for natural language processing—are being adapted for biomedical research. These include the following:

- scGPT (foundation model designed to integrate and analyze large-scale single-cell multi-omics data using a generative pre-trained transformer [GPT] architecture) for analyzing multiomics and single-cell data,
- scBERT (single-cell Bidirectional Encoder Representations from Transformers, a pre-trained deep neural network-based model) for modeling cell type—specific gene expression dynamics, and
- Geneformer for predictive modeling in network biology.^544–547^

These AI-driven tools are accelerating the pace of disease modeling and drug discovery, offering new ways to interpret complex biological systems and identify personalized therapeutic solutions.

iPSC models have become powerful tools for studying diseases that currently lack effective treatments. They have significantly advanced our understanding of disease mechanisms and supported the discovery of new therapies. Recent successes include modeling a wide range of conditions such as familial hypercholesterolemia liver disease,^548,549^ Alagille syndrome-type 1,^550^ *α*1 antitrypsin deficiency,^551^ hemophilia B,^552^ nonalcoholic fatty liver disease,^553^ Duchenne muscular dystrophy (DMD),^554^ cystic fibrosis,^555^ and cancer immunotherapy models.^556,557^ These models have also facilitated drug discovery efforts for conditions such as liver fibrosis,^558^ nonalcoholic fatty liver disease,^559^ progressive familial intrahepatic cholestasis type 2,^560^ PD,^397^ AD (ClinicalTrials.gov ID NCT04413344),^399^ biliary disease,^561^ severe acute respiratory syndrome coronavirus 2,^562^ dilated cardiomyopathy,^420^ ALS (ClinicalTrials.gov ID NCT02450552; UMIN-CRT clinical trials UMIN000034954 and UMIN000036295), and frontotemporal dementia (ClinicalTrials.gov ID NCT04931862).

To improve the physiological relevance of iPSC-based models, researchers have developed 3D organoid culture systems. These systems better replicate the cellular microenvironment, tissue architecture, and function of human organs compared with traditional 2D cultures. Organoids fall into 2 main categories:

- Scaffold-free systems—self-assembled spheroids or cell aggregates;
- Scaffold-based systems—use of solid or liquid biomaterials to support and guide cell organization.^563,564^

Organoids have been created from various tissues, including the brain,^565^ liver,^463^ lung,^566^ reproductive system,^567^ skin,^568^ heart,^569^ kidney,^570^ and bile duct.^571^ Despite the advantages of 3D systems, many researchers still use 2D cultures for their simplicity and scalability—especially when the cells exhibit disease-relevant phenotypes.^572^

Scaffold-based 3D cultures offer an additional layer of complexity by enhancing cell—cell and cell—matrix interactions. These systems often use hydrogels made from ECM components like collagen and fibrin, or other biocompatible materials, to create a supportive 3D environment.^573,574^ iPSC-derived cells benefit from ECM-rich hydrogels—especially those derived from decellularized target tissues—which promote cell differentiation and maturation, helping the cells more closely resemble their in vivo counterparts in both structure and gene expression.^575^ The functional maturity of iPSC-derived cells can be further enhanced by coculturing them with other cell types naturally found in the target tissue. This approach improves the physiological relevance of the model and supports more accurate disease modeling and drug testing.^576^

The properties of biocompatible hydrogels—such as absorption, flexibility, and stability—are influenced by factors like the type of polymer used, the degree of crosslinking, the presence of functional groups, and their hydrophilic nature.^577,578^ Cell adhesion and cellular interactions can be further modulated by incorporating specific peptides, such as arginyl-glycyl-aspartic acid, and by adjusting polymer side groups, pH levels, and electrostatic charges.^578,579^ Additionally, hydrogels can be engineered to respond to external stimuli—including mechanical stress, ultrasound, ions, light, temperature, magnetic fields, electric currents, and biological signals—to release embedded agents like drugs. These stimuli can trigger changes in the hydrogel’s swelling, degradation, or release behavior, enabling precise control over the local microenvironment. The integration of 3D cell culture with 3D printing technologies has further advanced the field of bioprinting.

Bioprinting is a technique that uses bioinks—combinations of cells and hydrogels or other biomaterials—to fabricate 3D structures that closely mimic natural tissues.^580^ A compelling example is the bioprinted model of post-MI scarring, where iPSC-CMs and cardiac fibroblasts were embedded in a self-healing hydrogel to replicate damaged heart tissue. This model enables therapeutic screening in a physiologically relevant context.^581^ Bioprinting has been successfully applied to generate a wide range of iPSC-derived tissues, including kidney,^582^ heart,^583^ liver,^584,585^ nerve,^586,587^ cornea,^588^ and muscle.^589^ Additionally, undifferentiated hiPSCs have been printed into scaffolds designed to induce in situ differentiation.^590^ Despite its promise, scaffold-based bioprinting faces several challenges. Trade-offs between printing speed, cell expansion time, cost, cell viability, and anatomical accuracy limit its clinical scalability. Moreover, the scaffold materials themselves can interfere with drug screening by absorbing compounds, potentially skewing results. For instance, hepatocyte spheroids were tethered to a galactose-conjugated hydrogel “soft sponge” to reduce drug absorption.^591^ Other limitations include batch-to-batch variability in hydrogel production and the risk of xenogeneic contamination from animal-derived materials like Matrigel (Corning Life Sciences), which can affect reproducibility and immune response studies.^592,593^ Additional hurdles include maintaining scaffold integrity, controlling degradation rates, and scaling up iPSC production in 2D cultures for 3D applications.

To overcome these barriers, researchers are exploring scaffold-free 3D culture systems. Meanwhile, recent innovations continue to enhance scaffold-based approaches. These include density gradient hydrogels that improve cell infiltration and survival,^577^ hydrogels with varied pore sizes that support better cell adhesion and proliferation,^594^ and the integration of ML to optimize printing precision and biomimicry.^595^ Advanced scaffolds, such as laser-microstructured matrices seeded with hiPSC-derived NSCs, have enabled the growth and differentiation of diverse neuron types, deepening our understanding of neural networks.^596^ Furthermore, combining scaffolds with hiPSC-derived vascular organoids has led to the creation of hierarchical vascular tissues, offering promising platforms for drug screening and physiological research.^597^

In scaffold-free 3D cultures, cells are suspended in a gently rotating liquid medium, allowing them to naturally aggregate and self-assemble into spheroids. This contrasts with the flat, monolayer format of 2D cultures and the structured environments of scaffold-based systems.^583^ These spheroids can produce their own ECM, creating a microenvironment that more closely mimics in vivo conditions. Early studies using 3D cultures, such as the one^598^ that examined 25 breast cancer cell lines, identified distinct spheroid morphologies—round, mass, grape-like, and stellate—each reflecting different levels of organization, cell—cell communication, and invasive behavior. Transitioning from 2D to 3D culture significantly alters gene expression profiles, making 3D models more representative of physiological states and better suited for studying disease progression and drug responses.^598,599^ A notable example is the first scaffold-free 3D iPSC model of AD, which used patient-derived neurospheroids to compare 2D and 3D culture conditions. While 2D cultures showed reduced amyloid *β* peptide synthesis in response to *β*-secretase inhibitors, the 3D cultures exhibited a diminished drug response—highlighting the superior physiological relevance of 3D models.^600^

Recent advances have further demonstrated the potential of scaffold-free systems. For instance, human iPSC-CMs have been used to create 3D cardiac microtissues that beat spontaneously, maintain viability for >100 days, and exhibit transcriptional and metabolic profiles similar to native heart tissue.^601^ Techniques like the Kenzan method use soft bio-3D printers to arrange spheroids on needle arrays, enabling faster and gentler assembly of complex 3D structures compared to scaffold-based approaches.^583,602,603^ This method has been applied across various fields, including nerve and cartilage regeneration, glioma invasion modeling, and liver toxicity testing.^604–608^ Despite these advances, challenges remain—particularly in integrating multiple systems such as vascular, immune, and nervous components to fully replicate the complexity of human tissues. Nevertheless, both scaffold-based and scaffold-free 3D culture systems continue to evolve, pushing the boundaries of disease modeling, drug screening, and the development of patient-specific organ models with the potential to reduce immune rejection in transplantation.^609–611^

In recent years, iPSC-based models of the BBB have been extensively developed using both 2D and 3D culture systems to better understand the mechanisms underlying neurodegenerative diseases. 2D BBB models typically involve culturing cells in transwell assay systems, which allow researchers to study the cellular components and molecular mechanisms that regulate barrier permeability.^612–615^ In contrast, 3D BBB models leverage more physiologically relevant microenvironments created through hydrogels, microfluidic devices, and brain organoids, eliminating the need for transwell setups.^616–618^ These 3D systems more accurately replicate the structural and functional complexity of the BBB. Notably, brain organoid-based models derived from iPSCs can be generated in approximately 2 weeks and maintained in long-term culture. This makes them particularly valuable for studying BBB permeability, cellular interactions, and for conducting drug screening assays.^619^ The evolution from 2D to 3D iPSC-based BBB models marks a significant advancement in neuroscience research. While 2D systems remain useful for mechanistic studies, 3D models offer a more realistic and dynamic platform for investigating disease progression and therapeutic responses, paving the way for more predictive and translational research in neurodegenerative disorders.

### B. Direct reprogramming

Direct reprogramming, formerly discussed as transdifferentiation, is a technique that converts one mature cell type directly into another without passing through a pluripotent or progenitor state. This approach bypasses the need for iPSCs and has gained traction for its potential in disease modeling, drug screening, and preclinical testing of regenerative therapies.^4^ Fibroblasts—particularly those derived from skin—are among the most commonly used starting cells for direct reprogramming due to their abundance across tissues and their demonstrated plasticity. These cells have been successfully reprogrammed into a wide variety of functional cell types: cardiomyocytes,^478,620^ neurons,^621^ osteoblasts,^622^ chondrocytes,^623^ hepatocytes,^624^ smooth muscle cells,^625^ skeletal muscle cells,^524^ insulin-producing *β* cells,^626^ and adipocytes.^530^ This versatility highlights the potential of fibroblasts in regenerative medicine. However, direct reprogramming is not limited to fibroblasts; the broader principle of cellular plasticity suggests that many cell types could be reprogrammed under the right conditions.^627,628^ Recent strategies and outcomes of direct reprogramming are summarized in Table 3.^629–682^ For example, in a study focused on primary hyperoxaluria type 1, patient-derived skin fibroblasts were directly reprogrammed into induced hepatocytes (iHeps) using lentiviral transduction of transcription factors FOXA2, HNF1a, HNF4a, and TBX3.^675^ These iHeps were then genetically corrected using CRISPR-Cas9 to knock in the *AGXT* gene, which reduced oxalate accumulation. This approach offers a promising therapeutic strategy for treating primary hyperoxaluria type 1 through liver cell replacement therapy.^675,683^

In another study, mouse adipocytes isolated from skin were reprogrammed to assess their potential as an alternative source of dermal fibroblasts for wound healing. Using a retroviral vector, researchers introduced transcription factors ELF4, FOXC2, FOXO1, IRF1, PRRX1, and ZEB1.^654^ This combination successfully converted adipocytes into fibroblast-like cells, as evidenced by decreased expression of adipocyte markers (C/EBP, aP2, and leptin) and increased expression of the fibroblast-associated marker anti-ER-TR7. This transdifferentiation process, which also occurs in certain cancer pathologies,^684^ may reflect the inherent plasticity of adipocytes. The model presents a potential therapeutic strategy for promoting skin regeneration in cases where grafting is not feasible, such as extensive burn injuries. In summary, direct reprogramming offers a powerful and flexible platform for generating patient-specific cell types for research and therapeutic use. By bypassing the pluripotent state, it reduces the risk of tumorigenicity and accelerates the production of functional cells. As techniques continue to evolve, direct reprogramming holds significant promise for personalized medicine and regenerative therapies.

Retinal degeneration and vision loss are devastating consequences of diseases such as macular degeneration, glaucoma, and diabetic retinopathy. In mammals, the loss of retinal ganglion cells (RGCs) is particularly problematic, as these neurons do not naturally regenerate once damaged. Early breakthroughs in direct reprogramming provided a foundation for addressing this challenge. In a landmark study, researchers demonstrated that the pioneer transcription factor Ascl1, when combined with Brn2 and Myt1l—collectively known as the Ascl1, Brn2, and Myt1l (ABM) cocktail—could reprogram MEFs into induced neurons (iNs).^685^ These iNs exhibited key neuronal features, including action potentials, synapse formation, and contractility, making them valuable tools for modeling neurological diseases and screening potential therapeutics. Building on this approach, another research group modified the ABM cocktail by replacing Myt1l with Isl1, a LIM homeodomain transcription factor essential for RGC development and maturation. This revised combination—Ascl1, Brn2, and Isl1—successfully directed MEFs to differentiate into RGC-like neurons, effectively bypassing non-RGC fates.^686^ The resulting cells not only formed functional synapses but also displayed transcriptomic profiles closely resembling those of native RGCs. This enhanced specificity in reprogramming leads to a more homogeneous population of RGC-like neurons, minimizing the presence of off-target cell types that could otherwise confound drug screening results. As a result, this refined method offers a promising platform for studying retinal diseases and developing targeted therapies.

In a study focused on neurodevelopmental disorders, researchers developed an in vitro model of Rett Syndrome using dermal fibroblasts from patients with X-linked MECP2 mutations, which are known to impair brain development.^674^ These fibroblasts were directly reprogrammed into iNs through transient episomal plasmid expression of the transcription factors SOX2 and PAX6. The resulting iNs successfully recapitulated key features of Rett syndrome pathology, as confirmed by RNA sequencing, histone acetylation patterns, and dendritic arborization analysis. This work highlights the power of direct reprogramming to generate disease-relevant neuronal models, especially for conditions like Rett syndrome where brain tissue samples are difficult to obtain. Such models are invaluable for studying disease mechanisms and testing potential therapies for severe neurological disorders. Direct reprogramming is also gaining traction as a promising strategy for addressing neurodegenerative diseases and brain injuries, which are traditionally difficult to treat. Recent studies have explored various ex vivo astrocyte-to-neuron conversion techniques. For example, midbrain astrocytes have been directly reprogrammed into dopaminergic iNs using retroviral overexpression of transcription factors such as NURR1, SHH, BCLxL, ASCL1, and a stabilized variant, ASCL1-5SA.^670^ Similarly, cortical astrocytes have been converted into GABAergic or glutamatergic/dopaminergic iNs using retroviral CEND1 (cell cycle exit and neuronal differentiation [NeuroD]1) or Neurog2, in combination with small molecules like forskolin and VPA.^500^ In addition to transcription factor-based methods, chemical reprogramming has emerged as a safer alternative for potential therapeutic applications. One study demonstrated the conversion of astrocytes into neurons using a cocktail of small molecules—including Y26732, DAPT, RepSox, CHIR99021, ruxolitinib, and smoothened agonist (SAG)—in the induction media.^521^ Compared with genetic approaches, small molecule—based reprogramming is generally considered safer for future transplantation due to reduced risks of genomic integration.^687^

Combining small molecules with transcription factors has proven to be an effective strategy in direct reprogramming, significantly enhancing the efficiency and precision of cell fate conversions. For example, after traumatic brain injury, brain-resident microglia—including macrophages—migrate to the site of damage. Researchers developed an in vitro model to directly reprogram microglia into iNs using the transcription factor NeuroD1.^688^ When mouse brain microglia were transduced with a NeuroD1-expressing lentiviral vector, a portion of the cells successfully converted into iNs. The reprogramming efficiency more than doubled—from less than 20% to more than 50%—when NeuroD1 was combined with a small molecule cocktail of VPA, forskolin, and Y-27632.^532^ This study, validated through lineage tracing, was the first to demonstrate direct conversion of microglia (rather than meningeal macrophages) into neurons, underscoring the enhanced efficacy of incorporating small molecules into reprogramming protocols. Fibroblast-to-neuron reprogramming has also benefited from the inclusion of small molecules. Earlier studies showed that fibroblasts could be converted into iNs using the ABM cocktail (ASCL1, BRN2, and MYT1L),^685^ and that ASCL1 alone was sufficient to generate excitatory iNs.^689^ To reduce the risks associated with viral integration and tumorigenicity, researchers developed a nonviral system using a fusion protein of 30Kc19-Ascl1—a cell-penetrating peptide derived from silkworm hemolymph—combined with VPA. This approach successfully reprogrammed mouse fibroblasts into iNs in vitro.^690^ In a similar effort to avoid viral vectors, another study used episomal plasmids to reprogram human fibroblasts.^649^ These plasmids carried ASCL1, neuron-specific miR-124, and p53 short-hairpin RNA and were transfected into human foreskin fibroblasts (HFFs). By 21 days after transfection, the cells exhibited neuron-specific gene expression, achieving direct conversion without passing through a pluripotent stem cell state. This nonintegrating method offers a safer and more clinically relevant platform for modeling neurological diseases.

Direct reprogramming has also been applied to generate induced cardiomyocytes (iCMs) from human dermal fibroblasts (HDF) for in vitro drug screening and tissue engineering.^468^ In this study, fibroblasts were transduced with lentiviral vectors expressing GATA4, MEF2C, TBX5, MYOCD, and NKX2-5 and reverse transfected with miR-1 and miR-133a. This combination suppressed fibroblast markers and activated cardiac-specific genes, as evidenced by intracellular calcium transients. Although the addition of small molecules—such as a JAK1 inhibitor (EMD4Biosciences; 420099) and the GSK3 inhibitor CHIR99021—improved reprogramming efficiency, it remained <25%. Moreover, the resulting iCM-like cells did not exhibit full contractile function, indicating that further optimization is needed to produce fully functional cardiomyocytes for therapeutic applications.

Recent advances in direct reprogramming have shown that combining small molecules with transcription factors significantly improves the generation of functional iCMs. In one study, researchers screened 4960 transcription factors using an automated high-throughput platform and identified a novel combination—MYOCD, SMAD6, and TBX20 (MST)—capable of reprogramming primary human cardiac fibroblasts into iCMs.^536^ When this MST cocktail was combined with the growth factor FGF2 and the small molecule XAV939 (a WNT pathway inhibitor), the resulting iCMs exhibited spontaneous contractions and calcium transients—key indicators of functional cardiomyocytes. Hierarchical clustering analysis further revealed that MST-induced iCMs more closely resembled hiPSC-derived cardiomyocytes than those generated using the earlier GMT method (GATA4, MEF2C, and TBX5).^691^ In another study, a chemical screen of 2000 compounds identified SB431542 (a TGF-*β* inhibitor) and baricitinib (a Janus kinase inhibitor) as enhancers of cardiac fibroblast reprogramming when used alongside the transcription factors MEF2C and TBX5 (MT).^472^ This combination significantly improved reprogramming efficiency and promoted spontaneous contractile activity in the resulting iCMs. Together, these MST and MT-based reprogramming strategies—augmented by small molecules—offer robust platforms for generating functional iCMs. These in vitro models hold great promise for disease modeling, drug screening, and potentially, future regenerative therapies.^472,536^

Generating functional iNs for human neurodegenerative disease modeling and drug discovery is a major focus within the field of direct reprogramming. The optimization of transcription factor combinations and small molecule cocktails for reprogramming has been continuously evolving since the inception of direct reprogramming techniques. For example, skin fibroblasts from patients with ALS have been successfully transdifferentiated into human-induced motor neurons using a lentiviral vector to deliver the transcription factors NGN2, ISL1, SOX11, and LHX3, along with a T2A (ribosomal-skipping) peptide.^508^ This method reportedly transdifferentiated >84% of ALS fibroblasts into cervical and thoracic spinal motor neurons. Using this human induced motor neuron model, a chemical screen identified the small molecule kenpaullone as a potential therapeutic compound capable of rescuing ALS-specific disease characteristics, including restoring electrophysiological activity and the ability to form NMJs.^508^ To avoid viral transduction, researchers have also developed small molecule-only cocktails capable of converting somatic cells into iNs. For example, HFFs were rapidly transdifferentiated into neuron-like cells (eg, TUJ1^+^ and DXC^+^) with more than 70% efficiency within 2 days using a small molecule cocktail that included CHIR99021, RepSox, forskolin, Y-27632, JQ-1^+^, and trichostatin A.^507^ Additionally, HFFs have been transdifferentiated into electrophysiologically functional iNs using a different small molecule cocktail (forskolin, RepSox, SP600125, CHIR99021, Go6983, Y-27632, IXS9, and I BET151) over a 30-day period, achieving a conversion efficiency of >80%.^527^

A key objective in developing iNs for disease modeling and drug screening is to obtain reprogrammable cells from noninvasive sources. This has led researchers to explore sources such as urine, using small molecule, nonviral reprogramming techniques to avoid potential genomic integration. Direct reprogramming of urine cells has been attempted with various small molecule combinations, such as CHIR99021, A8301, Y27632, TTNPB, forskolin, VPA, and NaB (CAYTFVB),^510^ as well as CHIR99021, RepSox, VPA, Parnate, TTNPB, and Dznep (CRVPTD).^495^ These approaches used CHIR99021 (a GSK3 inhibitor) and TTNPB (a retinoic acid receptor activator) to activate the Wnt, retinoic acid receptor, and cAMP-dependent pathways, while VPA was used to promote mTOR signaling.^692^ Although both cocktails successfully reprogrammed urine cells into neuron-like cells that expressed neuron-specific gene markers and exhibited neuronal morphologies, the resulting cells demonstrated minimal electrophysiological activity and were unable to produce calcium action potentials.^495,510^ While these neuron-like cells are unsuitable for cell replacement therapies due to their lack of action potential functionality, they still offer valuable tools for noninvasive, patient-specific disease modeling and therapeutic drug screening.

In an effort to develop an AD model for mechanistic studies and drug discovery, researchers combined small molecule reprogramming factors with microfluidics technology to transdifferentiate skin fibroblasts from patients with familial AD (FAD) into iNs and create a “lab-on-chip” high-throughput assay platform.^462^ The chemical cocktails used for this purpose included CHIR99021, LDN193189, RG108, dorsomorphin, P7C3-A20, A83-01, and ISX9 (CA-cocktail), as well as forskolin, Y27632, DAPT, PD0325901, A83-01, purmorphamine, and P7C3-A20 (CB-cocktail). The CA-cocktail was applied to FADs via channels on a microfluidics device for 3 days, followed by the CB-cocktail for 9 days, successfully generating mature iNs with an impressive 61% efficiency—more than double the efficiency achieved when FADs were transdifferentiated with a similar small molecule cocktail (VPA, RepSox, forskolin, SP600125, Go6983, and Y-27632) in traditional 24-well plates. The resulting FAD-iNs generated on the microfluidics chip retained neurodegenerative phenotypic abnormalities associated with FAD. This innovative approach of chemically reprogramming FADs into functional FAD-iNs on a microfluidics device provides a noninvasive, patient-specific high-throughput platform for disease modeling and drug screening without the need for viral constructs or iPSC intermediates.^462^

Extracellular vesicles (EVs) are small lipid-enclosed compartments naturally released by all cell types. Extensively characterized by the International Society for Extracellular Vesicles, EVs function as mediators of cell-to-cell communication, transmitting information through proteins, nucleic acids, and metabolites.^693–696^ Researchers have begun leveraging the utility and permeability of EVs and exosomes to deliver reprogramming cargo nonvirally for the transdifferentiation of target cells. For example, in the case of aortic valve disease, where calcification can reduce blood flow and eventually lead to heart failure,^697^ a research group has developed a method using EVs to directly reprogram calcified valve cells into macrophages. They loaded EVs derived from HDFs with plasmid DNA and mRNA encoding the transcription factors CEBPA and Spi1I. These EVs were then successfully delivered to ex vivo calcified aortic valve tissue, reprogramming human endothelial cells into anti-inflammatory macrophage-like cells. This EV-based reprogramming presents a promising therapeutic strategy for patients ineligible for valve replacement surgery.^671^ Given that exosomes are a subset of EVs with similar functions, it is not surprising that they are also being explored for cell delivery strategies in disease modeling. For instance, fibrosis complicates both hepatitis C viral (HCV) liver disease and lung silicosis.^698,699^ In HCV liver disease modeling, miR-192 was identified as a regulator of TGF-*β*1, which influences the transdifferentiation of hepatic stellate cells into myofibroblasts, contributing to liver fibrosis.^650^ Exosomes loaded with miR-192 induced this fibrogenic state in hepatic stellate cells, while those containing anti-miR-192 reversed the process. Similarly, in lung silicosis modeling, macrophages exposed to silica were used to phagocytose particles, after which exosomes were isolated and cultured with primary lung fibroblasts.^651^ This led to the transdifferentiation of fibroblasts into myofibroblasts, with miR-125-5p and its target Smurf1 identified as key regulators of this transition via TGF-*β* signaling. Further, previous data had identified SPP1 as a silica-exposed macrophage exosomal protein involved in the regulation of fibroblast to myofibroblast transdifferentiation.^661^ These findings significantly advance our understanding of the mechanisms underlying fibrotic lung disease in silicosis. Overall, the use of EVs and exosomes as naturally occurring, nonviral delivery system presents a promising avenue for direct reprogramming strategies, as demonstrated in aortic valve disease and HCV liver disease, and for disease modeling in conditions such as lung silicosis.

As discussed in the context of iPSC methodology for replicating organ disease states in culture, 3D organoid cultures offer a more physiologically relevant model of organ function than 2D cultures. Organoids benefit from their 3D structure, which allows for enhanced cell—cell interactions and microenvironmental influences, more closely mimicking in vivo conditions. Using 3D organoid culture systems in combination with direct reprogramming techniques provides robust models for studying disease progression and for drug screening.

For example, primary human hepatocytes have been directly reprogrammed using a combination of 3 small molecules (A83-01, CHIR99021, and HGF) to create chemically derived hepatic progenitor cells for liver organoid production.^463,464^ Instead of using Matrigel, which can contain unknown proteins and exhibit batch variability,^593^ a collagen scaffold was used to generate an organoid product suitable for potential clinical applications. The use of chemically derived hepatic progenitor cells derived from small molecules to generate organoids resulted in a 10-fold increase in reprogramming efficiency, a 68% increase in growth rate, and improved expression of hepatic progenitor markers compared with organoids derived from primary hepatocytes.^463^ This method enabled the development of liver organoids without the need for iPSC intermediates or viral intervention, making them suitable for disease modeling, drug screening, and potentially cell replacement therapy, given the use of a collagen scaffold instead of Matrigel. Similarly, another research group recently used small molecule direct reprogramming to produce intestinal 3D organoids from human urine cells (hUCs).^469^ This process involved 3 steps: first, transforming hUCs into hUC-induced endoderm progenitor cells in 2D culture using small molecules such as CHIR99021, EPZ5676, B27, *β*-ME, RG108, P8511, activin A, LYZ294002, and EPZ011989. Next, induced endoderm progenitor cells were differentiated into midgut/hindgut cells using Noggin, SB431542, CHIR99021, and SAG. Finally, these midgut/hindgut cells were induced to become intestinal progenitor cells with CHIR99021, BMP4, and RA before being embedded in Matrigel to form hUC—derived induced intestinal organoids (U-iIOs). These direct reprogramming steps were carefully designed to guide cells with multiple differentiation potentials toward the goal of forming intestinal progenitor cells. U-iIOs created through direct small molecule reprogramming demonstrated active barrier function and genetic profiles more similar to the primary small intestine than those derived from iPSC indirect methods.^469^ These U-iIOs provide a valuable intestinal model for developing patient-specific treatments for various diseases.

In addition to the use of transcription factors and small molecules for direct reprogramming, a recent study demonstrated a novel approach using electrical stimulation alone.^648^ Electrical stimulation has been previously used to enhance the direct reprogramming of fibroblasts into neurons^320^ and to improve the reprogramming of iPSCs into cardiomyocytes.^700^ Building on the observation that MSCs can be aggregated and induced to differentiate into chondrocytes through electrical stimulation,^701^ researchers attempted a similar approach to induce chondrocytes from HDFs. HDFs were subjected to electrical stimulation at 5 V/cm and 10 Hz using a C-Pace EP culture pacer over a specific time course. Notably, the fibroblasts did not aggregate compactly under 6 or 10-miliseond stimulation; however, at 8 milliseconds of electrical stimulation, they formed compact aggregates and differentiated into chondrocyte-like cells. These induced chondrocyte-like cells exhibited increased expression of chondrocyte markers and a corresponding decrease in fibroblast markers. Additionally, they were karyotypically stable and did not carry the risk of tumor formation associated with the transduced genes used in other reprogramming methods.^702,703^ This makes electrical stimulation a promising technique for direct cell reprogramming ex vivo for clinical transplantation and potential in vivo applications.

Both indirect and direct reprogramming methods have unique advantages and limitations when developing models for disease study. Disease modeling using iPSCs can be particularly challenging in cases of polygenic diseases, where pinpointing the underlying causes may require a large number of patient and familial control samples to establish multiple iPSC lines, distinguishing causative factors from the background noise of epigenetic differences and natural genetic variation.^704^ Another significant challenge in disease modeling with iPSCs is the loss of age-associated phenotypes.^705^ Neurodegenerative diseases, such as AD, PD, and ALS, typically manifest in the aging immune system.^706,707^ Without the microenvironment of aging immune cells, in vitro models may fail to accurately recapitulate these diseases, thereby hindering meaningful conclusions. For example, iPSC-derived iNs for modeling AD generally have their age-related epigenetic information reset while retaining cell type—specific epigenetic markers.^708^ In contrast, iNs directly reprogrammed from patient fibroblasts retain age-related epigenetic information, making them more suitable for age-equivalent disease models, although they may lose some epigenetic information related to cell type developmental history.^709^ Efforts have been made to develop iPSC derivatives that retain aging-related phenotypes in vitro to better mimic the progression of late-stage neurodegenerative diseases. For instance, synthetic mRNA overexpression of progerin in PD-iPSC-derived dopamine neurons induced characteristics of late-stage PD, including dendrite degeneration, reduced tyrosine-hydroxylase expression, increased neuromelanin, and mitochondrial swelling, among others.^708^ Similarly, a hiPSC-derived neuronal/glial mixed-cell model was treated with rotenone to induce mitochondrial dysfunction and mimic PD aging-associated phenotypes.^710^ However, it remains debated whether these models adequately represent true aging or merely reflect cellular stress and oxidative damage.^711^

To address the challenge of erasing age-related phenotypes, direct reprogramming strategies can be used. Directly reprogramming fibroblasts into iNs does not involve passing through an iPSC intermediate state, which typically strips cells of their epigenetic aging-related phenotypes and leaves them in an embryonic-like state.^712^ Instead, direct reprogramming, such as converting fibroblasts into iNs, preserves epigenetic age-related gene expression profiles and aging phenotypes, resulting in iNs that more accurately model neurodegenerative disease states.

In silico bioinformatics analysis has also been used in predictive modeling of direct reprogramming. For example, use of a network biology platform, CellNet, is able to quantify the similarities in direct reprogrammed iCMs to the endogenous cardiomyocyte. Interestingly, CellNet has shown that iCMs resulting from direct reprogramming (via *Gata4*, *Mef2c*, and *Tbx5*) were less alike to endogenous CMs than iCMs that resulted from iPSC-mediated indirect reprogramming.^713^ This bioinformatics analysis approach showed the necessity for interrogating cellular reprogramming products and identified areas for improvement for direct reprogramming approaches. Combining computational modeling (Capybara) and ML (scREMOTE) programs with single-cell data is able to further our understanding of direct reprogramming approaches by identifying discrete intermediate states of cells transitioning into induced target cell products; identifying molecular signaling pathways that can be targeted to improve reprogramming yields; and predicting regulatory potential of reprogramming factors.^713–715^

## VI. Indirect reprogramming for cell-based and tissue-based therapies

Indirect reprogramming has been valuable for creating in vitro disease models and screening patient-specific therapeutic drugs. An important advancement in this field is the use of indirect reprogramming to generate viable cells for transplantation back into the host patient as part of cell replacement therapies.

### A. Significance of indirect reprogramming

The introduction of reprogramming factors by Yamanaka and Thomson has fundamentally transformed approaches to patient-specific cell-based therapies. A key advantage of this method is that it can circumvent the need for invasive or risky procedures to harvest cells from tissues such as the liver or CNS.^716,717^ Another significant benefit is the use of patient-specific cells to derive other cell types, which greatly reduces the potential for immune rejection following transplantation.^718^ Many laboratories are currently exploring the reprogramming of abundant differentiated adult skin fibroblasts into target cell types for therapeutic purposes.^719–726^ In 2016, the FDA implemented the 21st Century Cures Act to provide expedited pathways for advancing regenerative medicine therapy products, especially those targeting severe and life-threatening conditions with unmet medical needs.^727^ This legislation aims to promote the development of innovative cell-based products that have a favorable benefit-risk profile and straightforward manufacturing processes, thereby facilitating their progression to clinical trials.

iPSC methods are continually evolving as researchers strive to optimize transfection efficiency and proliferation rates while ensuring patient safety and minimizing transplantation risks. Common iPSC derivation methods include both viral (eg, Sendai virus, adenovirus, and adeno-associated virus [AAV]) and nonviral (eg, mRNA transfection, miRNA transfection, transposons, episomal plasmids, minicircle vectors, liposomal magnetofection, overexpression of protein reprogramming factors, and chemical induction) approaches.^728–730^ Notably, the source cells used for deriving iPSCs can also impart an epigenetic memory, which researchers must consider.^729,731^ This is evident in differences in DNA methylation between iPSC-derived and ESC-derived cells.^731^ iPSCs have been derived from a variety of sources, including skin fibroblasts, peripheral blood, hair follicle keratinocytes, urine renal cells, and biological waste materials.^471,730,732–738,^

### B. Current applications of indirect reprogramming

#### 1. Human pluripotent stem cell for therapeutic use

Currently, commercial products are making significant strides in using differentiated human pluripotent stem cells (hPSCs), whether derived from ESCs or iPSCs, for therapeutic treatments of various diseases. In the case of type 1 diabetes (T1D), differentiated hPSC therapies are being explored to provide patients with new *β* cells, potentially preventing or reversing T1D.^739^ Stem cell—derived islet replacement therapies have garnered attention due to the limitations of previous grafting methods, which required multiple donor pancreases (2–4) to provide sufficient donor islets.^70,740^ One of the aims of stem cell—derived islet therapy is to reduce or prevent immune rejection by eliminating the reliance on donors with diverse genetic backgrounds. Protocols for generating hPSC-derived islets involve a stepwise differentiation process to produce islet-like clusters, ensuring the elimination of progenitor or off-target cell types.^741^ This process sequentially treats hPSCs with specific small molecules and growth factors to mimic islet development through embryogenesis (hPSC → definitive endoderm → primitive gut tube → pancreatic progenitor → endocrine progenitor → stem cell—derived islet).^741^

For instance, an encapsulation device has been developed containing hESC-derived PEC-1 (islet progenitor) cells, designed to mature into fully functional *β* cells aftrr implantation in patients.^739,741^ A phase 1/2 clinical trial (NCT03163511) using this device demonstrated a 20% reduction in exogenous insulin dose for patients up to a year after implantation, along with meal-stimulated C-peptide secretion Despite these promising results, C-peptide secretion only reached 1% of normal levels and did not lead to insulin independence, possibly due to a low proportion of *β*/*α* cells in the explants.^742^ Notably, the device’s porous design allowed for host-derived vascularization, which supported sustained endocrine function.^741^ Another phase 1/2 clinical trial involved a fully differentiated stem cell—derived pancreatic islet replacement therapy (VX-880). In a single patient who received half the target dose of VX-880, there was a marked increase in C-peptide levels and improved glycemic control, with a 90% reduction in the need for exogenous insulin.^743^ The ongoing follow-up trial (NCT04786262) has reported similar preliminary results in the first 2 patients. While these studies are promising, several areas still require improvement, including enhancing the differentiation to non-*β* cells, improving the functionality of *β* cells, eliminating the need for immunosuppressants, and minimizing graft loss.^741^

#### 2. Induced pluripotent stem cells for therapeutic use

Despite the challenges, researchers are making significant progress in developing iPSC-derived cells for clinical therapies, using a variety of methods tailored to different target tissues. In 2014, Kamao et al^744^ conducted the first clinical trial using iPSC-based therapy to treat a patient with AMD.^745^ This study involved creating human iPSC-derived RPE monolayer sheets without the use of artificial scaffolds. These hiPSC-RPE sheets expressed ECM BMs and could be prepared for engraftment by laser microdissection, eliminating the need for in vivo maturation.^744^ In accordance with FDA and World Health Organization guidelines, iPSC-derived RPE cells were rigorously tested in animal transplantation models with serial dilutions, demonstrating a negligible risk of tumor formation.^746^

Another promising iPSC therapy, known as iPLAT1, has successfully completed a first-in-human clinical trial.^747^ iPLAT1 is an iPSC-derived platelet product designed for transfusions in patients with thrombocytopenia and alloimmune platelet transfusion refractoriness due to alloreactivity against donor HLA-1 or platelet antigens. To create this product, iPSCs were derived from patient PBMCs. During differentiation, inducible transgenes (c-Myc, Bmi1, and Bcl-xl) were used to immortalize megakaryocyte progenitors, which were then expanded in a turbulent flow bioreactor with a cocktail of adhesion-independent drugs (Y-39983, GNF-316, and KP-475).^748^ The transfusion was successful, with detectable circulating large platelets and only minor side effects.^747^ This same research group has also developed an “off-the-shelf” iPSC-derived platelet product depleted of HLA class I, intended for clinical use.^749,750^

The use of hydrogels to generate iPSC-based therapeutic products has shown potential in animal models across various tissues, including the kidney,^751,752^ liver,^753,754^ CNS,^755^ PNS,^756^ heart,^757,758^ bone,^759^ thymus,^760^ pancreas,^761^ and intestine,^762^ among others. For example, in mouse models of MI, iPSC-derived cells embedded in hydrogels have demonstrated cardiac repair and improved function following intrapericardial^757^ and intramyocardial injection.^758^ In a thymus model, human iPSCs embedded in 3D alginate hydrogel capsules with hematopoietic progenitor cells were differentiated into iPSC-derived thymic epithelial cell organoids.^759^ When transplanted into a humanized mouse model, these iPSC-derived thymic epithelial cell organoids supported human T cell development.

In a cranial bone regeneration study, iPSC-derived neural crest mesenchymal progenitor cells embedded in Ink-Bone (CELLINK Life Sciences) were shown to increase bone volume and promote partial bone bridging, indicating that iPSC-derived cells maintained osteogenic potential when extruded as a bio-ink into a cranial defect.^760^ Additionally, the use of a neurotrophin-enriched (GDNF and BDNF) collagen hydrogel with iPSC-derived dopaminergic neuron progenitors improved survival and differentiation, as evidenced by increased tyrosine hydroxylase immunostaining, compared with cells transplanted into PD rat brains without a supportive neurotrophic scaffold.^763^

Hydrogels have demonstrated significant potential in promoting regeneration following spinal cord injuries (SCIs). For example, in a rat model of cervical spinal cord contusion^764^ and a mouse model of spinal cord transection,^765^ transplantation of hydrogels embedded with iPSC-derived deep cortical neurons or iPSC-derived neuronal stem cells, respectively, facilitated neural regeneration and functional recovery. Moving beyond the limitations of hydrogels, which can clump in vivo, and 3D printing methods that can disintegrate upon transfer, a novel technique for creating cellular constructs with embedded vascular structures has been developed.^766^ This method involves the self-assembly of iPSC-derived endothelial cells within a fibrin matrix, forming microvascular meshes around micropillars. This configuration allows precise control over the shape and size of the construct, up to an area of 5 cm^2^. Similar to stem cell—derived sheets designed for transplantation,^767^ these microvascular meshes provide a more organized and stable vascular structure.^766^ In vivo experiments have shown that subcutaneous transplantation of iPSC-derived microvascular meshes alone promotes neovascularization and anastomosis. When cotransplanted with rat islets, these meshes enhance islet vascularization and achieve a 3-month reversal of T1D in a mouse model. This approach aims to incorporate a vascular framework that is often missing in other methods, thereby supporting engraftment, differentiation, and graft survival.

Advancements in bioengineering are developing new techniques for introducing iPSC-derived cells into host tissue for cell therapy applications while maintaining cell viability. Another notable achievement in iPSC engineering involves the treatment of T1D using a retrievable graft. Researchers utilized a human iPSC line (TkDN4-M) transformed into *β* cells through a 6-stage chemical reprogramming method, which involved the sequential use of small molecules and growth factors to guide differentiation from definitive endoderm to *β* cells. These iPSC-derived *β* cells were encapsulated in a lotus root—shaped construct filled with hydrogel suspension.^768^ Another iPSC engineering feat came from a group looking to treat T1D with a retrieval graft. Using a human iPSC line (TkDN4-M) transformed to *β* cells through a 6-stage chemical reprogramming method using small molecules and growth factors (1, definitive endoderm; 2, primitive gut tube; 3, posterior fore gut; 4, pancreatic progenitor; 5, endocrine progenitor; and 6, *β* cell), they filled a lotus root—shaped cell-encapsulated construct with iPSC-derived *β* cells in a hydrogel suspension.^769^ This tube-filled structure facilitated glucose, oxygen, and insulin exchange with the host mouse for over a year after transplantation and remained easily retrievable after 1 year.

The use of chemical small molecules to differentiate iPSCs into functional target cells with physiological outcomes in mammals has gained considerable attention in recent years. For example, iPSC-derived cells are being optimized for treating neurodegenerative diseases through the transplantation of healthy, functional neurons.^770^ Cortical neurons have been successfully derived from hPSCs (hESC and fibroblast-derived iPSC) using a combination of small molecule inhibitors (LDN193189, SB431542, XAV939, PD0325901, SU5402, DAPT, and CHIR99021) during differentiation.^771^ These neurons, possessing functional electrophysiological properties, can develop long-distance projections when transplanted into the postnatal mouse cortex. Similarly, small molecule—derived cerebral organoids have been used to develop human cortical glutamatergic neurons with functional electrophysiology.^772^ In a study involving patients with Down syndrome, iPSCs were differentiated using small molecule inhibitors (TGF-*β* inhibitors SB431542, BMP inhibitor DMH-1, and hedgehog inhibitor cyclopamine).^773^ Following microtransplantation into the mouse cortex, iPSC-derived cortical neuron organoids matured, extended projections to the basal brain region, formed bidirectional synaptic connections with pre-existing mouse neurons, and improved auditory stimuli response.^772^ Another study on PD used small molecules (ascorbic acid, SB431542, dorsomorphin dihydrochloride, purmorphamine, CHIR99021, and Y27632) to differentiate iPSCs into functional nigral dopaminergic neurons. When transplanted into the striatum of 6-hydroxydopamine neurotoxin—lesioned rats, these neurons rescued motor defects and demonstrated axonal outgrowth.

Research has also focused on using small molecules to differentiate iPSC-derived human islets. A combination of small molecules (activin A, vitamin C, CHIR99021, PI103, Y27632, SB431542, LDN193189, Wnt-C59, nicotinamide, ISX9, ALK5 inhibitor II, and forskolin) administered in 6 sequential stages successfully generated functional hiPSC-islets with maturation markers (MAFA and UNC3) and biphasic insulin secretion.^774^ These hiPSC-islets restored insulin secretion and glycemic control in diabetic nonhuman primates following a single intraportal infusion. In kidney capsule transplantation experiments, iPSC-derived kidney organoids generated using small molecule—based differentiation (Y-27632, CHIR99021, heparin, Noggin, activin A) effectively served as surrogates for human kidneys.^775^ Single-cell analysis revealed that these iPSC-kidney organoids could reduce off-target cells after transplantation.

Efforts to make iPSC-derived cells safer for human cell therapy have led to the development of chemically defined, animal origin—free (CD-AOF) differentiation media. Using CD-AOF, researchers have generated iPSC-derived liver bud organoids that rescued acute liver failure in a mouse transplantation model.^776^ This approach eliminates the risk of introducing infectious or unpredictable factors from animal sources. Additionally, xeno-free plastic scaffolds are being developed for iPSC-derived cell transplantation to further enhance safety.^777^ Collectively, these studies represent significant progress in developing iPSC-based therapies for neurodegenerative diseases, diabetes, liver, and kidney diseases using small molecules. Combining small molecule indirect cellular reprogramming with CD-AOF culture conditions is a critical step toward creating viable, functional, and safe iPSC-based cell therapies for humans.

DMD is a severe muscle-wasting disease caused by a mutation in the *Dp427m* gene that prevents muscle cells from producing dystrophin; currently, there is no cure for DMD.^778^ Researchers are exploring iPSC-based cell replacement as a potential therapeutic approach. A recent study in a DMD mouse model demonstrated that hiPSC-derived myogenic precursors expressing conditional PAX7, a chromatin remodeling factor, were sufficient to restore dystrophin expression and improve force generation.^779^ Following intramuscular transplantation, these hiPSC-derived myogenic precursors successfully engrafted, integrated into the muscle satellite cell pool, and persisted for half of the animals’ lifespan, highlighting the feasibility and potential of iPSC-based treatments for DMD. In addition to general skeletal muscle degeneration, diaphragm weakness and respiratory failure are major contributors to mortality in patients with DMD.^778^ To address the respiratory complications associated with diaphragm dysfunction in DMD, a recent study investigated iPSC-based transplantation therapy.^780^ In this study, hiPSC-derived MuSCs were generated using the small molecules CHIR99021 and SB431542 in culture. These iPSC-derived MuSCs were then directly injected into the diaphragms of DMD mice. This research was the first to demonstrate the feasibility of direct cell transplantation into the diaphragm, although it was reportedly very challenging. The transplanted cells that engrafted were able to regenerate dystrophin-expressing skeletal muscle. However, the engraftment efficiency was low, mainly due to the technical difficulties of the injections and the lower proliferative potential of iPSC-derived MuSCs compared with primary cells. While this study represents a promising step toward developing an iPSC-based therapy for DMD-related respiratory issues, further optimizations will be necessary before clinical applications are viable.

An ongoing clinical trial for SCI is using integration-free “HLA super-donor” iPSC cell lines, which are differentiated into neural stem/progenitor cells for grafting.^781^ Unlike the iPLAT1 trial, the treatment of SCI requires rapid intervention, making the timing of cell production crucial. Owing to the urgency following SCI, there is not enough time to generate patient-specific iPSCs. Instead, these cells are produced in a Good Manufacturing Practices facility and then frozen and stored until a suitable recipient is identified. A significant focus during this process is the selection of neural progenitor cell clones that lack tumorigenic properties.^782^ Researchers are optimistic about the potential of treating patients with SCI with iPSC-derived neural progenitor cells from the HDF-originating iPSC cell line 201B7. These cells have demonstrated the ability to differentiate into mature neurons after transplantation, produce neurotrophic factors, form synaptic connections, and generate oligodendrocytes in supportive animal model trials.^783, 784^

Combining iPSC reprogramming strategies with other therapeutic treatment options is also gaining traction as they have the potential for synergistically promoting beneficial outcomes. For example, a phase I trial demonstrated that treatment with iPSC-derived NK cell line FT500 combined with immune checkpoint inhibitors stabilized or reduced lesion size in patients with advanced solid tumors or lymphomas, without severe adverse events or graft-versus-host disease response.^785^ Combining iPSC-derived cells with CAR-NK cell differentiation (CAR-iNK), tyrosine kinase inhibitors, radiotherapy, or anti-PD-L1 monoclonal antibody has provided encouraging outcomes in the management of cancer outcomes.^786–788^ The combination of iPSC-derivation with CAR-NK directed differentiation improved the homogeneity of the resulting CAR-iNK cell population while improving safety and cell product specificity. Interestingly, in a mouse model, fibroblast-derived iPSCs were used in a cancer vaccine as an adjuvant sharing multiple cancer cell epitopes, resulting in boosted cancer immune response and reduced evasion of immune surveillance.^789^ This method has the potential of developing patient-specific prophylactic cancer vaccines that promote an antigen-specific T cell response for multiple cancer types.

iPSCs have been used in multiple therapeutic combinations to improve specificity and bolster outcomes compared with more traditional approaches. In addition to cancer immunotherapies and vaccines, iPSCs are being developed as patient-specific sources for transplantation of autologous cells treated with gene therapy, in animal models^790–792^ and human studies.^793,794^ These advances in combination therapies with iPSCs show great translational potential. Given the inherent oncogenic risks associated with stem cell—derived cells and viral vector integration events, adherence to stringent quality control and monitoring will be necessary.

iPSC clinical research has been steadily gaining momentum over the past decade with an upsurge of iPSC clinical studies initiated between 2019 and 2020 reaching 25 studies, compared with 5 newly initiated clinical studies the previous year.^795^ This surge in clinical trials corresponds with a steady rise in iPSC patent publications, which peaked at 1705 in 2020.^796^ In 2022, there were 81 iPSC clinical trials conducted worldwide, including ongoing and completed studies. These trials span a diverse range of biological systems, including the nervous, respiratory, circulatory, genitourinary, visual, and endocrine systems, as well as the skin, blood, neoplasms, and developmental defects.^797^

Countries like Japan, with less stringent restrictions on the clinical use of iPSC technology, have seen some remarkable successes. In Japan, iPSC-derived tissue sheets have been approved for clinical use as a treatment for heart disease.^798^ Additionally, Japan made headlines when a woman became the first person to receive skin-derived iPSC-derived corneal cells to improve her vision.^799^ Following this success, Japan approved a clinical research trial (jRCTa050200027) to treat patients with allogeneic iPSC-derived retinal sheets. In 2015, autologous iPSC-derived dopamine neurons were injected into the midbrains of cynomolgus monkeys as a model for PD therapy, which led to improved motor function that persisted for 2 years without the need for immunosuppression.^800^ Building on this success, a preclinical study using clinical-grade hiPSC-derived dopaminergic neurons for PD demonstrated safety in mouse models.^801^ An ongoing clinical trial in Japan (UMIN000033564) is using iPSC-derived dopaminergic progenitor cells to treat PD, with the first patient success reported in 2018.^802^ These advancements represent significant progress in regenerative medicine and are likely to shape future therapeutic possibilities.

### C. Limitations of indirect reprogramming

Using cell plasticity and stemness properties through iPSC reprogramming techniques represents a promising area in regenerative medicine and disease therapy, as well as in disease modeling.^803^ Among the various methods for generating somatic-derived iPSCs, those that are free of transgene sequences—known as zero-footprint methods—are preferred to maximize patient safety.^728^ However, even under highly controlled conditions, there remains a risk of tumorigenicity^804^ or triggering an immune response.^774,805^ To mitigate the risk of immune reactions from allogeneic donor iPSC-derived transplantations, one approach is to mask the presence of the transplanted cells with syngeneic MSCs. In a model using iPSC-CMs, syngeneic MSCs were cotransplanted with allogeneic iPSC-CMs. This cotransplantation strategy resulted in extended graft survival and enhanced immune tolerance, likely due to cell—cell signaling and potential nanotube-exchange interactions with activated lymphocytes.^806^

Autologous iPSC-derived cells, although generally safer than allogeneic donor-derived cells, still possess the potential for immunogenicity when reintroduced into the original patient.^807–809^ The immune response elicited can vary depending on the type of cell being transplanted; for example, autologous hiPSC-derived smooth muscle cells tend to be more immunogenic than autologous hiPSC-derived retinal pigment cells.^810^ One underlying cause of this immunogenic potential is linked to mitochondrial DNA (mtDNA) mutations. It is reported that the reprogramming process increases DNA mutation rates, which are typically lower than those for mtDNA.^811,812^ This has led to the discovery that single-nucleotide polymorphisms in mtDNA within autologous iPSC-derived cells can produce neoantigens that are sufficient to trigger an HLA-dependent immune response upon transplantation back into the host.^813,814^ Therefore, additional screening for mtDNA mutations is advisable for iPSC manufacturers to mitigate this risk. To address the challenges of immune rejection in iPSC production, hypoimmune-iPSC (HIP) have been developed. These cells are engineered with the deletion of HLA class I and II molecules and the overexpression of CD47, a “do not eat me” signal commonly used by malignant cells to evade the immune system.^815–817^ HIP (B2M^−/−^CIITA^−/−^CD47^+^) pancreatic islet cells have shown significant promise in immune evasion, surviving in allogeneic nonhuman primate recipients for 40 weeks after transplantation without the need for immunosuppression.^815^ Previous studies have demonstrated that depleting B2M and CIITA alone or in combination in iPSCs can promote immune evasion, but this approach is not entirely effective without the additional overexpression of CD47.^818,819^ If successfully applied in humans, HIP iPSCs could provide a powerful solution for replenishing insulin-producing *β* cells in patients with advanced T1D.

Reprogramming efficiency and genomic integration are critical considerations in iPSC production and ensuring clinical safety. Low reprogramming efficiency can result in undifferentiated cells, intermediate products, or tumorigenic transformed cells, all of which pose significant risks to patients. Additionally, genomic integration can lead to oncogene activation, further increasing the risk of tumorigenesis.^449,820,821^ Initial reprogramming efficiencies using the OSKM factors were relatively low, ranging from 0.02% to 0.08%.^54,822^ Attempts to generate iPSCs using nonintegrating adenovirus significantly reduced reprogramming efficiencies to between 0.0001% and 0.001% of input cells.^823^ Replication-incompetent AAV, frequently used in clinical trials, has a reported reprogramming efficiency of less than 0.01%, with a rare chance (<10%) of genomic integration.^824, 825^ Sendai virus, on the contrary, is considered a safer method for producing clinical grade iPSCs due to its cytoplasmic replication and nonpathogenicity in humans.^826^ It has a reprogramming efficiency of 0.01%–4%, although it may require up to 20 passages to completely remove the viral genome and multiple viruses if delivering multiple factors.^826,827^ In the process of iPSC production, reproducibility remains challenging. Numerous factors such as human contamination, cell line-to-line variation can contribute to reproducibility issues. Some of these issues may be addressed by semiautomated or fully automated iPSC culture workstations,^828,829^ AI-based cell sorting microscopy,^830^ and sorting techniques including the use of magnetically labeled antibodies to generate a more homogeneous iPSC product.^831^ Such advances taken together with adherence to stringent quality control requirements, for example, those recommended by the Global Alliance for iPSC Therapies^832^ will help usher in a major platform technology enabling clinically beneficial patient-specific iPSC therapies.

Nonviral methods such as transposons, including PiggyBac and Sleeping Beauty, offer reprogramming efficiencies of 0.02%–0.05% but carry inherent tumorigenic risks due to host—genome integration.^729,827^ Nonviral mRNA transfection with VPA achieves a higher reprogramming efficiency of 4.4%, but this method is more labor intensive, requiring daily transfections rather than the single transfection or transduction needed for other methods.^720,833^ miRNA transfection methods have reported reprogramming efficiencies of 0.002%, while using miRNAs as lentiviral particles can achieve a robust reprogramming efficiency of nearly 10% in fibroblasts with Hdac2 suppression.^827,834,835^ Other methods such as minicircle vectors, episomal plasmids, and protein expression have very low reprogramming efficiencies, ranging from 0.005% to 0.006%, and require daily transfections or complex synthesis processes.^729,827^

To avoid issues of genetic integration and viral vector remnants, researchers have increasingly turned to chemical induction using small molecules to produce iPSCs.^836–838^ However, concerns remain regarding the underlying mechanisms, gene regulation dynamics, and the lack of specificity in chemical targeting, which could result in unintended cell fates.^839,840^ The use of BrdU in chemical reprogramming has further raised concerns about chromosomal instability, increased mutational burden, toxicity, and unknown cell fate dynamics.^841,842^ Although BrdU has been used to study neurogenesis in some patients,^206^ it has also been shown to cause dose-dependent genetic and neurologic developmental defects in animal models.^843,844^ Given the rapid advancements in cell reprogramming, stringent safety evaluations for each component of reprogramming cocktails are essential.

Novel techniques continue to be developed to generate iPSCs with minimal risk to patients. One promising approach is the use of CRISPR/Cas9-based systems to activate endogenous transcriptional reprogramming genes. Early attempts using the CRISPR synergistic activation mediator system or dCas9-VP64 to activate LIN28, OCT4, SOX2, with or without KLF4 and MYC in primary human fibroblasts, were unsuccessful in generating embryonic stem-like colonies during iPSC reprogramming.^845^ However, combining CRISPR activation (CRISPRa) with small molecules has shown promise. The SunTag CRISPRa system, combined with small molecules PD0325901, CHIR99021, thiazovivin, and SB431542, successfully reprogrammed human fibroblasts to iPSCs by activating OCT4, SOX2, MYC, LIN28, NANOG, and KLF4 (or EEA-motif).^846,847^ This method avoids the need for activating the oncogene *KLF4* and maintains better genomic stability by circumventing the knockdown of P53 by short-hairpin RNA or miR-302/367 used in other protocols.^848^

Regardless of the production method, most countries enforce stringent federal regulations and mandate comprehensive testing for hPSC markers, genetic stability, vector clearance, differentiation capacity, teratoma-forming potential, sterility, and viability.^849^ The primary objective of hPSCs in clinical applications is to produce cells that, upon introduction into a host environment, can perform a physiological function, such as regenerating damaged tissue or combating a disease state. The selection of donor iPSC source cells, as well as determining the appropriate dose or number of cells administered, can significantly impact the physiological outcomes and treatment efficacy. Administering too few cells may be insufficient to achieve a measurable physiological effect or to ensure long-term engraftment.^742,850^ Cell plasticity and stemness are influenced by environmental cues, enabling the cells to adapt and fulfill a physiological role. In some cases, introducing precursor cells, which are not fully differentiated, through cell transplantation or iPSC therapy allows for final differentiation into specific lineages to occur in vivo. This approach has been associated with beneficial physiological outcomes in treating conditions such as nerve injuries and other diseases.^782,850,851^

## VII. Direct reprogramming for cell-based and tissue-based therapies

**Fig. 8.**
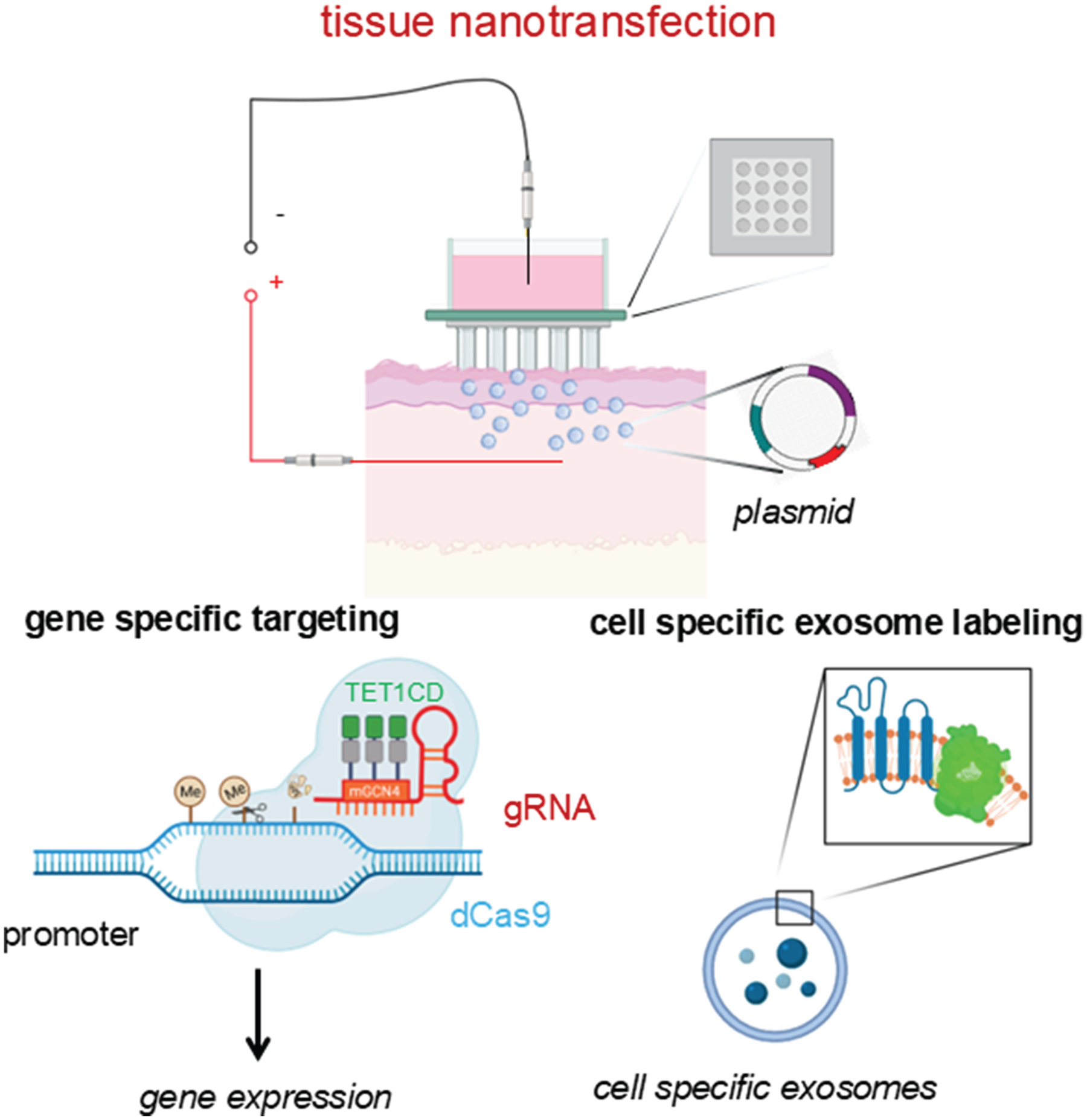
TNT. Cell-specific and gene-specific intervention in vivo. TNT enables the delivery of charged molecules (eg, ASOs or plasmids), directly into specific regions of the skin in vivo. TNT may also achieve cell and gene-specific editing. gRNA, guide RNA.

During the 1980s and 1990s, experiments involving cell fusion and nuclear transfer demonstrated that the cellular environment significantly influences cell identity, providing a foundational understanding for later developments in direct reprogramming. In 2010, Vierbuchen et al^685^ at Stanford University achieved a groundbreaking advancement by demonstrating that mouse fibroblasts could be directly reprogrammed into functional neurons through the introduction of 3 neural-specific transcription factors: Ascl1, Brn2, and Myt1l. This was the first definitive evidence of direct lineage conversion in mammalian cells.^685^ A significant progression in the field has been the development of in vivo reprogramming, wherein cells are directly reprogrammed within a living organism rather than in vitro.^321^ In 2012, Qian et al^852^ demonstrated that reprogramming factors could be delivered to nonmyocyte cells in the murine heart to generate cardiomyocyte-like cells, using viral gene delivery methods. However, viral reprogramming poses risks, including potential genomic integration events and unintended reprogramming of other cells, which could lead to serious adverse effects. In 2017, a nonviral direct reprogramming method was achieved using a nanoelectroporation technique known as TNT (Fig. 8).^321,853^ This technique successfully achieved both vasculogenic and neurogenic reprogramming of the skin.^112,172,854^

Direct reprogramming is distinct from indirect reprogramming primarily because it does not require an intermediary iPSC state.^855^ When direct reprogramming is successful, it produces cells with the intended functionality, without reverting to an intermediate cell type. This bypasses the creation of iPSCs, whose uncertain fate can pose significant risks, such as the potential for tumorigenesis. Direct reprogramming strategies typically target precursor or fully differentiated somatic cells to transdifferentiate into a desired functional cell type. By avoiding the iPSC stage, direct reprogramming reduces the risk associated with the potential presence of tumorigenic cells in the final product.^855,856^ One of the main advantages of direct reprogramming is the ability to reprogram cells in vivo through single-step techniques.^112,321,696,853,857^ As with indirect reprogramming methods, direct reprogramming has also benefited from the use of small molecule compounds that can supplement, enhance, or replace other components, resulting in safer and more functional transdifferentiated cell products.^687^

### A. Significance of direct reprogramming

Direct reprogramming has its conceptual origins in the pioneering work of John Gurdon during the 1950s and 1960s, where SCNT was used to demonstrate that fully differentiated somatic cells retain the genetic information and capacity to differentiate into other lineages.^49–51^ This groundbreaking research showed that differentiated cells possess a degree of plasticity, maintaining the inherent potential to change into different cell types. This concept was further explored and expanded in 1979 by Taylor and Jones, who demonstrated the plasticity of fully differentiated cells in vitro. By culturing mouse fibroblasts with varying concentrations of 5-azacytidine, they successfully converted the 10T1/2 fibroblast cell line into monocyte, adipocyte, and chondrocyte cells.^858,859^ Their work provided evidence that differentiated cells could be reprogrammed into distinct lineages, with the newly formed cell types expressing specific gene sets characteristic of their identities.^860^ In 1987, Davis et al^861^ advanced the field of cellular reprogramming by isolating the cDNA sequence for a myoblast-specific transcription factor, MyoD1, and transfecting it into terminally differentiated fibroblast cells. This led to the conversion of fibroblasts into stable myoblasts.^861^ This pivotal study demonstrated that cell identity could be altered through the introduction of a single transcription factor, significantly influencing subsequent research, including the discovery of iPSCs using the OSKM transcription factors by Takahashi and Yamanaka. Moreover, it inspired the concept that somatic cell transdifferentiation could be achieved with minimal genetic manipulation.

These foundational studies have been instrumental in developing the concept of direct reprogramming, or transdifferentiation, where fully differentiated cells are converted from one specific fate to another without passing through an intermediate pluripotent state. Direct reprogramming is now a valuable tool in regenerative medicine, with applications across a wide range of model systems and therapeutic contexts.^4,112^ A primary goal within the fields of direct reprogramming and regenerative medicine is the translation of these techniques into clinical practice.^856,862–864^ The ability to treat diseased organs without surgical intervention, restore cell populations depleted by disease, or enhance wound healing through in vivo direct reprogramming could significantly expand the boundaries of modern medicine. As various disciplines and research avenues converge on both direct and indirect cellular reprogramming, the potential for clinical applications in the near future appears not only feasible but increasingly likely.

### B. Progress in direct reprogramming

Recent studies have increasingly used ex vivo direct reprogramming to expand and transdifferentiate cells for transplantation and cell replacement therapies, particularly in situations where donor cells and organs are scarce (Table 4).^865–879^ The ability to grow and expand cells ex vivo for autologous transplantation, while avoiding the risks associated with iPSC intermediates, is a compelling approach for certain ex vivo applications. This concept has also gained significant attention in the field of small molecule reprogramming, where it minimizes or eliminates the risks of viral remnants and genomic integration.

#### 1. Ex vivo and transplant

Severe liver failure resulting from damage or disease is a serious condition that typically requires transplantation as the only curative option. Unfortunately, the availability of donor organs is limited and carries inherent risks of immune rejection. The ability to produce suitable hepatocytes in large quantities for cell replacement therapy has been a significant challenge, which direct reprogramming may help overcome. To address this, researchers have developed human-induced hepatocytes (hiHEPs) that can be generated in vitro through the direct reprogramming of fibroblasts. Using lentiviral transduction to introduce the transcription factors FOXA3, HNF1A, and HNF4A, fibroblast-derived hiHEPs were produced, which were capable of restoring liver function and prolonging the survival of mice with acute liver failure, although the reprogramming efficiency was low.^624^

Recent advancements in nonviral reprogramming methods have led to the development of chemical reprogramming techniques. For example, a small molecule cocktail—comprising CHIR99021, E-616452, forskolin, AM580, and EPZ004777 or CHIR99021, E-616452, forskolin, CH55, UNC0638, and EPZ004777—was used to chemically induce mouse fibroblasts into a cell-state reminiscent of hepatocytes. Upon transplantation into mice with liver failure, these chemically iHeps repopulated the liver, exhibiting activity and function similar to primary hepatocytes. While this chemical induction method reduced the risks associated with viral transduction, the reprogramming efficiency remained low at 0.8%.^470^ More recently, another small molecule cocktail—SB431542, LDN193189, BIX01294, CHIR99021, and DAPT—achieved a significantly higher reprogramming efficiency of up to 80%.^473^ This cocktail directly reprogrammed MEFs into iHeps that, upon transplantation, promoted liver regeneration and prolonged survival in mice with acute liver damage. These iHeps demonstrated gene expression patterns similar to mature primary hepatocytes and showed no tumorigenic potential in both in vivo and in vitro assays.^473^ With these rapid advancements in nonviral direct reprogramming methods to produce transgene-free iHeps, translation to clinical regenerative medicine appears imminent.

As discussed in earlier sections, various reprogramming methods have been explored for regenerating damaged cardiac tissue following MI. While iPSC approaches have made significant strides in design and safety,^880^ there remain concerns about the teratoma-forming potential and genomic instability associated with cells passing through a pluripotent intermediate. Recent progress in direct reprogramming for cardiac transplantation has shown promising outcomes. For instance, adipose-derived regenerative cells (ADRCs), transfected via a lentiviral construct with a unique combination of 6 transcription factors (Baf60c, Gata4, Gata6, Klf15, Mef2a, and Myocd), generated presumed cardiomyocytes (6F-ADRCs).^875^ Single-cell RNA sequencing (scRNASeq) bioinformatics analysis revealed that these 6F-ADRCs had upregulated the expression of cardiomyocyte-specific genes (Myh6, Actc1, and Tnnt2). When transplanted into infarcted heart tissue in vivo, the 6F-ADRCs survived for extended periods, promoted angiogenesis, likely through VEGF-A/B paracrine factor expression, and improved cardiac function by preventing left ventricular remodeling. These findings suggest a fate change to the cardiac lineage.

In a similar approach, skin fibroblasts were directly reprogrammed into cardiomyocytes using a combination of cardiac transcription factors (GATA4, MEF2c, and Nkx2.5).^877^ Rat skin fibroblasts were transfected with reprogramming factors via plasmid electroporation and transplanted into an MI rat model. Pretransplantation gene and protein expression analyses (GATA4, cMHC, MEF2c, Nkx2.5, cTnI, and TNNT2), along with post-transplantation histological, immunohistochemical, and echocardiographic assessments, confirmed that these cells had transdifferentiated into the cardiac lineage and promoted myocardial regeneration. Interestingly, when iPSC-derived cells were transplanted, they showed reduced responses in most metrics compared with the directly reprogrammed cells, likely due to the reversion of cells to an embryonic-like stage rather than direct differentiation.

In a small molecule—based approach to cardiac reprogramming, fibroblasts differentiated via small molecules (CHIR99021, A83-01, GSK126, Forskolin, CTPB, and AM580) under xeno-free conditions generated cardiovascular progenitor cells capable of improving heart function in infarcted mice for up to 13 weeks after transplantation.^486^ In another strategy, 3D heart ECM and small molecules were used to directly reprogram fibroblasts into cardiomyocytes.^525^ Primary MEFs were cultured in 3D decellularized heart ECM-derived hydrogel and treated with small molecules (CHIR99021, A83-01, forskolin, and SC-1) before transplantation into a MI rat model. The resulting cardiomyocytes exhibited upregulated cardiac markers, electrophysiological function, sarcomere organization, and responsiveness to *β*-adrenergic agonist treatment. Using heart ECM hydrogel in transplantation to MI rat hearts facilitated cell—ECM interactions that promoted cardiomyocyte maturation and integrin signaling in a 3D microenvironment, resulting in improved neovascularization and reduced fibrosis compared with transdifferentiated fibroblast—derived cardiomyocytes in 2D mats. However, this method’s limitation is that reprogrammed cardiomyocytes more closely resemble neonatal rather than adult cardiomyocytes. Optimizing maturation may be achieved through the addition of bioelectric fields or incorporating biomaterials into the scaffold structure. Overall, these approaches to producing functional cardiac lineage cells demonstrate that incorporating multiple direct reprogramming methods enhances the functionality and utility of the end products for treating MI-injured heart tissue.

Diseases like multiple sclerosis cause neurological dysfunction due to CNS demyelination and oligodendrocyte loss. Developing oligodendrocyte progenitor cells (OPCs) via direct reprogramming capable of differentiating into functional oligodendrocytes has made significant progress in recent years. MEFs chemically reprogrammed with a cocktail of 9 factors (CHIR99021, LDN193189, A83-01, Hh-Ag1.5, retinoic acid, SMER28, RG108, Parnate, and bFGF) have been shown to transdifferentiate into induced OPCs with verified molecular and functional assays.^503^ Moving closer to clinical application, another group recently reported that human astrocytes cultured with a cocktail of 6 small molecule compounds (CHIR99021, forskolin, RepSox, LDN, VPA, and thiazovivin) also transdifferentiated into induced OPCs.^529^ These induced OPCs were then transplanted into demyelinated mouse brains, where they successfully differentiated into mature oligodendrocytes within the brain microenvironment. These nonintegrating direct reprogramming methods represent a promising new avenue for treating patients with debilitating neurologic diseases.

#### 2. In vivo

One of the most promising aspects of direct cell reprogramming is its potential to regenerate diseased or damaged tissues within the living body, eliminating the need for transplantation (Tables 1 and 5).^878–895^ Efforts are underway to enhance in vivo reprogramming efficiency and reduce associated risks. A significant advancement in this field is the development of nonviral methods for the targeted delivery of reprogramming factors, which enhances the safety of clinical applications. TNT is a notable example, achieving cell-specific delivery and gene editing.^321,696,853,854,857^

TNT is an electromotive gene transfer technology that was developed to achieve nonviral tissue reprogramming in vivo.^321^ This topical transcutaneous gene delivery system transports genetic factors across skin barriers and cell membranes in a uniform field, maintaining cell integrity and viability. TNT is a combination of gene therapy and technological advances designed to directly reprogram cells in their natural tissue habitat to support cell state/fate changes without the use of potentially harmful viral vectors. The initial version of TNT used nanochannels to deliver reprogramming factors from a solution in contact with a negative electrode, through exfoliated skin, to a positive electrode inserted intradermally. For example, similar to the in vitro reprogramming of MEFs to iNs using ABM overexpression,^685^ TNT was used to deliver ABM factors transdermally, directly reprogramming mouse skin fibroblasts into neuronal and neurogenic states.^172,321,853^ This nonviral method demonstrated that these factors could propagate beyond the uppermost skin layers through EVs carrying ABM cDNAs and mRNAs,^321,696,896,897^ reaching deeper dermal layers. Thus, TNT effectively uses the skin microenvironment as a self-sustaining bioreactor.^898^ Through this mechanism, TNT-ABM successfully transdifferentiated fibroblasts into functional iNs in a necrotizing tissue mouse model^321^ and a cutaneous diabetic polyneuropathy model.^172^ Additionally, altering the genetic factors delivered from ABM (fibroblast to iN) to Etv2, Foxc2, and Fli1 changed the transdifferentiation pathway, producing induced endothelial cells. Etv2, Foxc2, and Fli1 delivery via TNT was effective in converting fibroblasts into vasculogenic cells to rescue ischemic tissue.^854,899^

Fibroblasts, which exist in nearly every tissue, provide both structural and nonstructural support. Through bulk and single-cell sequencing, fibroblasts have been shown to exhibit significant heterogeneity, resulting in various subtypes that are conserved in mammals.^900–906^ Skin injury itself is a natural cue to trigger physiological reprogramming of wound-site fibroblasts to VFs capable of rapidly producing functional blood vessels.^112^ This is achieved by rapid inhibition of miR-200b.^112, 907^ Under limiting conditions, such as diabetes, such physiological reprogramming fails to execute and can be rescued by TNT improving diabetic wound healing.^112^ Using TNT, antisense oligonucleotides (ASOs) targeting miR-200b were electroporated into mouse diabetic wounds. Without ASO intervention, miR-200b inhibits hyperglycemia-induced VEGF expression.^908^ Inhibiting miR-200b in diabetic dermal fibroblasts via ASO promoted their transdifferentiation into an endothelial-like state, known as VFs.^112^ This endothelial-like fibroblast state is such that it retains some original fibroblast characteristics and therefore VFs are described as a state, but not fate, change of dermal fibroblasts. TNT-based genetic cargo delivery has been successfully used in various wound healing applications, including recovery from volumetric muscle loss,^331^ rescue of necrotizing tissue,^321^ wound closure,^167,909^ lymphedema treatment,^910^ ischemic reperfusion^911^ and targeting lethal tumors for regression.^912^ These diverse outcomes underscore the potential of this innovative nonviral gene transfer technology to be integrated into clinical practice.

At first glance, TNT may appear to have many off-target effects; however, the system is designed to target all cells within the localized transfection area, creating a new microenvironment that not only directly reprograms target cells but also fosters a supportive environment, similar to how ECM or synthetic hydrogels support directed differentiation. Through mechanisms such as cell—cell communication, including exosomes, TNT treatments can extend beyond the transfection boundary.^696,913^ This tunable ability^913,914^ to penetrate multiple tissue layers allows for the reprogramming of entire microenvironments, not just individual cells, to induce physiological changes. A potential clinical hurdle is the high voltages used in TNT, which could cause minor cellular damage. However, this concern might be mitigated by engineering larger silicon chip areas or using lower voltages with longer exposure times. Together, these examples of TNT-mediated fibroblast conversion demonstrate the potential of this novel, safe, nonviral direct reprogramming technology for treating wounds and various disease states.

Studies using viral gene delivery have demonstrated the remarkable feasibility of in vivo direct reprogramming. Retinal degeneration and vision loss caused by neurodegenerative diseases are typically irreversible in mammals. Inspired by non-mammalian vertebrates that can regenerate RGCs, researchers are targeting Müller glia (MG) for direct reprogramming to generate neurogenic MG-derived RGC progenitors. The approach to in vivo MG reprogramming is based on early in vitro experiments, such as those with Ascl1/Brn2/Isl1 and ABM, which successfully reprogrammed MEFs into iNs and RGCs, respectively.^685,686^ Adapting this protocol for in vivo applications, researchers have used 2 transcription factors (Ngn1/3) involved in neurogenesis.^342^ By delivering these factors via lentiviral vectors into the retinas of mouse pups, MG cells—as well as rods, bipolar cells, and amacrine cells—were directly converted into RGCs.

Another research group replaced the ABM cocktail with a different set of transcription factors (Islet1/Pou4f2/Ascl1) to create CRE-inducible mouse strains for monitoring MG to RGC reprogramming following NMDA (glutamate receptor) retinal injury.^330^ This transcription factor combination generated RGC-like cells that, while not fully mature, exhibited directed axonal growth and mixed electrophysiological properties. Thus, while these cells were reprogrammed to an RGC state, they did not achieve full RGC maturation. Through scRNASeq and transposase-accessible chromatin sequencing, the researchers discovered that the transcription factors Islet1/Pou4f2/Ascl1 did not activate the complete set of genes required for RGC maturation as initially hypothesized. In a subsequent preprint publication, they employed high-throughput sci-PLEX scRNASeq to screen for compounds that might enhance MG to RGC reprogramming in injured mouse retinas.^345^ This screening identified DBZ and metformin as potential small molecules capable of regulating MG neuronal fate following Ascl1 induction. Although complex screening assays are not the ultimate goal, they represent substantial progress toward clinically viable in vivo reprogramming and the identification of small molecules that can facilitate this process.

Approaching MG reprogramming to regenerate RGCs from the perspective of intrinsic plasticity, another research group proposed that the MAP4K/YAP pathway limits the ability of mammalian MG to differentiate in damaged retinas.^333^ To test this hypothesis, they used a small molecule inhibitor of MAP4K4/6/7, DMX-5804, which was administered via intraperitoneal injection. In mouse retinas damaged by NMDA-induced injury, DMX-5804 promoted MG proliferation and conversion to a retinal progenitor cell—like state. Following the withdrawal of the inhibitor, these retinal progenitor cells transdifferentiated into retinal neuron—like cells, including amacrine and RGCs. This study exemplifies how combining concepts of inherent cell plasticity with small molecule reprogramming techniques can transdifferentiate targeted cell populations in vivo to treat diseases or damage-associated conditions. Beyond the studies mentioned earlier, which demonstrated the in vitro reprogramming of fibroblast cells to cardiac lineages prior to in vivo transplantation, several recent approaches have directly reprogrammed cardiac fibroblasts into cardiomyocytes in vivo with notable success. In a viral approach, transcription factors GMT were delivered via Sendai virus injection to the border between healthy and infarcted heart tissue, resulting in the direct reprogramming of cardiac fibroblasts into iCMs.^888^ Lineage tracing revealed a transition from fibroblast to cardiac lineage, which improved cardiac function and reduced fibrosis in mouse models of MI. However, this method was limited by a reprogramming efficiency of 2.5%, which persisted up to 4 weeks after transfection.

Another approach used a nanoparticle delivery system for cardiac reprogramming. Gold-coated nanoparticles conjugated with polyethylenimine and loaded with retroviral GMT vectors (AuNP/GMT/PEI) were injected into the MI heart border zone.^324^ Characterization of these cells showed successful reprogramming of cardiac fibroblasts into iCMs that upregulated cardiomyocyte-specific genes, displayed spontaneous contractility in vitro, and improved cardiac function after MI in vivo, achieving a reprogramming efficiency of 7%. Viral reprogramming poses risks of genomic integration, which is undesirable in a clinical setting. To mitigate these risks, nonviral nanoparticles have been used as miRNA carriers for cardiac reprogramming. Branched polyethyleneimine—coated nitrogen-enriched carbon dots (BP-NCDs) have been reported as an efficient miRNA delivery system.^328^ Epicardial injections of BP-NCD loaded with miRNA mimics (miRNA-1, miRNA-133, miRNA-208, and miRNA-499) into MI mouse hearts improved cardiac function by reducing fibrosis and enhancing ventricular wall thickness. Cardiac fibroblasts reprogrammed with BP-NCD-miRs in vitro showed silencing of fibroblast genes, increased expression of myocardial-specific genes and maturation markers (Myh7 and Tnnt2), and exhibited cardiomyocyte morphology, indicating a state-change in cardiac fibroblasts following direct reprogramming with BP-NCD-miRs nanoparticles. Unfortunately, the in vivo reprogramming efficiency was not reported.

Another nanoparticle approach involved using a FH peptide—modified neutrophil-mimicking membrane (FNLM) on mesoporous silicon nanoparticles as a miRNA (miRNA-1, miRNA-133, miRNA-208, and miRNA-499) delivery system.^327^ In vitro characterization of cardiac fibroblasts treated with FNLM-miRs showed upregulation of cardiac, sarcomeric, and ion channel genes, while fibroblast genes were suppressed. These reprogrammed fibroblast-derived cells demonstrated spontaneous beating and calcium flux, indicating successful reprogramming into iCMs. In an in vivo MI ischemia/reperfusion model, FNLM-miRs were administered intravenously, resulting in reduced fibrosis, improved cardiac function, and conversion of cardiac fibroblasts into iCMs with sarcomeric structures and TNNT2 expression, achieving a reprogramming efficiency of 1.5%. This successful conversion of cardiac fibroblasts to iCMs highlights the effective targeting capabilities of intravenously delivered miRNA-loaded nanoparticles. Although nonviral nanoparticle-based delivery systems have lower reprogramming efficiencies compared with Sendai virus (2.5%) and retroviral (5%–10%) gene delivery systems previously reported for GMT reprogramming of cardiac cells,^691,852^ they offer a safer, clinically applicable nonviral alternative.

In the CNS, the loss of neurons due to trauma or neurodegenerative diseases can be severely debilitating and is often irreversible.^915,916^ Astrocytes play a critical role in the development and maintenance of CNS neurons and are responsive to damage and neurodegenerative conditions.^917^ Given their proximity to neurons and their reactive nature, astrocytes are frequently targeted for direct reprogramming into neurons within the CNS. Numerous recent studies (Tables 1 and 5) have demonstrated the potential to reprogram astrocytes in vivo into neurons and neuron-like cells using various methods, genes, and small molecules. For example, the direct conversion of astrocytes into iNs has been achieved using the transcription factor NeuroD1 in several studies.^659,886,887^ In a nonhuman primate model of ischemic brain stroke, NeuroD1 delivered via an AAV was injected into the cortex. This AAV-mediated NeuroD1 delivery transdifferentiated 90% of infected reactive astrocytes into Tbr1^+^ cortical neurons, which survived for more than a year, with minimal off-target infection. As nonhuman primate models become better characterized and sequenced, it may be possible to confirm whether these astrocyte-derived cells have undergone a complete reprogramming of cellular fate. In a mouse model of ischemic stroke, lentiviral injection of NeuroD1 into the peri-infarct brain region successfully transdifferentiated astrocytes into NeuN^+^ neurons.^659^ Most iNs expressing the NeuN maturation marker also expressed the glutamatergic neuron marker vGLUT. Functionally, these iNs restored cortical circuits, correlating with improvements in psychological, motor, and sensorimotor functions, and exhibited electrophysiological properties (action potentials) and morphological characteristics (axonal outgrowth and neurite extension). These findings suggest that NeuroD1-iNs derived from astrocytes are likely undergoing a cell fate conversion from astrocytes to iNs.

NeuroD1 has also been used to reprogram astrocytes in the CNS in a mouse SCI model.^886^ In this study, AAV-based NeuroD1 was injected into the site of a spinal dorsal horn stab injury, effectively converting reactive astrocytes into iNs that expressed spine-specific genes and glutamatergic neuron markers. With a conversion efficiency of approximately 95%, functional assays confirmed the presence of neuron morphology, electrophysiological action potentials, and synaptic responses reminiscent of a cell fate change. Collectively, these experiments suggest that the tissue microenvironment (brain or spinal cord) influences cell fate specification, guiding the reprogrammed cells to develop into tissue-specific neuron subtypes (Tbr1^+^ cortical neurons in the brain and Tlx3^+^ neurons in the spinal cord).^659,886^

Astrocyte-to-iN conversion has also been achieved using nonviral small molecule reprogramming factor combinations. Small molecule—mediated conversion of spinal astrocytes to iNs is a nonviral, nonintegrative method developed to enhance the safety of potential clinical in vivo cell reprogramming therapies. Intraperitoneal injection of small molecules LDN193189 and CHIR99021 was shown to convert astrocytes to iNs, which expressed neuronal markers, exhibited neuronal morphology, and promoted neurogenesis in the injured spinal cord.^344^ These findings indicate that small molecules are sufficient to convert reactive astrocytes into a neuronal lineage state that can persist for up to a year in a damaged spinal cord. However, a trade-off was observed between increased safety, reduced specificity of the reprogrammed cell subtypes, and decreased reprogramming efficiency. Further refinement of the small molecule cocktail is needed to improve reprogramming efficiency and produce subtype-specific iNs capable of restoring motor function in damaged spinal cords.

Another small molecule reprogramming approach involved converting astrocytes to iNs in the brain using a cocktail of Forskolin, CHIR99021, ISX9, I-BET151, and Y27632, which produced iNs with electrophysiological function, synaptic connectivity, and neuron-specific marker expression in vivo (striatum, mostly GABAergic; cortex, mostly glutamatergic), supported by in vitro gene expression analysis.^326^ This in vivo small molecule—based method reported a reprogramming efficiency of 11%. In vitro direct reprogramming of astrocytes to iNs with small molecules (Y26732, DAPT, RepSox, CHIR99021, ruxolitinib, and SAG) achieved a higher efficiency of 82%.^521^ Lineage tracing confirmed the conversion of astrocytes to neuron lineage state; however, in vivo iN subtype specificity was more variable than in the previously described NeuroD1 viral approach.^326,659^ These studies demonstrate the feasibility of converting astrocyte populations in the mammalian brain and damaged spinal cord to neuronal lineage cell fates, potentially offering clinically safer, nonviral, and non-integrating treatments to restore functional neurons in patients with neurodegenerative diseases and traumatic injuries. However, small molecule-only reprogramming approaches require optimization to improve efficiency and narrow down the resulting iN subtype-specific products.

Considering the range of viral and nonviral direct cell reprogramming strategies, it is becoming increasingly evident that safer nonviral alternatives could replace viral-based delivery of reprogramming factors. This shift marks a significant progression in the fields of reprogramming and regeneration, demonstrating that cell fate transitions are possible without the risks associated with viral genomic integration. However, non-viral approaches are not without their own risks. Cell-type conversion specificity appears to be less precise than the outcomes observed with viral-based methods, likely due to differences in permeability and a lack of understanding of the underlying biochemical mechanisms altered when multiple small molecules are administered. Precision in delivery for both viral and nonviral methods is continually being improved to avoid off-target cell conversions. While viral vectors provide a relatively straightforward way to introduce genetic reprogramming factors into a host, they are not without risks. Viral constructs can integrate portions of their genetic sequences into the host genome, potentially leading to severe adverse outcomes, including oncogenesis, cell death, and the induction of autoimmune diseases due to aberrant protein synthesis.^447–449^

#### 3. Technical barriers to direct reprogramming

Direct reprogramming, similar to indirect reprogramming, has been performed using a variety of methods and factors, which can be categorized into 3 main approaches: ex vivo systems, transplantation of directly reprogrammed cells in vivo, and direct cell fate conversions occurring in vivo (Tables 3–5). Many of these methods use small molecule chemical compounds, either alone or in combination with other factors, to guide cells toward a desired fate while prioritizing clinical safety. However, there are still barriers to the clinical application of in vivo direct reprogramming. One significant challenge is the ability to track all cells undergoing reprogramming. This issue is partly technical, dependent on the method of reprogramming factor delivery and the potential impact on off-target cells.^4^ New delivery methods are being developed and optimized to minimize off-target cell conversion.^112,853,918,919^ Given the risk of off-target cell reprogramming by in vivo methods, lineage tracing is becoming a standard validation technique to confirm cell fate conversion.^920^ Without lineage tracing, results can be misinterpreted or skewed. Another consideration, as with indirect reprogramming methods, is reprogramming efficiency. While small molecule reprogramming offers a safer, nonviral, nonintegrative approach, there is often a trade-off in the specificity of the converted cell subtypes compared with viral-based and gene-based methods. Optimizing these small molecule cocktails and exploring the mechanistic and biological effects of small molecule combinations will make them more attractive therapeutic options in clinical settings.

The use of next-generation sequencing (NGS) has enabled in-depth transcriptomic analysis of how cells and cell populations respond to reprogramming factors. scRNAseq allows for the tracking of cells undergoing direct reprogramming as they transition from their initial cell type toward the targeted fate. For example, an NGS study tracked the progression of MEFs as they transitioned into iNs following Ascl1 overexpression. Early time points showed a mostly homogeneous response in MEFs, with upregulation of neuronal genes and downregulation of MEF genes, consistent with Ascl1 transfection efficiencies. Principal component analysis and quadratic programming analysis revealed a transitional transcriptional intermediate state before cells were fully committed to the neuronal lineage. This finding suggested that while Ascl1 is sufficient to initiate the conversion of a homogeneous population of MEFs toward a neural fate, additional reprogramming factors are needed to prevent cells from diverging toward nonneuronal, myogenic fates.^921^

Advanced computational methods combined with NGS technologies have provided not only enhanced resolution but also a means of predictive modeling to identify key regulators and insights for potential reprogramming factors. Analyzing the direct reprogramming of fibroblasts to induced endoderm progenitors using NGS and in silico gene regulatory network analysis revealed 2 trajectories: one toward successful endoderm progenitors and another representing reversion back to a fibroblast state. By combining in silico knockout simulations and experimental validation, researchers were able to identify factors that regulate reprogramming efficiency, as well as predictive factors and complexes that could further refine multiple related direct reprogramming cocktails.^922^ These advanced methods and refinements are among the tools that may help overcome the final hurdles preventing the widespread clinical application of direct cell reprogramming. Moreover, NGS and computational bioinformatics are not only propelling new reprogramming methods forward but also revisiting foundational techniques, such as the OSKM method by Takahashi and Yamanaka, to uncover transcriptomic complexities that had previously been overlooked.^923^

## VIII. Future perspectives

Revisions of the regulatory landscape as exemplified by the 2012 FDA Safety and Innovation Act (FDASIA) are expected to be seen more frequently to make room for novel approaches of healthcare. FDASIA established the “Breakthrough Therapy Designation” for drugs that demonstrate substantial improvement over existing treatments in preliminary clinical evidence. FDASIA improved the medical device review process by introducing the “Humanitarian Use Device (HUD)” pathway for rare diseases and simplifying premarket approvals. It made way for incorporating patient perspectives into the development and regulatory decision-making process. Broadening the use of surrogate endpoints (biomarkers that predict clinical benefit) for approving drugs targeting serious conditions represents another major advancement. Effective collaboration between academic and industry partners to improve the science behind drug development represents a major cornerstone as regulatory sciences evolves to support the new era in medical drug discovery. Infection complications represent a major threat to most forms of regenerative medicine therapies. FDASIA included the Generating Antibiotic Incentives Now Act, providing incentives like extended exclusivity for antibiotics treating serious infections. In sum, the 2012 FDASIA has been pivotal in modernizing the FDA’s regulatory framework, emphasizing speed, efficiency, and innovation.^924^ By introducing new pathways for accelerated approval, engaging patients, and incentivizing industry investment in high-priority areas, the act helped bring transformative therapies to market faster and more effectively, addressing urgent public health needs.

Thanks to leapfrogging capabilities in computation sciences, advancing at us at a rapid pace is a future that takes ATMPs to a new level of complexity. It is foreseeable that as we seek to navigate the biological complexities in vivo, we must increasingly rely on recent developments in ML and AI. AI-driven systems Deep-SeSMo and SSGraphCPI are being used to identify novel therapeutic agents in high-throughput drug screenings. Reinforcement learning and Bayesian AI-driven optimizations are being used to overcome the limitations and unknowns relative to small molecule and reprogramming factor treatments.^925,926^ The surging power of AI and ML is also being harnessed to identify epigenetic signatures and genes responsible for cell and tissue identity, which will provide a much-needed level of clarity for designing and implementing reprogramming strategies.^140,927,928^ As more training data become available, research methods will be increasingly driven by AI and ML.^541^ Integrating these technologies with precision will pave the future to personalized medicine wherein intelligent biomaterials and self-repairing “living bots” become a reality in clinical regenerative medicine therapies.^929^

As reprogramming strategies—both direct and indirect—continue to evolve, the integration of emerging picoscale technologies is poised to play a pivotal role in shaping the future of regenerative medicine. Recent advances in microfluidics have enabled precise manipulation of fluids at the pico—liter scale, pushing the boundaries of traditional organ-on-a-chip systems toward more versatile platforms such as capillary-driven laboratory-on-paper devices and channel-less laboratory-on-a-tip systems. These innovations offer unprecedented control over biological fluids and single-cell environments, facilitating high-throughput screening for drug discovery and biomolecular assays.^930^ Moreover, the development of microfluidic and nanofluidic systems capable of delivering nucleic acids and small molecules at near picoscale resolution is opening new frontiers in intracellular delivery.^931–933^ These platforms enable minimally invasive, highly targeted interventions that could be critical for reprogramming cells with high efficiency and low cytotoxicity. Complementing these advances, the emergence of picoscale particles—such as those based on graphene quantum dots—offers a promising nonviral alternative for gene and epigenetic modulation.^934^ Their biocompatibility, tunable surface chemistry, and ability to traverse cellular membranes make them candidates for in vivo applications. Emergent picoscale technologies are not merely incremental improvements; they represent a paradigm shift in how we interface with cellular systems. By enabling precise, scalable, and minimally disruptive manipulation of the cellular microenvironment, they are likely to become foundational tools in the next generation of cell and tissue reprogramming strategies.

To expedite the development and review of ATMPs, enabling patients to access innovative treatments more quickly, the RMAT designation was instituted by the FDA in 2017.^9,10,935^ RMAT applies to a broad range of therapies that include cell therapies, therapeutic tissue engineering products, human cell and tissue products, and certain gene therapies. Novel cell/tissue reprogramming, 3D bioprinting, and culture strategies are being developed to drive the field of precision medicine beyond cell therapies, toward fully cultured patient-specific organs for personalized transplantation, without the need for donors and their associated allogeneic risks.^936–939^ While manufacturing 3D-printed medical devices for transplantation carries risks of immune rejection, or cross-species contamination from xenogenic cell sources, regulatory bodies such as the FDA are being proactive in assessing and clearing such devices for clinical use.^940^ An adaptive regulatory framework will be necessary to make fully functional 3D-printed organs becomes a reality in healthcare.

Because of FDASIA, sponsors receive early and frequent interactions with the FDA to optimize product development strategies, including advice on clinical trial design and manufacturing. With over 100 products qualifying for RMAT designation, and 13 fully FDA approved, there is a recent shift in treatment options to incorporate a new wave of drugs and/or devices that specialize in cell and tissue reprogramming intervention.^6–8^ Fast-tracking ATMPs rely on preliminary clinical evidence wherein patient safety is of key significance. Both short-term and long-term follow-up observations monitoring patient health are necessary. For the treatment of hemophilia A, reprograming cells to express the missing factor VIII protein is achieved by valoctocogene roxaparvovec. Valoctocogene roxaparvovec treatment resulted in patients maintaining factor VIII protein expression with a half-life projected to last up to 5 years at levels supporting healthy to mild hemophilia. This is an RMAT designation therapy is delivered intravenously as liver-targeted AAV.^941^ Depending on longer-term observations, this treatment may replace the need for patients with hemophilia to receive multiple weekly intravenous factor replacement infusions. So called nonpathogenic AAVs are relatively safe but there remain some inherent risk factors for which effective nonviral gene delivery approaches are being investigated. FDA expects diligent monitoring of adverse events in response to the use of replication-competent viruses, such as retrovirus vectors, that include the risk of unregulated cell expansion and malignancy resulting from insertional oncogenesis events.^942^

From an implementation science standpoint, at this time, it is cell reprogramming iPSC-based cell therapies, in particular, that are predominant. While the notion of direct tissue reprogramming is gaining momentum with respect to scientific development, it does not represent a significant component of current products translated to clinical application. Bringing cell and tissue reprogramming therapies from development to market is a difficult task for biotechnology and pharmaceutical companies. Safety considerations including tumorigenicity, immunogenicity, genomic integration, off-target effects, dosing, induced cell functional phenotype, and cell culture heterogeneity and genetic variants must be monitored and minimized during the manufacturing process.^864,943,944^ iPSC industries, for example, are applying similar safety requirements adopted from monoclonal antibody production when creating, expanding, and purifying iPSC-derived cell lines. Closely monitored bioreactors and HLA-homozygous iPSC-derived cell banks (haplobanks) are being developed to scale up production and reduce market costs of cell therapies, while preclinical good laboratory practice studies, standardization, and vector elimination screenings are being conducted to reduce patient risks.^381,383,384,945^ While practices are not under universal regulatory standards, global regulatory bodies for cell-based and iPSC-based therapies exist in Australia (Therapeutic Goods Administration), European Union (European Commission of European Medicines Agency), Japan (Pharmaceuticals and Medical Devices Agency), United Kingdom (Medicines and Healthcare products Regulatory Agency), and United States (FDA) to impose standards and practices that must be met before these products are introduced in a clinical setting.^946^ The high costs currently associated with cellular reprogramming therapies raises ethical concerns that these life-saving treatments will be cost-restrictive, limiting their availability to prosperous populations within and between countries.^947^ Without development of safe industrial methods to scale-up production and reduce cost burdens, costs could worsen health disparities based on socioeconomic status.

Reprogramming cells and tissues by methods using iPSCs, viruses, and small molecules have associated toxicity risks that researchers are actively working toward minimizing or outright preventing. Nonviral gene delivery approaches such as, but not limited to, TNT are of outstanding interest. iPSC-based reprogramming methods carry the risks of producing off-target differentiation products or cells with proliferative oncogenic potential. Therefore, iPSC differentiation approaches must be optimized and screened to restrict differentiation pathways and prevent off-target cell products.^741^ Viral-based reprogramming methods carry the inherent risk of genomic insertions capable of inducing oncogenic events; however, use of nonviral, nonintegrating viruses, proper screening, cell-specific targeting, and optimization of reprogramming factor selection methods are being incorporated into cell and tissue reprogramming approaches to mitigate toxicity and health risks, while producing beneficial and promising therapeutic outcomes.^686^ Targeted electroporation and small molecules are emerging as promising avenues for nonviral reprogramming methods. The potential toxicity risk of using small molecules is derived from increased variability in cell-specific targeting.^326,659^ With optimization, more precise delivery methods, and better understanding of the molecular pathways, small molecule reprogramming represents a potentially clinically safe nonviral approach to produce therapeutically beneficial cell products with improved efficiency.^775^ First reports on targeted TNT pave the path to a future where cell subset—specific targeting would be evident minimizing off-target toxicity.^112,140,857,948^

Adapting new technologies for direct cell reprogramming is showing promise toward limiting off-target effects. CRISPRa, for example, combines attenuated gRNA and dCas9 with synthetic transcription activators improves site-directed targeting of endogenous gene loci. CRISPRa has been successful in reprogramming by activating silent genes in target cells including fibroblast to induced cardiac progenitor cells (GATA4, NKX2.5, and TBX5),^949^ fibroblast to iCMs (GATA4 and exogenous MEF2C and TBX5), myofibroblasts to iHeps (GATA4 and FOXA3).^672,677^ CRISPRa-mediated direct reprogramming is still in its infancy and carries safety concerns regarding off-target genomic editing, yet the method shows great potential for in vivo clinical applications in the future.

Incorporating direct reprogramming methods with other innovative technologies and devices provides new avenues for site-directed cell therapy delivery. Bone injuries, for example, can leave fracture nonunion where the healing process fails to complete.^950^ Rather than using traditional autologous bone grafting or iPSC-derived osteoblasts that bring a risk of teratoma formation, ex vivo direct reprogramming of patient derived fibroblasts to osteoblast-like cells can be combined with scaffolding material to be directly transplanted to the bone defect, promoting ossification.^622,868^ The use of scaffolding as a microenvironment for direct reprogramming can enhance reprogramming efficiency and maturation by inclusion of growth factors present in the native microenvironment of the target tissue and by mimicking the mechanical force of native ECM.^4,525,632,869^ New methods are currently being developed to induce in vivo localized direct reprogramming with biomaterial scaffolds. Scaffolding materials can be infused with reprogramming factors designed to induce direct reprogramming in a target tissue, leveraging existing cell types to promote regeneration.^622^ In a femoral osteotomy animal model, for example, animals were treated with an implantable collagen sponge scaffold infused with chemically modified BMP2 mRNA nonviral lipid vector.^951^ This scaffolding and localized expression of BMP2 created an osteogenic microenvironment that successfully promoted bone defect regeneration. Similar studies have seen promising reprogramming and regenerative results with factor-encapsulating scaffolds for bone (miR-26a),^952^ tendon (trichostatin A),^953^ cartilage (sodium diclofenac and bone marrow-derived MSC exosomes),^954^ nerve (polylactic acid and Wnt3a),^955,956^ angiogenesis (VEGF, BMP2, NT3, and miR222),^957–959^ cardiovascular (MSC secretome growth factors and exosomes),^960^ cardiac and smooth muscle (MYOD1),^961^ among others. While it is not yet possible to prevent these reprogramming scaffolds from affecting all off-target cells, these devices represent a step toward localized microenvironment regulation capable of promoting direct cell reprogramming and tissue regenerative outcomes.

## IX. Conclusion

Historically, cell types have been classified based on a variety of characteristics, including their morphology (shape and structure), functional attributes (such as physiological properties), specific location within tissues and organs, lineage or origin, and distinct cell-surface markers. This method of cell classification has played a crucial role in the advancement of biomedical sciences, providing a comprehensive understanding of tissue biology, facilitating the development of targeted therapies, improving the accuracy of disease diagnosis, and unraveling the cellular and molecular mechanisms that underpin health and disease. However, the emergence of advanced technologies has dramatically transformed our understanding of cell and tissue biology. These technologies have revealed that cells possess a far greater degree of functional dynamism and adaptability than was previously recognized. This insight has introduced the concept of “mosaic fluidity,” which suggests that cell types and functions are not rigidly fixed but can, instead, undergo changes in response to variations in their surrounding microenvironment and external cues. This realization challenges traditional notions of cell identity and function, emphasizing the fluid and responsive nature of cells within living tissues.

The field of in vivo tissue reprogramming has emerged not merely as an extension of developments in cellular reprogramming but as an entirely new discipline. Unlike in vitro cellular reprogramming, where the cellular environment can be tightly controlled, in vivo tissue reprogramming involves a highly complex and dynamic process in which the final state or fate of reprogrammed tissue is determined through active negotiation with a multitude of in vivo factors. These factors include the tissue microenvironment, immune system interactions, and circulating blood-borne elements, each playing a critical role in guiding the reprogramming process. As a result, the outcomes of in vivo tissue reprogramming are uniquely complex and may not always be fully reproduced in an in vitro laboratory setting, highlighting the distinct and sophisticated nature of this emerging field.

These fundamental conceptual advancements are redefining the landscape of medical drug therapies. The future of medical treatments is increasingly oriented toward precision medicine, regenerative therapies, and highly targeted interventions. In particular, the potential for medical drug therapies in regenerative medicine is remarkably promising. There is a growing emphasis on developing pharmaceutical agents capable of inducing cell and tissue reprogramming, with the aim of restoring or repairing tissues that have been damaged or diseased. The use of small molecules, biologic agents, and gene-editing tools offers the potential to target specific cellular pathways, thereby enhancing the body’s inherent ability to heal and regenerate. These emerging therapies are designed to activate the body’s natural regenerative capacity by engaging specific molecular pathways, aiming to restore tissue function that has been lost due to aging, injury, or disease processes. In doing so, they represent a transformative shift toward more personalized, effective, and adaptive treatment strategies.

## Figures and Tables

**Table 1:** In vivo nonviral direct reprogramming and functional outcome Outcome cell types listed do not necessarily qualify to be a fate change from the starting cell type. A fate-change call must be supported by evidence demonstrating that the outcome/product cell types have completely lost all characteristics (transcriptomic signature that would identify the product cell as a variant of the parent cell as determined by scRNA sequencing) of the parental cell while acquiring novel characteristics of the listed outcome/product cell. In the absence of such evidence, claimed transitions may be viewed as a form of state change of the parental cell, which may be transient until otherwise proven.

Target Site	Intended Reprogrammed Cell Type	Organ/Tissue Targeted	Method	Reprogramming Molecule/Factor	Morpholgy	Functionality	Marker	Disease State	Reference
Skin	Neuronal	Skin	Intradermal nanoparticle injection; electrical stimulation (triboelectrical stimulator)	Brn2, Ascl1, and Myt1	−	Induced neurogenic cells	TUJ1	Neuronal degeneration	^320^
Skin epidermis	Vasculogenic/neural	Skin	TNT, plasmid cutaneous	Vasculogenic TNT_EFF_; ABM (neurogenic TNT_ABM_)	+	Increased vascularization, rescue of necrotizing tissue; innervation, electrophysiologically active	PECAM-1, vWF; COL1A1, TUJ1, NF	Injury-induced hind limb ischemia	^321^
Oral gavage and intraperitoneal	Cardiomyocyte	Heart	Oral gavage (CRFTM); intraperitoneal injection (VP)	CHIR99021, RepSox, forskolin, VPA, Parnate; TTNPB, Rolipram	+	Action potential, improved ejection fraction and reduced fibrosis	*α*-actinin, cTN1, GATA4, MEF2C	Heart failure	^322^
Hippocampus	Neuron	Brain	Small molecule intracranial or intraperitoneal injection	CHIR99021, DAPT, SB431542, LDN193189	−	Hippocampus neurogenesis	DCX, KI-67, NEUN	Neuronal degeneration and injury	^323^
Peri-infarct myocardium	Cardiomyocyte	Heart	Gold nanoparticle injection	Gata4, Mef2c, Tbx5	+	Reduced fibrosis and infarcted area	TBX5, *α*-actinin, TNNT2, *α*-MHC	Myocardial infarction	^324^
Skin epidermis	Neurogenic	Skin	TNT, plasmid cutaneous	Ascl1, Brn2, and Myt1l (neurogenic TNT_ABM_)	+	Rescue pre-existing nerve fiber	NF200, NGF, NT3, PGP9.5, TUJ1	Diabetic peripheral neuropathy	^172^
Spleen	Hepatocyte progenitor cells	Liver	Small molecule intrasplenic injection	Y-27632, A-83–01, CHIR99021, HGF	+	Reduced fibrotic area, proliferate and promote regeneration	CD24, KI67, HNF4*α*, SOX9, CK19, CD24	Liver fibrosis	^325^
Striatum or cortex	Neuron	Brain	Small molecule osmotic minipump	Forskolin, CHIR99021, ISX9, I-BET151, Y-27632	+	Exhibited action potentials and synaptic connectivity	NEUN, DARPP32, NPY, PVALB, CTIP2, TBR1,	Neuronal degeneration and injury	^326^
Fibroblast, heart	Cardiomyocyte	Heart	Sequential targeting nanoparticle, tail vein injection	microRNA1, 133, 208, and 499	+	Attenuated fibrosis, improved ejection fraction	TNNT2, COL1, tdTomato-cardiac fibroblasts	Myocardial infarction, heart failure	^327^
Myocardium infarct junction	Cardiomyocyte	Heart	Nano dot-miRNA epicardium injection	miRNA-1, miRNA-133, miRNA-208, miRNA-499	−	Reduced fibrosis and scar area, improved ventricular wall thickness	CD31	Myocardial infarction	^328^
Liver tumor	Benign hepatocytes	Liver	Small molecule intratumor injection	SB431542, CHIR99021, BIX01294, all-trans retinoic acid	−	Reduced tumor burden, caused tumor fibrosis	AFP, HNF4*α*	Liver cancer	^329^
Eye, intravitreal	Neuron	Retina	Small molecule injection; inducible Tg mice	Trichostatin A; Islet1, Pou4f2, Ascl1	+	Mixed electrophysiologic activity	OTX2, ELAVl4, SOX11, SOX4, GAP43, SATB1, CNTN5, CALB2	Neuronal degenerative diseases	^330^
Skin and tibialis anterior	Myogenic cells	Skeletal muscle	TNT, plasmid nanoporation	MyoD (myogenic TNT_MyoD_)	+	Recover volumetric muscle loss, improved muscle torque	MF20, MGN, MYHC7, MRF4, MYF5	Volumetric muscle loss	^331^
Intraperitoneal	Neuron	Brain	Small molecule injection	CHIR99021, Dorsomorphin, forskolin, ISX9, Y-27632, DB2313		Improved motor sensory function	MAP2, TUJ1, NEUN, NEUROD1	Ischemic stroke	^332^
Eye, intravitreal	Neuron	Retina	Small molecule injection	DMX-5804	−	Neuronal cells	PAX6, NEUN, MAP2, Calretinin 9, LATS, YAP, SOX9	Retina degenerative diseases	^333^
Ipsilateral striatum	Neuron	Brain	Plasmid injection	phVEGF	−	Reduced cerebral infarction volume	p-ERK, PAX6	Stroke	^334^
Intraperitoneal	Neuronal cells	Spinal cord	Small molecule injection	Ginsenoside Rg1	−	Locomotive recovery, reduced lesion cavity	MAP2, NEUN, HES1, HEY1	SCI	^335^
Oral gavage	Neuron	Intestine	Small molecule	RepSox	−	Neuronal cells, increased gastrointestinal motility	HuCD, SOX10, GFAP, S100*β*	Gastrointestinal neuropathy	^336^
Eye, intraocular	Muller glial–derived progenitor cells	Retina	Small molecule injection	BMS309403, FGF2, C75, G28UCM		Proliferation of MG progenitor cells	PCNA, CDK1, TOP2A, ASCL1	Retinal injury	^337^
Heart, tamoxifen induced	Cardiomyocyte	Heart	Inducible Tg mice	Mef2c, Gata4, Tbx5, Hand2	+	Reversed fibrosis, improved left ventricle ejection fraction and end-systolic/stroke volumes, improved survival	TNNT2, HF, NPPA, NPPB, *α*-actinin, CX43	Myocardial infarction	^338^
Intraperitoneal	Hepatocyte (regenerative)	Liver	Small molecule intraperitoneal injection	Salidroside, CID755673, forskolin, GSK429286A, ETC-1002	+	Rescue liver function (reduced bilirubin and aspartate aminotransferase), regeneration, reduced fibrosis	HNF4a, CYP3A4, CYP3A13, CYP2B10, TTR	Prolonged liver injury	^339^
Motor cortex	Neuron	Brain	Motor cortex: small molecule osmotic minipump; hydrogel transplant	LIF, vitamin C, *β*-ME, BPTES, 2-DG; hydogel with chitosan nanoparticle (insulin/bFGF/transferrin-conjugated)	+	Improved motor coordination and function	GFAP, NEUN, TUJ1	Motor cortex injury	^340^
Subretinal cell	ONL (outer nuclear layer) cells	Retina	Conditional expression plasmid injection	Ikzf1, Ikzf4	+	ONL cell migration to apical side of retina	RXRG, LHX2, VSX2, FOSN3, NEUROD4	Retinal injury and degenerative diseases	^341^
Retinal cells	Neuron	Retina	Electroporation	NGN1/3	+	RGCs	RBPMS, NF200	Glaucoma	^342^
Skin	Vasculogenic fibroblast	Skin	TNT, plasmid nanoporation	Anti-miR200b oligonucleotide (ASO) (vasculogenic TNT_ASO_)	+	Improved perfusion, vasculature and wound closure	COL1A2, CD31, FSP1, vWF, FLI1	Ischemic wounds	^112^
Intradermal cells	Dermal papilla	Skin	Hydrogel microspheres—nanoparticle injection	Tideglusib and tamibarotene	+	Reduced wound fibrosis, promoted hair follicle regeneration	KRT14, TGF*β*R2	Skin wounds	^343^
Intraperitoneal	Neuronal cells	Spinal cord	Small molecule injection	LDN193189, CHIR99021	+	INDUCED neurogenesis in spinal cord	DCX, TUJ1, MAP2, NEUN, SYN1, GABA, GAD6	SCI	^344^
Eye, intravitreal	Neuron	Retina	Small molecule injections	Metformin or DBZ; Ascl1 Tg mice	+	Promoted glia-derived neurogenesis	OTX2, HuC/D, MG-lineage GFP, sci-Plex	Retina degenerative diseases	^345^
Intraperitoneal	AEC-I	Lung	Small molecule injection	CHIR99021		Reduced apoptosis, rescue of alveolar epithelial population	PDPN, proSP-C	Acute respiratory distress syndrome	^346^
Skeletal muscle	Dedifferentiated muscle (innervation permissive)	Muscle	Dox slow-release Elvax sheets under hindlimb skin	NANOG Dox-inducible Tg mice		NMJ formation, muscle innervation	PAX7, MYHC, PAX7, NRG1, NRG2, NGF	Peripheral nerve injury	^347^
Oral gavage	Pancreatic *β*	Pancreas	Small molecule	PF-562271		Improve glucose homeostasis, insulin secretion	GLUT2, PDX1, NKX6.1	Diabetes	^348^
Dermis	Myoepithelial cells	Skin	Proteinaceous nanoformulation injection	VPA, SB431542, retinoic acid, tranylcypromine, cyclic pifthrin-*α*, BMP4, EGF	+	Accelerated wound healing, reduced fibrosis, sweat gland regeneration	*α*-SMA, K10, K18, AQP5	Skin wounds	^349^
Inner ear scala media	Cochlear hair cell	Ear	Small molecule microinjection	PD0325901	+	Potassium currents, hair cell regeneration	MYO7A, SOX9	Hearing loss	^350^
Skin epidermis	Vasculogenic	Skin	TNT, plasmid cutaneous	Vasculogenic TNT_EFF_		Rescued perfusion and wound closure	COL1A2, VWF, PECAM1, TET1/2/3, lectin, CDH5,	Ischemic limb wounds	^140^
Myocardial infarction margin area	Cardiomyocyte	Heart	EV injection	Cardiomyocyte EVs	+	Inhibited myocardial cell apoptosis, improved blood supply, improved ventricular wall thickness	cTNT, *α*-actinin, RYR2, NKX-2.5, ISL-1, MESP-1, GATA4, FLK-1, CX43	Myocardial infarction	^351^
Intraperitoneal and oral gavage	Cardiomyocyte	Heart	Small molecule cocktail	CHIR99021, RepSox, forskolin, VPA, TTNPB, Rolipram, Parnate	+	Cardiomyocyte cells (lineage tracing from cardiac fibroblasts)	*α*-Actinin, cTnl, TBX5, MYL2, TTN, MYBPC3, PLN, RYR2	Myocardial infarction	^352^

**Table 2:** Small molecule reprogramming factors Growth factors included fibroblast growth factors (FGF, bFGF, and FGF2); epidermal growth factor; hepatocyte growth factor (HGF); TGF; and nerve growth factor (NGF).

Small Molecules	Function	Cocktails	Disease State	Functional Outcome/Rescue	Reference
1,4-DPCA	Inhibitor of hypoxia-inducible transcription factor prolyl 4-hydroxylase	1,4-DPCA, FGF2, BMP4, mLif, A8–301, CHIR99021	Absolute uterine factor infertility	Uterine glandular epithelium	^457^
mLif	Monocyte locomotion inhibitory factor				
20*α*-Hydroxycholesterol	Hedgehog pathway activator	20*α*-Hydroxycholesterol, forskolin, luteinizing hormone, SB431542	Male hypogonadism	Androgen synthesis in testes in vivo	^458^
		20*α*-Hydroxycholesterol, forskolin, DAPT, purmorphamine, 8-Br-cAMP, SAG	Male hypogonadism	Expressed testosterone-synthesizing enzymes in vitro; promoted testosterone recovery in vivo	^459^
2-deoxy-d-glucose (DG)	Glucose analog; glycolysis inhibitor	2-DG, BPTES, LIF, vitamin C, *β*-mercaptoethanol (ME); hydogel with chitosan nanoparticle	Motor cortex injury	Improved motor coordination and function in vivo	^340^
BPTES (bis-2-(5-phenylacetamido - 1,3,4-thiadiazol-2-yl)ethyl sulfide)	Glutaminase inhibitor				
*β*-ME	Neuron survival in culture				
5-AZA	DNMT inhibitor	5-AZA, miR-133	Cardiovascular disease	Epigenetic-enhanced reprogramming efficiency	^460^
8-Br-cAMP	Activates cAMP-dependent protein kinase; PKA activator	8-Br-cAMP, VPA, CHIR99021, RepSox, forskolin, Y-27632, BMP4	Ischemic diseases	Generated vascular structures in vivo	^461^
		8-Br-cAMP, forskolin, DAPT, purmorphamine, 20*α*-hydroxycholesterol, SAG	Male hypogonadism	Expressed testosterone-synthesizing enzymes in vitro; promoted testosterone recovery in vivo	^459^
A83-01	TGF-*β* antagonist	A83-01,CHIR99021, LDN193189, RG108, dorsomorphin, P7C3-A20, ISX9, forskolin, Y27632, DAPT, PD0325901, purmorphamine, P7C3-A20	Alzheimer disease, PD	Misfolded protein accumulation; mitochondrial abnormalities	^462^
		A83-01, CHIR99021, HGF	Hepatitis, cirrhosis, fatty liver disease	Hepatic progenitor cells and organoids	^463–465^
		A83-01, CHIR99021 and a 3D scaffold	Cholangiopathy	Hepatic progenitors; restored bile flow in vivo	^466^
		A83–01, Y-27632, CHIR99021	Liver injury and cirrhosis	Hepatic progenitor cells; survived in vivo without tumorigenesis	^467^
		A83–01, Y-27632, CHIR99021, HGF	Liver fibrosis	Reduced fibrotic area; proliferated and promoted regeneration in vivo	^325^
		miR-1 and miR-133a, Gata4, Mef2c, Tbx5, Myocd, Nkx2-5; SB432542, CHIR99021	Heart disease and injury	Exhibited voltage-gated Ca^2+^ channels, adrenergic receptors	^468^
Activin A	TGF-*β* family; inhibits osteoblast differentiation	Activin A, CHIR99021, EPZ5676, B27, *β*-ME, RG108, P8511, LYZ294002, EPZ011989,	Intestinal disease model	Intestinal barrier function	^469^
EPZ5676; EPZ011989	Histone methyltransferase inhibitor	Noggin, SB431542, BMP4, retinoic acid			
LYZ294002	Pan-PI3K inhibitor				
AM580	Retinoic acid receptor-*α* activator	AM580, CHIR99021, E-616452, forskolin, EPZ004777, CH55, UNC0638	Liver failure	Drug metabolism in vitro; liver repopulation in vivo	^470^
CH55	Retinoic acid receptor activator				
UNC0638	Euchromatic histone methyltransferase substrate inhibitor				
AS8351	Histone KDM5B demethylase inhibitor	AS8351, BIX01294, CHIR99021, A83-01, SC-1, Y-27632, OAC2, SU16F, JNJ10198409	Myocardial infarction	contractility, beating cardiomyocytes in vitro, remuscularized infarcted area in vivo	^471^
Y-27632	Rho kinase (ROCK)-1/2 inhibitor				
JNJ10198409	Platelet-derived growth factor receptor (PDGFR) inhibitor				
OAC2	Oct4 activator				
Baricitinib	Janus kinase (JAK) inhibitor	Baricitinib, SB431542; Mef2c, Tbx5	Injured cardiac tissue	calcium transients, beating cardiomyocytes, action potentials	^472^
BIX01294	Histone methyltransferase inhibitor	BIX01294, SB431542, LDN193189, CHIR99021, DAPT	Liver diseases	Lipid accumulation, ALB secretion in vitro, promote liver regeneration in vivo	^473^
		BIX01294, SB431542, CHIR99021, all-trans retinoic acid	Liver cancer	Reduced malignant metabolic profile in vivo	^474^
Blebbistatin	Nonmuscle myosin II inhibitor	Blebbistatin	Neuronal degeneration model	misfolded protein accumulation, mitochondrial abnormalities	^475^
BMP4	Retinoic acid receptor-*α* activator; growth factor	BMP4, RepSox, CHIR99021, isoproterenol, retinoic acid; ectodermal dysplasia antigen (EDA)	Skin wounds	Ach-dependent calcium transients, sweat gland regeneration in vivo	^476^
Isoproterenol	*β*2-AR activator				
BMS309403	Fatty acid–binding protein 4 inhibitor	BMS309403, FGF2, C75, G28UCM	Retinal injury	Proliferation of MG progenitor cells in vivo	^337^
C75	Fatty acid synthase inhibitor				
G28UCM	Fatty acid synthase inhibitor				
Celecoxib	Cyclooxygenase 2 inhibitor	Celecoxib, VPA, CHIR98014, RepSox, TTNPB	Articular cartilage injury and degeneration	Cartilage matrix formation and rescue of mechanical function in vivo	^477^
CHIR99021	GSK3 inhibitor (activates Wnt signaling)	CHIR99021	Acute respiratory distress syndrome	Reduced apoptosis, rescue of alveolar epithelial population in vivo	^346^
		CHIR99021, RepSox, forskolin, VPA	Heart failure	Beating cardiomyocytes	^478^
		Forskolin, ISX9, CHIR99021 I-BET 151, DAPT	Glioblastoma multiforme	Lost tumor spheroid formation	^479^
		CHIR99021, DAPT, LDN193189, SB431542	Neuronal degeneration and damage	Neurogenic cells	^480^
		VPA, CHIR99021, forskolin, 1,9 pyrazoloanthrone (SP600125), Y-27632, melatonin	PD, AD, and Huntington disease	Neural cells, autophagy activation	^481^
		miR-2392; forskolin, VPA, CHIR99021	Ischemic heart disease, myocardial infarction	Cardiomyocytes	^482^
		VPA, CHIR99021, RepSox	Neuronal degeneration	Neural progenitor cells, behavioral function rescue in vivo	^483^
		CHIR99021, DAPT, SB431542, LDN193189	Neuronal degeneration and injury	Hippocampus neurogenesis in vivo	^323^
CID755673	Protein kinase D inhibitor	CID755673, salidroside, forskolin, GSK429286A, ETC-1002	Prolonged liver injury	Rescued liver function (reduced bilirubin and aspartate aminotransferase); regeneration; reduced fibrosis in vivo	^339^
ETC-1002	AMPK activator				
GSK429286A	ROCK inhibitor				
Salidroside	Phenylpropanoid glycoside				
Ciglitazone	Peroxisome proliferator-activated receptor (PPAR)γ agonist; promotes adipocyte differentiation	Ciglitazone	Fatty liver disease	Lipid accumulation	^484^
CNTF	Ciliary-derived neurotrophic factor	CNTF, BMP4, CHIR99021, SB431542, A83-01, forskolin, dbcAMP, LIF	Late-onset neurologic disorders	Functional astrocytes in vitro	^485^
CTPB	P300 histone acetyl transferase activator	CHIR99021, A83-01, GSK126, forskolin, CTPB, AM580	Myocardial infarction	Cardiovascular progenitor cells; multilineage cardiovascular differentiation in vivo	^486^
GSK126	Histone methyltransferase inhibitor				
Curcumin	Turmeric; antioxidant; anti-inflammatory	Triiodothyronine and curcumin	Neurologic diseases	Neural precursor cells	^487^
Triiodothyronine	Thyroid hormone; reprogramming enhancer (c-Myc–like function)				
Cyclic pifthrin-*α*	p53 inhibitor	VPA, SB431542, retinoic acid, tranylcypromine, cyclic pifthrin-*α*, BMP4, EGF	Skin wounds	Accelerated wound healing, reduced fibrosis in vivo	^349^
DAPT	NOTCH1 inhibitor	Forskolin, DAPT, purmorphamine; Sf1	Hypogonadism	Testosterone secretion	^488^
		DAPT (or short-hairpin [sh]RNA NOTCH1)	Neuronal degeneration and injury	Synapse formation in vivo	^489^
DB2313	Transcription factor PU.1 inhibitor	CHIR99021, Dorsomorphin, forskolin, isoxazole-9 (ISX-9), Y27632, DB2313	Ischemic stroke	Improved motor sensory function in vivo	^332^
dbcAMP	Dibutyryl cyclic-AMP; activates cAMP-dependent protein kinases	VPA, bFGF, dbcAMP; miR-9/9*, miR-124, NeuroD2 ± Ascl1 and Mtyl1	Neuronal degeneration	Exhibited action potentials; synapse formation	^490^
		dbcAMP, forskolin, ISX9, CHIR99021, I-BET151, Y-27632	Retinal degenerative diseases	Exhibited action potentials	^491^
DBZ	γ-Secretase/Notch inhibitor	Metformin, DBZ; Ascl1 Tg mice	Retinal degenerative diseases	promoted neurogenesis in vivo	^345^
Metformin	LKB1/pAMPK activator				
DMH1	Inhibitor of BMP	CHIR99021, DMH1, RepSox, forskolin, Y 27632, SP600125	Neurologic disease	Neuronal cells	^492^
DMX-5804	MAP4K4/6/7 inhibitor	DMX-5804	Retinal degenerative diseases	Neuronal cells	^333^
Dorsomorphin	TGF-*β* inhibitor	Dorsomorphin, trichostatin A	ALS, PD	Neuroectodermal cells	^493^
		CHIR99021, VPA, dorsomorphin, SB431542, forskolin	Cardiovascular disease	Spontaneous calcium oscillation	^494^
DZNep	Histone methyltransferase inhibitor	CHIR99021, RepSox, VPA, Parnate, TTNPB; Dznep + 1 TF (Foxa3, Hnf1*α*, or Hnf4*α*)	Liver failure, liver metabolic disorders	Glycogen accumulation, low-density lipoprotein (LDL) absorption, cytoplasmic accumulation of neutral triglycerides and lipids, albumin secretion	^495^
EPZ004777 (C6F5UE)	Histone methyltransferase inhibitor	5-AZA, CHIR99021, SB431542, forskolin, VPA, EPZ004777	Corneal disorders	Self-renewal capacity, reverse corneal opacity in vivo	^496^
Forskolin	Adenylyl cyclase (cAMP) activator	Forskolin, VPA, RepSox, tranylcypromine, TTNPB	Epithelial lactation mechanisms	Mammary epithelial cells	^497^
		forskolin, VPA, RepSox, tranylcypromine, TTNPB	Mammary gland defects	Regenerate mammary gland structure in vivo	^498^
		Forskolin + neuronal medium (neurobasal, cAMP, bFGF)	Neuronal degeneration	Exhibited action potentials in vitro, survival >2 mo in vivo	^499^
		Forskolin, VPA; NeuroG2, Cend1	Neuronal degeneration and injury	Neural precursor cells	^500^
Ginsenoside Rg1	Neuroprotective herb; block Notch/Stat3 pathway	Ginsenoside Rg1	SCI	Locomotive recovery in vivo	^335^
GO6983	PKC inhibitor	GO6983, VPA, CHIR99021, RepSox, forskolin, SP600125, Y-27632	AD	Exhibited action potential; synapses	^501^
Heregulin-B1-EGF domain	Neuregulin family growth factor	bFGF, forskolin, PDGF, heregulin-B1-EGF domain	Multiple sclerosis	Axon regeneration, NMJ rescue in vivo	^502^
Hh-Ag1.5	Hedgehog smoothened receptor agonist	A83-01, CHIR99021, LDN193189, Hh-Ag1.5, retinoic acid, SMER28, RG108, Parnate, bFGF	Demyelinating disease	Myelinating activity	^503^
I-BET151	Extra-terminal domain and bromodomain protein inhibitor	CHIR99021, RepSox, forskolin, i-BET151, ISX-9	Neuronal degeneration and injury	Exhibited action potentials and synapse formation in vivo	^504^
IBMX	Phosphodiesterase inhibitor	miR-9, 124, 155, and 224, separately; IBMX, NGF, retinoic acid	Neuronal degeneration and injury	Exhibited calcium excitability	^505^
ISX-9	Isoxazole 9; Wnt/*β*-catenin activator; neurogenesis promoter	Forskolin, CHIR99021, ISX9, I-BET151, Y27632	Neuronal degeneration and injury	Exhibited action potentials and synaptic connectivity in vivo	^326^
IWR1	Wnt/*β*-catenin pathway inhibitor	VPA, CHIR99021, RepSox, forskolin, IWR1	Retinal degenerative diseases	RCVRN^+^ photoreceptor cells that survived in vivo	^506^
JQ-1(+)	Bromodomain inhibitor	JQ-1(+), CHIR99021, RepSox, forskolin, Y276332, trichostatin A	Neuronal degeneration and injury	Spontaneous calcium influx and outflow	^507^
Kenpaullone	ALK5 inhibitor	Kenpaullone; Ngn2, Sox11, Isl1, Lhx3	ALS	Exhibited action potential, synapse formation	^508^
LDN193189	TGF-*β* inhibitor; BMP-receptor inhibitor	LDN193189, CHIR99021	SCI	Injury-specific iNs in vivo	^344^
LiCl	GSK3 and LSD1 inhibitor	LiCl, VPA, forskolin; siRNA Fir, siRNA Mxi1	Hearing loss (cochlear hair cells)	Hair cell regeneration in vivo	^509^
mLif	Monocyte locomotion inhibitory factor	FGF2, BMP4, mLif, 1,4-DPCA, A8–301, CHIR99021	Absolute uterine factor infertility	In vitro model	^457^
NaB	Sodium butyrate, HDAC inhibitor	A83-01, CHIR99021, Y27632, TTNPB, forskolin, VPA, NaB	Neuronal degeneration	Partial electrophysiological activity	^510^
Noggin	BMP antagonist	SB431542, Noggin, retinoic acid	PD	Dopamine secretion, synapses, calcium influx	^511^
P7C3-A20	Neuroprotective and proneurogenic activity	A83-01, CHIR99021, LDN193189, RG108, dorsomorphin, P7C3-A20, ISX9, forskolin, Y27632, DAPT, PD0325901, purmorphamine	Neurologic disorders	Exhibited action potentials, synapses, and local circuit integration in vivo	^512^
Parnate (Tranylcypromine; P8511)	Lysine demethylase 1 inhibitor	TTNPB, forskolin, RepSox, tranylcypromine, VPA	Epithelial lactation mechanisms	Mammary epithelial cells	^513,514^
PD0325901	MEK/ERK inhibitor	PD0325901	Hearing loss (cochlear hair cells)	Exhibited action potentials; promoted hair cell regeneration in vivo	^350^
PF-562271	Focal adhesion kinase inhibitor	PF-562271	Diabetes	Improved glucose homeostasis; insulin secretion in vivo	^348^
Phenamil	Peroxisome proliferator-activated receptor (PPAR)γ activator	RepSox, forskolin, phenamil	Osteoporosis, bone injury	Bone matrix formation in vivo	^515^
Phorbol 12-myristate 13-acetate (PMA)	PKC activator	CHIR99021, A83-01, tranylcypromine, forskolin, VPA, PMA	Skin wounds	Improved wound healing; restored functional sweat glands	^516^
Purmorphamine	Activates hedgehog signaling pathway	Kenpaullone, forskolin, purmorphamine, Y-27632, retinoic acid	ALS	Exhibited action potentials	^517^
QVD-OPH	Caspase inhibitor	QVD-OPH, VPA, CHIR99021, RepSox, forskolin, SP600625, GO6983, Y27632, ISX9, I-BET151, retinoic acid, vitamin C	PD, AD, traumatic brain injury	Exhibit voltage-gated Na^+^ and K^+^ currents	^518^
SP600625	JNK pathway inhibitor				
RepSox	(E-616452) ALK5 inhibitor	RepSox	Gastrointestinal neuropathy	Exhibited action potentials; increased gastrointestinal motility in vivo	^336^
Retinoic acid	Wnt signaling regulator	SB431542, CHIR99021, BIX01294, all-trans retinoic acid	Liver cancer	Reduced malignant metabolic profile in vivo	^329^
RG108	DNA methyltransferase inhibitor	Bix01294, RG108, VPA, PD0325901	Thrombocytopenia	Fibrin clot and platelet plug formation in vitro; platelet production in vivo	^519^
Riluzole	Glutamate blocker; ALS treatment	I-BET151, retinoic acid, riluzole	Sertoli cell infertility defects	Engulfment of apoptotic sperms in vitro; formation of testicular seminiferous tubules in vivo	^520^
Rolipram	Phosphodiesterase-4 inhibitor	CHIR99021, RepSox, forskolin, VPA, Parnate, TTNPB, Rolipram	Heart failure	Exhibited action potentials; improved ejection fraction; and reduced fibrosis in vivo	^322^
Ruxolitinib	Tyrosine kinase inhibitor	Y26732, DAPT, RepSox, CHIR99021, ruxolitinib, SAG	AD, PD	Exhibited action potentials	^521^
SAG	Smoothened agonist; hedgehog pathway activator	LDN193189, SB431542, TTNPB, thiazovivin, CHIR99021, VPA, DAPT, SAG, purmorphamine	Neuronal degeneration and injury	Exhibited action potentials in vitro; integrated into brain neuron circuits; and formed synapses in vivo	^522^
SB203580	MAPK/p38 inhibitor	Kenpaullone, forskolin, SB431542, SB203580	Skeletal muscle degenerative diseases	Contractility, myotube formation	^523^
SB431542	TGF-*β* inhibitor	FUW-tetO-pMyoD1; FGF2, SB431542, CHIR99021, forskolin	Skeletal muscle diseases	Formed striated sarcomere structure	^524^
SC-1	Extracellular signal–regulated kinase 1 inhibitor; Ras guanosine triphosphatase inhibitor	A83-01, CHIR99021, forskolin, SC-1; 3D heart ECM	Myocardial infarction	Beating cardiomyocytes, calcium transients in vitro; improved cardiac function in vivo	^525^
SMER28	Small molecule enhancer of autophagy	CHIR99021, LDN193189, A83-01, Hh-Ag1.5, retinoic acid, SMER28, RG108, Parnate, bFGF	Demyelinating disease	Myelinating activity	^503^
Sodium butyrate	Histone deacetylase inhibitor	Gata4, Mef2c, Tbx5, Hand2, Myocd, miR590; sodium butyrate, ICG-001, retinoic acid	Heart failure	Spontaneous cardiomyocyte beating; contractility	^526^
SP600125	JNK pathway inhibitor	Forskolin, RepSox, SP600125, CHIR99021, Go6983, Y-27632, IXS9 and I BET151	AD, Huntington disease, epilepsy	Membrane current properties	^527^
STK287794	Stimulates lipid droplet deposits in HDFs	STK287794	Fill voids following surgical resection (eg, tumor removal)	Lipid accumulation and formation of granulation tissue containing white and brown adipocytes in vivo	^171^
Tamibarotene	Retinoid receptor agonist	Tideglusib and tamibarotene	Skin wounds	Reduced wound fibrosis; promoted hair follicle regeneration in vivo	^343^
Tideglusib	GSK3*β* inhibitor				
Taurine	Inhibits metabolic reprogramming and dysfunction	VPA, CHIR, RepSox, forskolin, IWR1, Sonic hedgehog, taurine, retinoic acid	Retinal degenerative diseases	Restoration of the pupil reflex and visual function in vivo	^528^
Sonic hedgehog	CNS organization, cell proliferation, and differentiation				
Thiazovivin	ROCK inhibitor	CHIR99021, forskolin, RepSox, LDN, VPA, thiazovivin	Multiple sclerosis	Differentiation to mature oligodendrocytes in vivo	^529^
Trichostatin A	Histone deacetylase inhibitor	Trichostatin A; Islet1, Pou4f2, Ascl1	Neuronal degenerative diseases	Mixed electrophysiological activity in vivo	^330^
TTNPB	RAR activator	VPA, TTNPB, RepSox	Obesity, lipid disorders	Lipid accumulation	^530^
		TTNPB	Hair loss	Hair follicle formation in vivo	^531^
VPA	HDAC inhibitor	VPA, forskolin, Y-27632; NeuroD1	Neuronal injury	Neuronal cells	^532^
		Sox2 and Pax6 cmRNA; vaporic acid and RepSox	Neuronal degeneration disease model	Neural precursor cells	^533^
		miR-302/367; VPA	Neuronal degeneration and injury	Sodium channel mediated repetitive spike firing in vitro; GFP-neuroblast detection in vivo	^534^
Vitamin C	H3K36 demethylase cofactor; antioxidant; *α*-ketoglutarate dependent dioxygenases activator	Vitamin C; GMT	Myocardial infarction	Improved GMT reprogramming efficiency; reduced ROS production	^535^
Xav939	Tankyrase inhibitor (Wnt pathway)	Myocd, Smad6, Tbx20; Xav939	Heart disease	Exhibited cardiomyocyte contractility; calcium transients	^536^

**Table 3:** In vitro/ex vivo direct reprogramming not involving in vivo applications Outcome cell types listed do not necessarily qualify to be a fate change from the starting cell type. A fate-change cael must be supported by evidence demonstrating that the outcome/product cell types have completely lost all characteristics (transcriptomic signature that would identify the product cell as a variant of the parent cell as determined by scRNA sequencing) of the parental cell while acquiring novel characteristics of the listed outcome/product cell. In the absence of such evidence, claimed transitions may be viewed as a form of state change of the parental cell, which may be transient until otherwise proven.

Starting Cell Type	Intended Reprogrammed Cell Type	Organ/tissue	Method	Reprogramming Molecule/Factor	Morphology	Functionality	Marker	Disease State/Model	Reference
Fibroblast, foreskin	Neurons	Neuronal	Lentivirus transduction	miR-9/9*, miR-124, NeuroD2 +/− Ascl1 and Mtyl1; medium w/VPA, bFGF, dbcAMP	+	Exhibited action potentials, synapses	SCN1a, BAF53b, BAF45b, BAF45c, MAP2, TUJ1, SYN1, BSN, PCLO, SHANK3	Neuronal degeneration	^490^
Fibroblast, heart	Cardiomyocyte	Heart	miRNA transient transfection	miRNA 1, 133, 208, and 499; JAK inhibitor I	−	l-type channel expression, spontaneous calcium oscillations and contractility	TNNI3, *α*MHC, CFP, *α*-actinin	Cardiac injury	^629^
Astrocytes	Neuroblast	Brain	Lentivirus transduction	miR-302/367 GFP-tagged	−	Sodium channel–mediated repetitive spike firing	DCX, TUJ1, NeuN	Neuronal degeneration and injury	^534^
Fibroblast, foreskin	Neuronal	Neuronal	Small molecule cocktail	VPA, CHIR99021, RepSox, forskolin, SP600125, GO6983, Y-27632	+	Exhibited action potentials, synapses	NCAM1, DCX, MAP2, ASCL1, NEFL	AD	^501^
Fibroblast, MEF	Cardiomyocytes	Heart	Small molecule cocktail	CHIR99021, RepSox, forskolin, VPA	+	Beating cardiomyocyte cells	*α*-Actinin, TNNT2, cTN1, MYH6, MEF2C, GATA4, NKX2	Heart failure	^478^
Fibroblast, MEF	Neurogenic	Skin	Nanochannel electroporation (NEP)	Ascl1, Brn2, and Myt11 (neurogenic NEP_ABM_)	+	Neurogenic fibroblast	TUJ1, CCNA2, NESTIN	Neuronal degeneration	^630^
Fibroblast, MEF	Neuronal	Skin	NEP (triboelectrical stimulator)	Brn2, Ascl1, and Myt1	−	Functional ligand–gated channels, action potentials	TUJ1, MAP2, Nestin, NEUN, vGLUT1	Neuronal degeneration	^320^
Fibroblast, MEF	Neurons	Brain	Small molecule cocktail	SB431542, Noggin, retinoic acid	+	Dopamine secretion, synapses, calcium influx	Synapsin, TUJ1, Nestin, MAP2, SOX1, NEUN, GFAP	PD	^511^
Fibroblast, skin	Neurons	Spine (CNS)	Lentivirus transduction; small molecule	Ngn2, Sox11, Isl1, Lhx3; kenpaullone	+	Exhibit action potential; form synapses	HB9, CHAT, VACHT, TUJ1, MAP2	ALS	^508^
Fibroblast, heart	Cardiomyocyte	Heart	Lentivirus transduction	Gata4, Mef2c, Tbx5, miR-590	−	Beating cardiomyocyte cells	*α*-Sarcomeric actinin, TNNT2, MYH6, TNNC1, NPPA, RYR2	Heart disease	^631^
Fibroblast, heart and tail-tip	Cardiomyocyte	Heart	miRNA transfection; 3D hydrogel	Pre-miR miRNA precursors for miR-1, miR-133, miR-208, miR-499	+	Enhanced reprogramming to cardiomyocyte	*α*-Sarcomeric actinin, TNNT2, KCNJ2, *α*MHC, MEF2C, TBX5, HAND2, MMP	Myocardial infarction	^632^
Pancreatic exocrine cells	Insulin-producing cells	Pancreas	Synthetic mRNA transfection	Pdx1, Neurogenin3, MafA	−	Insulin and C-peptide expression	GLUT2, KIR6.2, PCSK1, PCSK2, PAX4, NKX2.2, SUR1, GLP1r	Diabetes (type 1)	^633^
Endothelial progenitors	Smooth muscle cells	Muscle	Lentivirus transduction	Myocd	+	Exhibited contractility	ACTA2, MYH11, TAGLN	Vascular disease	^634^
Fibroblast, heart	Cardiomyocyte	Heart	Adenovirus or lentivirus injection	Gata4, Mef2c, Tbx5	−	Cardiomyocyte cells	TNNT2, *α*-actinin	Myocardial infarction	^635^
Astrocytes	Neuron	Brain	Lentivirus transduction	NeuroD1, Ascl1, Lmx1, miR218; ascorbic acid, SB431542, LDN193189, TGF*β*	+	Voltage-gated currents, stable morphology, and gene expression	MAP2, TUJ1, TH, HES6, GAP43, KIF1A, CXCR4, NEUN, SYN1, KCNJ6, DDC, PBX1, SLC6A3	PD	^636^
Fibroblast, skin	Myoblasts and myotubes	Muscle	AAV transduction	MyoD	+	Contractility, calcium release	Dysferlin, MYOD, CKM, myogenin, MHC, dystrophin, *β*-actin	Muscular disorders (eg, DMD)	^637^
RPE	Neuronal	Retina and neuronal	AAV transduction	miR-183, 96, 182	+	Neuronal cells	OTX2, NRL, PDC, DCT, Rhodopsin, CRX, Thy1	Retinal degeneration (eg, macular degeneration)	^638^
Fibroblast, skin	Neurons	Spine (CNS)	Lentivirus transduction	Isl1, Lhx3, Bcl-9/9*-142	+	Exhibited action potentials, synapses	MAP2, TUBB3, NEUN, NCAM, SCN1A, SNAP25, NRCAM, NEFM	ALS; SMA	^639^
Fibroblast, skin	Cardiomyocytes	Heart	Lentivirus transduction; small molecule	miR-1 and miR-133a, Gata4, Mef2c, Tbx5, Myocd, Nkx2-5; SB432542, CHIR99021	+	Voltage-gated Ca^2+^ channels; adrenergic receptors	ACTN2, TNNT2, VIM, MYH6/7, TAGLN, SMA, MYH11	Heart disease and injury	^468^
Fibroblast, human	Neuron	Neuronal	Lentivirus transduction	Ascl1, Isl1, NeuroD1, Brn2, Hb9, Lhx3, Myt1L, Ngn2	+	Neurite outgrowth, migration, neuronal connections	TUJ1, ISL1, CHAT, HB9	SMA	^640^
Fibroblast, MEF	Neural cells	Neural	Nanoparticle electrophoresis	Ascl1, Brn4, Tcf3	+	Neural cells	GFAP, Nestin, NF200, TUJ1, MAP2, GAP43	Neural system injury	^641^
Fibroblast, human	Multiple lineages: neurogenic, adipogenic, hepatogenic		HVJ-E transduction	Oct4, C/EBPb, C/EBP*α*; or Gata3, NeuroD1; or Hnf1a, FoxA2: Oct4, C/EBP*α* (adipogenic); Gata3, NeuroD1 (neurogenic); Oct4, Gata3, Hnf1a, FoxA2 (hepatogenic)	+	Adipocytes, neurocytes, hepatocytes	C/EBP*α*, PPARγ, lipid droplets (adipogenic); Nestin, MAP2 (neurogenic); TDO, E-cadherin, albumin (hepatogenic)	Multiple lineage cell products for regenerative therapies	^628^
Fibroblast, fetal lung (MRC5)	Hepatocytes	Liver	Lentivirus transduction	Atf5, Prox1, FoxA2, FoxA3, Hnf4a	+	ALB secretion, CYP1A2 and CYP3A4 activity	ALB, *α*1 antitrypsin, CYP3A4	Hepatotoxicity	^642^
Fibroblast, heart, tail tip and MEF	Cardiomyocyte	Heart	Sendai virus transduction	Gata4, Mef2c, Tbx5	+	Exhibited action potentials, beating cardiomyocyte cells, sarcomere formation	TNNT2, *α*-actinin	Myocardial infarction	^643^
Pericyte	Neurons	Brain	Retroviral transduction	Ascl1 and Sox2	+	Exhibited action potentials, synapses	SNAP25, MAP2, VIP, SST, CCK	Neuronal degeneration	^644^
Fibroblast, lung	Neuronal	Nerve	Small molecule cocktail	CHIR99021, DMH1, RepSox, forskolin, Y-27632, SP600125	+	Neuronal cells	BRN2, MYTL1, TAU, NEUROD1, NGN2, MAP2, TUJ1, NEUN	Neurologic disease	^492^
Glial cells	Neurons	Nerve (ear)	Plasmid transfection	Ascl1, NeuroD1	+	Neurite extensions	TUJ1, MAP2, VGLUT1, PROX1	Sensorineural hearing loss	^645^
Glioblastoma	Neuron	Brain, cancer	Small molecule cocktail	forskolin, ISX9, CHIR99021 I-BET 151, DAPT	+	Lost tumor spheroid formation	NGN2, ASCL1, BRN2, MAP2	Glioblastoma multiforme	^479^
HEK and MEF	Hepatocyte	Liver	Lentivirus transduction; decellularized liver culture	FoxA3-T2A, Hnf1a-E2A, Gata4	+	Metabolic/enzyme activity	CDH1, ALB, CYP3a11, ABCC2, AFP, HNF4A, transthyretin, transferrin	Chronic liver disease	^646^
Fibroblast, skin	Smooth muscle cells	Heart	Lentivirus transduction	Myocd, Gata6, Mef2C-fused with transactivation domain of MyoD	−	Smooth muscle cell contractility	MYH11, ACTA2, CNN1, SMTHB, TAGLN	Aortic aneurysm, atherosclerosis, stroke	^625^
Astrocytes	NSCs	Spinal cord	Lentivirus transduction	Zfp521	+	NSCs differentiated into neurons, astrocytes, and oligodendrocytes	DCX, Nestin, TUJ1, SOX2, NF200, GFAP, OLIG2, GLAST, GABA-A receptor	SCI	^647^
Fibroblast, skin	Cardiomyocyte	Heart	Gold nanoparticle transduction	Gata4, Mef2c, Tbx5	+	Reduced fibrosis and infarct area	TNNT2, ANP, MYH6, MYL2, HAND2, ANF, F-Actin, *α*MHC,	Myocardial infarction	^324^
Fibroblast, MEF	Oligodendrocyte progenitor cells	Nerve	Small molecule cocktail	CHIR99021, LDN193189, A83-01, Hh-Ag1.5, retinoic acid, SMER28, RG108, Parnate, bFGF	+	Myelinating activity	OLIG2, NKX2.2, PLP1, A2B5, NG2	Demyelinating disease	^503^
Astrocyte	Neuron	Nerve	Small molecule cocktail	CHIR99021, DAPT, LDN193189, SB431542	−	Neurogenic cells	NEUN, MAP2, ROBO2, SLIT1, SYP	Neuronal degeneration and damage	^480^
Urine cells	Neuron	Nerve	Small molecule cocktail	CHIR99021, A8301, Y-27632, TTNPB, forskolin, VPA, NaB	+	Partial electrophysiologic activity	Nestin, PAX6, TUJ1, MAP2, DCX, NEUN, GFAP	Neuronal degeneration	^510^
Fibroblast, MEF	Uterine glandular epithelium	Uterus	Small molecule cocktail	FGF2, BMP4, mLif, 1,4-DPCA, A8–301, CHIR99021	+	Uterine glandular epithelium	KRT19, EPCAM, CDH1, KI67, SPRR2f, LTF, FOXA2, SOX17	Absolute uterine factor infertility	^457^
Urine cells	Hepatocyte	Liver	Small molecule cocktail	CHIR99021, RepSox, VPA, Parnate, TTNPB; Dznep + 1 TF (Foxa3, Hnf1*α*, or Hnf4*α*)	+	Glycogen accumulation, LDL absorption, cytoplasmic accumulation of neutral triglycerides and lipids, albumin secretion	ALB, ASGPR1, CK18, Hnf4*α* TTR, GJB1	Liver failure, liver metabolic disorders	^495^
Fibroblast, skin	Chondrogenic cells	Cartilage	Electrical stimulation	C-Pace electroporation culture pacer (5 V/cm)	+	Protein secretion, hyaline cartilaginous matrix	COL2A1, AGC, SRY, SOX9	Cartilage degeneration	^648^
Fibroblast, foreskin	Neuron	Nerve	Episomal transfection	Ascl1, miR124, shP53	+	Neuronal cells	TUJ1, MAP2, NEUN, NCAM	Neuronal degeneration	^649^
Fibroblast, foreskin	Leydig-like cells	Testes	Lentivirus transduction; small molecule	Sf1; forskolin, DAPT, purmorphamine	+	Testosterone secretion	STAR, CYP11A1, CYP17A1, HSD3B1	Hypogonadism	^488^
Astrocytes	Neuron	Brain	Small molecule	CHIR99021, DAPT, SB431542, LDN193189	+	Spontaneous synaptic activity, action potentials	NEUN, KI67, NESTIN, DCX, TUJ1, PROX1, CTIP2, TBR1, VGLUT1	Neuronal degeneration and injury	^323^
Hepatic stellate cells	Myofibroblast	Liver	Exosomes in culture	Exosomal miR-192; reversible with anti-miR-192	+	Myofibroblasts	TGF-*β*1, COL1A1, *α*-SMA	Liver fibrosis	^650^
Fibroblast, MEF	Neurogenic	Skin	Nanochannel electroporation (NEP)	Ascl1, Brn2, and Myt1l (neurogenic NEP_ABM_)	+	Neurogenic fibroblasts	NF200, NGF, NT3	Diabetic peripheral neuropathy	^172^
Astrocyte	Neuron	Spine (CNS)	Small molecule cocktail	Kenpaullone, forskolin, purmorphamine, Y-27632, retinoic acid	+	Exhibited action potentials	TUJ1, MAP2, NEUN, Synapsin 1, HB9, ISL1, CHAT. VAChT	ALS	^517^
Fibroblast, heart	Cardiomyocyte	Heart	Lentivirus transduction; small molecule	Gata4, Mef2c, Tbx5, Hand2, Myocd, miR590; sodium butyrate, ICG-001, retinoic acid	+	Spontaneous cardiomyocyte beating, contractility	TNNT2, MYH6, NKX2.5	Heart failure	^526^
Urine and renal cells	Neuron	Brain	Small molecule cocktail	VPA, CHIR99021, RepSox, forskolin, SP600625, GO6983, Y-27632, ISX9, I-BET151, retinoic acid Vitamin C and QVD-OPH	+	Voltage-gated Na^+^ and K^+^ currents	TUJ1, MAP2, TAU, PSA-NCAM	PD, AD, traumatic brain injury	^518^
Fibroblast, lung	Myofibroblast	Lung	Silica-exposed exosomes	miR-125a-5p	−	Myofibroblasts, suppressed fibrosis gene expression	*α*-SMA, fibronectin, collagen I	Lung silicosis	^651^
Fibroblast, heart	Cardiomyocyte	Heart	Lentivirus transduction; Tg mice	Gata4, Mef2c; shRNA Atg5, shRNA Becn1	+	Spontaneously beating cardiomyocytes, increased mitochondrial DNA	Cx43, *α*-MHC, TNNT2, ND2, COX1	Myocardial infarction	^652^
Fibroblast, foreskin	Neuroectodermal	Nerve	Small molecule cocktail	Dorsomorphin, trichostatin A	+	Neuroectodermal cells	PAX6, vimentin	ALS, PD	^493^
Fibroblast, foreskin	Neuron	Nerve	Culture medium	FORSKOLIN, RepSox, SP600125, CHIR99021, Go6983, Y-27632, IXS9. and I-BET151	+	Membrane current properties	TUJ1, MAP2, NEUN, GABA, vGLUT1	AD, Huntington disease, epilepsy	^527^
Urine cells	Hepatocyte	Liver	Lentivirus and CRISPR knockdown	Hnf1a, FoxA3, and Gata4; CRISPR/Cas9 knockdown of P62, Becn1	+	Glycogen storage, lipid uptake	ICG, CYP450, ALB, AAT, CK18	End-stage liver disease	^653^
Adipocyte	Fibroblast	Skin	Retrovirus transduction	Elf4, FoxC2, FoxO1, Irf1, Prrx1, Zeb1	+	Spindle-shaped dermal fibroblasts	THY1, VCAM1, vimentin, actin, anti-ER-TR7	Skin wound	^654^
Fibroblast, heart	Cardiomyocyte	Heart	miRNA transfection	miRNAs (miR-1, 133, 208, and 499)	−	Calcium oscillation	GATA4, MEF2C, TBX5, HAND2, NKX2.5	Ischemic heart disease	^655^
Fibroblast, skin	Neuronal	Brain	Plasmid transfection; lentivirus transduction	Plasmid: microRNA-9/9*, microRNA-124. lentivirus: Ascl1	+	Formed synapses, calcium transients	TUJ1, MAP2, SYP, PSD95	Neurologic disease	^656^
Fibroblast, ear skin	Epithelial cells	Mammary gland	Small molecule cocktail	VPA, tranylcypromine, forskolin, TTNPB, RepSox	+	Mammary epithelial cells	KRT-18, PRLR, ACACA, EpCAM, B4GALT3, LTF, CSN2	Epithelial lactation mechanisms	^497^
Astrocyte	Neuron	Brain	Small molecule	Forskolin, CHIR99021, ISX9, I-BET151, Y-27632, DBcAMP	+	Exhibited action potentials and inward currents	TUJ1, MAP2, NEUN, SN1, GAD67	Neuronal degeneration and injury	^326^
Fibroblast, heart	Cardiomyocyte	Heart	Nanoparticle incubation	MicroRNA1, 133, 208, and 499	+	Spontaneous Ca^2+^ flux, beating cardiomyocyte cells	TNNT2, KCNJ2, SCN5a, MYH6, TNNI3, GATA4, MEF2C, TBX5	Myocardial infarction, heart failure	^327^
Fibroblast, pig embryonic	Skeletal muscle	Muscle	Lentivirus transduction; small molecule	FUW-tetO-pMyoD1; FGF2, SB431542, CHIR99021, forskolin	+	Formed striated sarcomere structure	Exo-MYOD1, Endo-MYOD1, PAX7, MYF5, MYOG	Skeletal muscle diseases	^524^
Hepatocyte	Hepatocyte progenitor	Liver	Small molecule cocktail	HGF, A-83–01, CHIR99021	+	Hepatic progenitor cells	KI-67, PCNA, CK19, CD44, SOX9	Liver disease	^465^
Stromal (bone marrow)	Neural precursors	Brain	Small molecule cocktail	Triiodothyronine and curcumin	−	Neural precursor cells	PAX6, SOX2, DLX2, GAP43	Neurologic diseases	^487^
Macrophage	Neuron	Brain	miRNA transfection; small molecules	miR-9, 124, 155 and 224, separately; IBMX, NGF, retinoic acid	+	Calcium excitability	MAP2, TUJ1, MASH1, synapsin, NURR1, Nestin	Neuronal degeneration and injury	^505^
Bovine satellite cells	Adipogenic bovine satellite cells	Adipose	Small molecule	Ciglitazone	+	Lipid accumulation	PPARγ, C/EBP*α*, C/EBP*β*, fatty acid synthase, stearoyl-CoA desaturase, perilipin 2	Fatty liver disease	^484^
Astrocyte	Neuronal	Spinal cord	AAV-CRISPRa transduction; small molecule	VP64 (Ngn2, Isl1 activator); Y-27632	+	Neurite extensions, spontaneous postsynaptic currents, action potentials	TUJ1, MAP2, NEUN, HB9, ChAT, SYT1	SCI	^657^
Fibroblast, heart	Cardiomyocyte	Heart	Lentivirus transduction	Gata4, Mef2c, Tbx5, Tead1	−	Contractility, calcium transients	TNNT2, MYH6, ACTC1, (SCN5A, HCN4)	Heart disease	^658^
Astrocyte	Neuron	Brain	Lentivirus transduction	NeuroD1	+	Increased motility, action potentials, sodium and potassium currents	TUJ1, synaptophysin, SYN1	Ischemic stroke	^659^
Fibroblast, heart	Cardiomyocyte	Heart	Nano dot-miRNA epicardium injection	miRNA-1, miRNA-133, miRNA-208, miRNA-499	−	Cardiomyocyte contractile activity	GATA4, MEF2C, NPPA, MYH7, NKX2-5, TNNT2, HAND2	Myocardial infarction	^328^
Oligodendrocyte precursor cells	Neuron	Brain	Plasmid transfection	Dlx2	+	Action potentials	TUJ1, NG2, MAP2, GAD67	Neuronal degeneration	^660^
Fibroblast, MEF	Myofibroblast	Lung	Exosomes	Silica-exposed macrophage exosomes (SPP1+)	−	Myofibroblasts	*α*-SMA, collagen I, fibronectin, COL1	Lung silicosis	^661^
Muller cells	Bipolar cells neuron	Retina	Small molecule cocktail	dbcAMP, forskolin, ISX9, CHIR99021, I-BET151, Y-27632	+	Exhibited action potentials	MAP2, TUJ1	Retinal degenerative diseases	^491^
Astrocytes	Neuron	Spinal cord	Lentiviral transduction; or small molecule	shRNA NOTCH1; or DAPT	+	Neuronal cells	TUJ1, MAP2, DXC	Neuronal degeneration and injury	^489^
Liver cancer cells, HepG2 and Hep3B	Benign hepatocytes	Liver	Small molecule intratumor injection	SB431542, CHIR99021, BIX01294, all-trans retinoic acid	−	Growth arrest, increased apoptosis, reduced migration and invasion; glycogen storage, lipid metabolism	HNF4*α*, CYP1A2, CYP3A4, AAT, ALB, OCT4	Liver cancer	^329^
Fibroblasts, skin	Neural	Brain	Small molecule cocktail	Melatonin, VPA, CHIR99021, forskolin, 1,9 pyrazoloanthrone (SP600125), Y-27632	+	Neural cells, autophagy activation	BRN2, ASCL1, MYT1L, DCX, Sox2, NeuN	PD, AD, and Huntington disease	^481^
Fibroblast, foreskin	Neuron	Nerve	Small molecule	Blebbistatin	+	Synaptogenesis, action potentials	TUJ1, MAP2, NEUN, SYN1	Neuronal degeneration	^475^
Fibroblast, skin	Neural precursor	Brain	cmRNA transfection; small molecules	Sox2 and Pax6 cmRNA; vaporic acid and RepSox	+	Neural precursor cells	PAX6, FOXG1, NGN2, TBR2, CTIP2, SATB2, TUJ1, vGLUT1	Neuronal degeneration model	^533^
Fibroblast, skin	Neuron	Brain	Small molecule cocktail	VPA, RepSox, forskolin, SP600125, GO6983, Y-27632 -OR- CHIR99021, LDN193189, RG108, dorsomorphin, P7C3-A20, A83-01, ISX9, forskolin, Y-27632, DAPT, PD0325901, purmorphamine, P7C3-A20	+	Misfolded protein accumulation, mitochondrial abnormalities	TUJ1, DCX, MAP2, Synapsin I, NEUN, TAU	AD, PD	^462^
Astrocyte	Neuron	Brain	Small molecule cocktail	Y26732, DAPT, RepSox, CHIR99021, ruxolitinib, SAG	+	Exhibited action potentials	TUJ1, DCX, MAP2, NEUN, SYN1, NEUROD1, ASCL1, NGN2, TB1, vGLUT1	AD, PD	^521^
Fibroblast, heart	Cardiomyocyte	Heart	Lentivirus transduction	Mef2c, Gata4, Tbx5, Tbx20, and miR-133	+	Contractility, calcium oscillation, action potentials	TNNT2 *α*-actinin, MYH6, MYH7, MYL2, TNNT2, ACTN2, MYBPC3	Injured myocardium	^662^
Fibroblast, (MELAS patients)	Neuron	Nerve	Lentivirus transduction	Acsl1, Brn2, shRNA targeting REST complex	+	Action potentials, decreased mitochondrial function	TAU, MAP-2	Mitochondrial encephalomyopathy, lactic acidosis and stroke-like episodes (MELAS)	^663^
Fibroblast, heart	Cardiomyocyte	Heart	Lentivirus transduction	Gata4, Mef2, Tbx5, Hand2/Myocardin, p63-transactivation inhibitory domain	+	Contractility, calcium transients	TNNT2, *α*-actinin, GJA1, MYH6	Congestive heart failure	^664^
Astrocyte	Neuron	Brain	Retroviral transduction; small molecules	NeuroG2, Cend1; forskolin, VPA	+	Neural precursor cells	TUJ1, MAP2, NEUN, GABA, TH, GLUT, synapsin1, PSD95	Neuronal degeneration and injury	^500^
Fibroblast, heart	Cardiomyocyte	Heart	miRNA transfection; 3D hydrogel	miR-1, 133, 208, and 499; 3D fibrin hydrogel	−	Calcium transients and release	TNNT2, MYL7, SCN5A, ACTC1, CACNA1C	Ischemic heart disease	^665^
Fibroblast, heart	Cardiomyocyte	Heart	PLGA-PEI nanoparticle transfection	miR-133, 5'AZA	+	Epigenetic enhanced reprogramming efficiency	TNNT2, GATA4, MEF2C, TBX5, HAND2, NKX2.5	Cardiovascular disease	^460^
Osteoblast (subchondral bone)	Chondrocyte	Cartilage	Lentivirus transduction	Sox9, Sox5, and c-Myc or Plagl1	+	2 chondrocyte subpopulation products	ALP, RUNX2, OCN, ACAN, COL9A1–3, COMP	Osteoarthritis	^666^
Fibroblast, heart	Cardiomyocyte	Heart	Lentivirus transduction; small molecules	Mef2c, Tbx5; SB431542, Baricitinib	+	Calcium transients, beating cardiomyocyte cells, action potentials	MYL2v, *α*-actinin, cTN1, TNNT2, MYH6, RYR2, NPPA, GJA1	Injured cardiac tissue	^472^
Melanocyte	Skeletal muscle	Muscle	Small molecule cocktail	Kenpaullone, forskolin, SB431542, SB203580	+	Contractility, myotube formation	TNNT2, TNNT3, Myf4, RYR1, MCK, MYOG	Skeletal muscle degenerative diseases	^523^
Inner ear supporting cells	Cochlear hair cells	Skin	Small molecule and adenovirus transduction	VPA; siRNA Fir, siRNA Mxi1	+	Hair cells	MYO7A, SOX2, ESPNFM1-43, POU4F3, SLC26A5, Ac-TUBA4A, NF-H, PVALB, ATOH1	Hearing loss	^509^
Hepatocyte	Hepatic progenitors and organoid	Liver	Small molecule cocktail	A83-01, CHIR99021, HGF	+	Hepatic progenitor cells and organoids	E-CAD, EPCAM, KI-67, ALB	Hepatitis, cirrhosis, fatty liver disease	^463^
Astrocyte	DA neuron	Brain	Retrovirus transduction	Nurr1, SHH, Bclxl, Ascl1, Ascl1, 5SA	+	Dopamine secretion	TH, TUJ1, NURR1, FOXA2, MAP2, NEUN, synapsin 1	PD	^670^
Fibroblast, skin	Cardiomyocyte	Heart	Lentiviral transduction; small molecule cocktail	miR-2392; forskolin, VPA, CHIR99021	−	Cardiomyocyte cells	TNNT2, GJA1, ɑ-MHC	Ischemic heart disease, myocardial infarction	^482^
Endothelial	Macrophage	Heart	EV nanotransfection	C/EBP*α* + Spi1 (EVs derived from dermal fibroblast)	+	Anti-inflammatory macrophages	CD163, CD31, Spi1, CD68	Valvular heart disease	^671^
Fibroblast, heart	Cardiomyocyte	Heart	Lentivirus transduction; small molecule	Myocd, Smad6, Tbx20; Xav939	+	Exhibited contractility, calcium transients	ACTC1, ACTN2, MYH6, MYH7B, MYL2, MYL3, MYL4, MYL7, TNNT2, TNNC1, TTN	Heart disease	^536^
Fibroblast (MEF) (hFCF)	Cardiomyocyte	Heart	Plasmid transfection; small molecule	Gata4, Mef2c, Tbx5 (GMT); vitamin C	+	Improved GMT reprogramming efficiency, reduced ROS production	MYH6, ACTN2, TNNT2, ACTC1	Myocardial infarction	^535^
Fibroblast, skin	Dermal papilla	Skin	Hydrogel microspheres—nanoparticle	Tideglusib and tamibarotene	+	Dermal papilla cells that promote keratinocyte proliferation and activation in co-culture	VCAN, ALP, BMP2, BMP4, ALPL, LEF1, *β*-CATENIN, FOXO1	Skin wounds	^343^
Fibroblast, MEF	Hepatocytes	Liver	AAV CRISPRa transduction	gRNA: Gata4, Foxa3	+	Glycogen storage, DiI-ac-LDL uptake, drug metabolism	E-cadherin, ALB	Liver fibrosis	^672^
Astrocytes	Neuronal	Spinal cord	Small molecule	LDN193189, CHIR99021	+	Neurite extension, synaptic connections	DCX, TUJ1, MAP2, NEUN, SYN1	SCI	^344^
Fibroblast, heart	Cardiomyocyte	Heart	Small molecule cocktail	CHIR99021, VPA, dorsomorphin, SB431542, forskolin	+	Spontaneous calcium oscillation	TNNT2, Cx43, *α*-actinin, TBX5	Cardiovascular disease	^494^
Keratinocyte	Myoepithelial cells	Skin	Small molecule cocktail	VPA, SB431542, retinoic acid, tranylcypromine, cyclic pifthrin-*α*, BMP4, EGF	+	Acetylcholine responsive, contractility, actin filament bundles	*α*-SMA, VIM, CXCR4, MYH11	Skin wounds	^349^
Cochlear hair supporting cells	Cochlear hair cell	Ear	Small molecule cochlea	PD0325901	+	Hair cell regeneration ex vivo	MYO7A, p-ERK, Atoh1, KI67	Hearing loss	^350^
Fibroblast, skin	Sertoli cells	Gonad	Lentiviral transduction	Fgf2, Gata6, Gata4, Mxi1, JunB, NfyB	+	Adhesion properties (necessary for tubule development)	SOX9, PTGDS, BMP4, DMRT1, KRT18, INHBA, NCAM1/2, TGFA, GADD45G, ZFPM, IL1A, CX43/GJA1	Disorders/differences of sex development	^673^
Fibroblast, skin	Neuronal	Nerve	Episomal transfection	SOX2, PAX6	+	Voltage-gated sodium channels	TUJ1, MAP2, NEUN, VLGLUT1	Rett syndrome	^674^
Fibroblast, skin	Hepatocyte	Liver	Lentivirus transduction: CRISPR plasmid transfection	Foxa2, Hnf1a, Hnf4a and Tbx3; CRISPR-Cas9 Agxt knock-in	+	Decreased oxalate accumulation in Agxt-corrected iHeps	LDH-A, AGT, oxalate, glycogen	Primary hyperoxaluria type 1	^675^
Urine	Intestine organoid	Intestine	Small molecule cocktail	CHIR99021, EPZ5676, B27, *β*-ME, RG108, P8511, Activin A, LYZ294002, EPZ011989, Noggin, SB431542, BMP4, retinoic acid	+	Intestinal barrier function	CDX2/MUC2, CDX2/CHGA, CDX2/LYZ, VIL/CHGA	Intestinal disease	^469^
Fibroblast, heart	Cardiomyocyte	Heart	EVs	Cardiomyocyte EVs	+	Mature cardiomyocyte cells	cTNT, *α*-actinin, RYR2, NKX-2.5, ISL-1, MESP-1, GATA4, FLK-1, CX43	Myocardial infarction	^351^
Fibroblast, heart/lung	Endothelial	Heart and lung	Retroviral transduction	Sox17, Erg	−	Endothelial cells	CDH5, PEACAM1, GJA5, KDR, NOTCH1, EFNB2, VWF, DLL4	Heart and lung disease	^676^
Fibroblast, MEF/fCF	Cardiomyocyte	Heart	Lentiviral CRISPRa/dCas9	Mef2c, Gata4, Tbx5	−	Cardiomyocyte cells	ACTC1, TNNT2, MYH6, MYL4, MYL7, PLN, SLC8A1	Myocardial infarction	^677^
Fibroblast, skin	Neuron	Skin	Lentiviral transduction	miR-9/9*, miR-124; SAHM1 peptide	+	Improved neurite outgrowth	FOSB, NR4A2, EFNA2, LINC01089, CDH12, KCNMA1, DCC	Neural developmental disorders	^678^
Fibroblast, MEF	Cochlear spiral ganglion neuron	Ear	Lentivirus transduction	Ascl1, Pouf1, Myt1l	+	Voltage-gated sodium and potassium channels, exhibited action potentials	TUJ1, MAP2, SYP, PROX1, PRPH, SCRT2, GATA3, ISL1, NTRK2, SYT4, NTRK3, CNTNAP2	Sensorineural hearing loss	^679^
Urine	Motor neuron	Nerve	AAV transduction; small molecule	hNGN2, mSOX11, hISL1, hLHX3; forsolin, dorsomorphin	+	Spontaneous calcium oscillation, formed NMJ in co-culture	ChAT, HB9, TUJ1, SMI32	Motor neuron diseases, ALS	^680^
Fibroblast, skin	Astrocyte	Brain	Small molecule cocktail	SB431542, A83-01, CHIR99021, forskolin, CNTF, BMP4, LIF	+	Exhibited glutamate uptake, ionic buffering, cytokine and spontaneous calcium responses	GFAP, S100*β*, CD90/VIM, GJA1, NFIA, NFIB, APOE, SLC1A3	Late-onset neurologic disorders	^485^
Fibroblast, skin	Neuron	Brain	Lentivirus transduction; 3D culture	MYT1L, NEUROD2, miR-9/9*-124; Matrigel	+	Exhibited action potentials, accumulation of A*β* deposits, tauopathy, bulged dystrophic neurites and neurodegeneration	NCAM1, MAP2, SLC17A7, VGLUT1, TUJ1	AD	^681^
Fibroblast, MEF, hESC	Neuron	Brain	Lentivirus transduction	Six3, Six6, Dlx2, Lhx1, Ascl1, Ngn2	+	Exhibited multipolar morphologies, calcium transients, spontaneous firing pattern	TUJ1, MAP2, NEUN, VGLUT1, VIP	Neurologic diseases with circadian disruption	^682^

**Table 4:** In vitro/ex vivo direct reprogramming followed by in vivo applications Outcome cell types listed do not necessarily qualify to be a fate change from the starting cell type. A fate-change call must be supported by evidence demonstrating that the outcome/product cell types have completely lost all characteristics (transcriptomic signature that would identify the product cell as a variant of the parent cell as determined by scRNA sequencing) of the parental cell while acquiring novel characteristics of the listed outcome/product cell. In the absence of such evidence, claimed transitions may be viewed as a form of state change of the parental cell, which may be transient until otherwise proven.

Starting Cell Type	Intended Reprogrammed Cell Type	Organ/Tissue Targeted	Method	Reprogramming Molecule/Factor	Morphology	Functionality	Marker	Disease State	Reference
Fibroblast, adult skin and fetal limb	Hepatocytes	Spleen	Lentivirus transduction; then transplant	FoxA3, Hnf1A, Hnf4A	+	Lipid uptake, cytochrome P450 enzyme activity, biliary drug clearance; restore liver function, and prolong survival in vivo	ALB, ASGPR1, AAT, transferrin	Liver disease	^624^
Astrocyte	Neurons	Brain	Small molecule cocktail; then transplant	LDN193189, SB431542, TTNPB, thiazovivin, CHIR99021, VPA, DAPT, SAG, purmorphamine	+	Exhibited action potentials in vitro, integrated into brain neuron circuits, and formed synapses in vivo	DCX, TUJ1, MAP2	Neuronal degeneration and injury	^522^
Fibroblast, foreskin	Cardiomyocytes	Heart	Small molecule cocktail; then transplant	CHIR99021, A83-01, BIX01294, AS8351, SC1, Y-27632, OAC2, SU16F, JNJ10198409	+	Contractility, beating cells, remuscularized infarcted area in vivo	TNNT2, connexin 43, CAV3.2, HCN4, KIR2.1	Myocardial infarction	^471^
Fibroblast, skin	Osteoblasts	Bone	Plasmid transfection; then transplant	Oct4, Osterix, l-Myc	+	Calcified bone matrix in vivo	ALP, OC, OPN	Osteoporosis, rheumatoid arthritis, periodontitis	^865^
Astrocytes	Neuronal cells	Brain	Small molecule cocktail; then transplant	CHIR99021, RepSox, forskolin, i-BET151, ISX-9	+	Exhibited action potentials and synapse formation in vivo	TUF1, MAP2, TAU, SYN1	Neuronal degeneration and injury	^504^
Fibroblast, skin	Endothelial cells	Blood vessel	Lentivirus transduction; then transplant	ER71, ETV2	+	Vascular incorporation, ischemic repair in vivo	CDH5, KDR, TEK, PECAM1, CD34	Ischemic and injured tissue vasculature	^866^
Fibroblast, skin	Myoblasts	Skeletal muscle	Retroviral transduction; then injected subcutaneous	Mycl, MyoD1	+	Multinuclear myotubes and myofiber-like structure in vivo	MYOG, CKM, DES	Muscle disease and congenital muscle defects	^867^
Fibroblast, skin	Osteoblast	Bone	Retrovirus transduction; then transplant (NanoClip-FD gel matrix)	Osterix, Oct3/4, l-myc	+	Calcified bone matrix, bone regeneration in vivo	OC, OPN	Osteoporosis, bone injury	^868^
Fibroblast	Neurons	Brain	Plasmid transfection; hydrogel culture; then transplant	Brn2, Ascl1 and Myt1; hydrogel (brain ECM)	+	Action potential, synapses	TUJ1, MAP2, NeuN	Neuronal degenerative diseases	^869^
Fibroblast, foreskin	Neurons	Brain	Small molecule cocktail; then transplant	CHIR99021, LDN193189, RG108, dorsomorphin, P7C3-A20, A83-01, ISX9, forskolin, Y-27632, DAPT, PD0325901, purmorphamine	+	Action potential, synapses and local circuit integration in vivo	TUJ1, MAP2, TAU	Neurologic disorders	^512^
Hepatocyte	Hepatocyte progenitors	Liver	Small molecule cocktail; then transplant	A83-01, CHIR99021, HGF	+	Hepatic progenitor cells, repopulate injured liver in vivo	E-cadherin, AFP, CD44, CD90, CK19	Liver diseases	^464^
Fibroblast, skin	SCHWANN cells	PNS	Small molecule cocktail; then transplant	bFGF, forskolin, PDGF, heregulin-B1-EGF domain	+	Axon regeneration, NMJ rescue in vivo	S100B, p75, P0, GFAP, O4	Multiple sclerosis	^502^
Renal epithelial cells	Nephron progenitor cells	Kidney	PiggyBac transposon transfection; then transplant	Snai2, Eya1, Six1	+	Integrate into developing nephron tubules in vivo	SIX1/2, EYA1, MEOX2, OSR1, CITED1	Chronic kidney disease	^870^
Astrocyte and fibroblast, foreskin	Neurons	Heart	Lentivirus transduction; then transplant	Ascl1, Phox2b, AP-2a, Gata3, Hand2, Nurr1, Phox2a	+	Noradrenaline release, action potentials; integrate into neural circuits in vivo	TUJ1, MAP2, SYN1, VMAT2, NET, NPY, GAL	Noradrenergic neuron disorders (AD, PD, middle-aged Down syndrome)	^871^
Epidermal skin	Sweat gland cells	Skin	Lentivirus transduction; then transplant	FoxC1	+	Wound repair and sweat gland regeneration in vivo	K8, K18	Anhidrotic/hypohidrotic ectodermal dysplasia	^872^
Fibroblast, periodontal ligament	Leydig cells	Testes	Small molecule cocktail; then transplant	Forskolin, 20*α*-hydroxycholesterol, luteinizing hormone, SB431542	+	Androgen synthesis in testes in vivo	STAR, CYP11a1, HSD3b, CYP17a1, HSD17b3	Male hypogonadism	^458^
Fibroblast, MEF	Photoreceptor cells	Retina	Small molecule cocktail; then transplant	VPA, CHIR, RepSox, forskolin, IWR1, sonic hedgehog, taurine, retinoic acid	+	Restoration of the pupil reflex and visual function in vivo	ROR*β*, ASCL1, PIAS3, CRX, rhodopsin, recoverin	Multiple sclerosis	^528^
Fibroblast, skin	Neurons	Spinal cord	Lentivirus transduction; then transplant	Oct4, Lhx3	+	Formed synapses; NMJ formation, locomotor recovery in vivo	ISL1, TUJ1, HB9, MAP2, NKX6.1, CHAT	SCI	^873^
Fibroblast, MEF	Fibrocartilaginous cells	Cartilage	Small molecule cocktail; then transplant	VPA, CHIR98014, RepSox, TTNPB, and celecoxib	+	Cartilage matrix formation and rescue of mechanical function in vivo	SOX9, COL2, ACAN, PRG4	Articular cartilage injury and degeneration	^477^
Apical papilla	Endothelial cells	Blood vessels	Small molecule cocktail; subcutaneous injection (Matrigel)	VPA, CHIR99021, RepSox, forskolin, Y-27632, BMP4, 8-Br-cAMP	+	Generate vascular structures in vivo	CD31, VEGFR2, VEGFR1, TIE2, VE-Cadherin	Ischemic diseases	^461^
Glial cells	Neuron	Brain	Retrovirus; then transplant	Ascl, Dlx2	+	GABAergic neurons	DCX, NEUN, MAP2, GAD67, CALB2, SST, VIP	Epilepsy	^874^
Fibroblast, skin	Adipocytes	Skin/adipose	Small molecule; then injected to supraperiosteal plane of the skull	STK287794	+	Lipid accumulation and formed granulation tissue containing white and brown adipocytes in vivo	STK287794. C/EBP*α*, PPARγ	Fill voids following surgical resection (eg, tumor removal)	^171^
Fibroblast, MEF	Corneal endothelia	Cornea	Small molecule cocktail; then transplant	CHIR99021, SB431542, forskolin, VPA, EPZ004777, 5-Aza	+	Self-renewal capacity, reverse corneal opacity in vivo	Na+/K+ATPase, AQP1, ZO-1, N-Cadherin	Corneal disorders	^496^
Fibroblast, ear skin	Mammary epithelial cells	Mammary gland	Small molecule cocktail; then transplant	TTNPB, forskolin, RepSox, tranylcypromine, VPA	+	Regenerate mammary gland structure in vivo	CDH1, EPCAM, ITGA6, KRT19, KRT8, KRT18	Mammary gland defects	^498^
Fibroblast, MEF	Vasculogenic fibroblast	Brain	Plasmid TNT; then transplant	Etv2, Foxc2, and Fli1 (EFF)	−	Increased neuron cell density, motor recovery, reduced glial scar and infarct region, increased vascularity in vivo	VEGF-D, bFGF, NEUN, GFAP	Ischemic stroke	^854^
Hepatocytes	Hepatic progenitor cells	Liver	Culture medium; 3D scaffold transplant	A83-01, CHIR99021 and a 3D scaffold	+	Hepatic progenitors, restore bile flow in vivo	CK7, CK19, CFTR, SOX9, CK19	Cholangiopathy	^466^
Fibroblast, primary MEF	Cardiomyocytes	Heart	Small molecule cocktail; then transplant	CHIR99021, A83-01, forskolin, SC-1; 3D heart ECM	+	Beating cardiomyocytes, calcium transients in vitro; improved cardiac function in vivo	TNNT2, MEF2C, MYH7, and NKX2-5	Myocardial infarction	^525^
Erythroblast	Megakaryocytes	Blood	Small molecule cocktail; then transplant	Bix01294, RG108, VPA, PD0325901	+	Fibrin clot and platelet plug formation in vitro; platelet production in vivo	CD41, CD42b, vWF	Thrombocytopenia	^519^
Fibroblast, neonatal skin	Dermal papilla	Skin	Small molecule; then transplant	TTNPB	+	Hair follicle formation in vivo	ALP, *α*-SMA, CRABP1	Hair loss	^531^
Fibroblast, skin	Sweat gland cells	Skin	Small molecule cocktail w/EDA; then transplant	RepSox, CHIR99021, isoproterenol, retinoic acid, BMP4; EDA (ectodermal dysplasia antigen)	+	Ach-dependent calcium transients, sweat gland regeneration in vivo	CK5, CEA, CK10, AQP5, CK18	Skin wounds	^476^
Epithelial retinal pigment cells	Photoreceptor cells	Retina	Small molecule cocktail; then transplant	VPA, CHIR99021, RepSox, forskolin, IWR1	+	RCVRN^+^ photoreceptor cells that survived in vivo	SOX8, IGFN1, ASCL1, RXRG, THRB, RORB	Retinal degenerative diseases	^506^
Damaged liver hepatocytes	Hepatic progenitor cells	Liver	Small molecule cocktail; then transplant	Y-27632, A-83–01, CHIR99021	+	Hepatic progenitor cells, survived in vivo without tumorigenesis	EpCAM, SOX9, CK19, CD133	Liver injury and cirrhosis	^467^
Fibroblast, skin	Osteogenic cells	Bone	Small molecule cocktail; then transplant (in Matrigel)	RepSox, forskolin, phenamil	+	Bone matrix formation in vivo	RUNX2, BSP, ALP, OCN	Osteoporosis, bone injury	^515^
Fibroblast, MEF	Sertoli cells	Testes	Small molecule cocktail; then transplant	I-BET151, retinoic acid, riluzole	+	Engulfment of apoptotic sperms in vitro; formation of testicular seminiferous tubules in vivo	Wt1, GATA4, glial cell line–derived neurotrophic factor, KRT18, AMH, AR (androgen receptor)	Sertoli cell infertility defect	^520^
Fibroblast, foreskin	Cardiovascular progenitor cells	Heart	Small molecule cocktail; then transplant	CHIR99021, A83-01, GSK126, forskolin, CTPB, AM580	+	Multilineage cardiovascular differentiation in vivo	Ki67, GATA4, ISL1	Myocardial infarction	^486^
ADRC	Cardiomyocytes	Heart	Lentivirus transduction; then transplant	Baf60c, Gata4, Gata6, Klf15, Mef2a, Myocd		Reduced scar area and improved survival in vivo	MYH6, ACTC1, TNNT2	Myocardial infarction	^875^
Fibroblast, skin	Vasculogenic	Skin	TNT, plasmid nanoporation, then transplant in collagen gel	Anti-miR200b oligonucleotide (ASO) (vasculogenic TNT_ASO_)	+	Vasculogenic fibroblasts in vitro, cord blood endothelial colony forming cells, blood vessel formation in vivo	COL1A1, COL5A2, COL3A1, VEGFB, VEGFC, NRP1, eNOS, ac-LDL, FLI1, CD31, VWF, lectin	Ischemic wounds	^112^
Fibroblast, neonatal skin and MEF	Hepatocytes	Liver	Small molecule cocktail; then transplant	CHIR99021, E-616452, forskolin, AM580, EPZ004777 -OR- CHIR99021, E-616452, forskolin, CH55, UNC0638, EPZ004777	+	Drug metabolism in vitro, liver repopulation in vivo	ALB, HNF4a, FOXA2, CPS1, CYP3a11	Liver failure	^470^
Fibroblast, foreskin (human) and MEF	Leydig cells	Testes	Small molecule cocktail; then transplant (in Matrigel)	Forskolin, DAPT, purmorphamine, 8-Br-cAMP, 20*α*-hydroxycholesterol, SAG	+	Express testosterone–synthesizing enzymes in vitro, promote testosterone recovery in vivo	NR5a1, CYP11a1, HSD3b1, HSD17b3, STAR	Male hypogonadism	^459^
Fibroblast, MEF	Hepatocytes	Liver	Small molecule cocktail; then transplant (in Matrigel)	SB431542, LDN193189, BIX01294, CHIR99021, DAPT	+	Lipid accumulation, ABL secretion in vitro, promote liver regeneration in vivo	HNF4*α*, HNF1*α*, ALB, CYP1A1, CYP1A2	Liver diseases	^473^
Fibroblast, rat embryonic	Neural progenitor cells	Brain	Small molecule cocktail; then transplant (in Matrigel)	VPA, CHIR99021, RepSox	+	Neural progenitor cells, behavioral function rescue in vivo	SOX2, PAX6, Nestin, ASCL1	Neuronal degeneration	^483^
Fibroblast, subcu. connective tissue (L929 line)	Osteoblast	Bone	Plasmid transfection; then transplant (in collagen gel)	Runx2, Dlx5	+	Osteocalcin secretion, mineralized bone tissue formation in vivo	ALP, OCN, ROCK, PI3K	Osteoporosis, bone injury	^876^
Fibroblast, skin	Insulin-producing cells (IPC)	Pancreas	AAV transduction; then transplant	Neurog3, Pdx1, MafA, Pax4, Nkx2-2	−	Glucose-induced insulin secretion in vivo	INS, GCG, SST, NEUROD1, INSM1, PAX4, NKX2-2, ARX	Diabetes	^626^
Fibroblast, skin	Neuron	Brain	Small molecule cocktail; then transplant (in Matrigel)	Forskolin + neuronal medium (neurobasal, cAMP, bFGF)	+	Exhibited action potentials in vitro, survival >2 months in vivo	ASCL1, NEUROG2, NEUROD1, NEUN, MAP2, TUJ1	Neuronal degeneration	^499^
Fibroblast, skin	Cardiomyocytes	Heart	Plasmid transfection; then transplant	Gata4, Mef2c, Nkx2.5; +/− Oct4, Klf4, Sox2, cMyc	+	Contraction, reduced fibrosis and improved ventricular wall thickness in vivo	GATA4, cMHC, MEF2c, NKX2.5, cTN1, TNNT2	Myocardial infarction	^877^
Astrocyte	Oligodendrocyte progenitor cells	Brain	Small molecule cocktail; then transplant (in Matrigel)	CHIR99021, forskolin, RepSox, LDN193189, VPA, Thiazovivin	+	Differentiation to mature oligodendrocytes in vivo	PDGFR*α*, KI-67	Multiple sclerosis	^529^
Fibroblast	Cardiac endothelial cells	Heart	Retrovirus transduction; then transplant	Sox17, Erg	+	Uptake Ac-LDL, produce NO in vitro, increased perfusion in infarcted region in vivo	PECAM1, CDH5	Myocardial infarction	^878^
Fibroblast, skin	Vasculogenic	Skin	TNT, plasmid nanoporation; then transplant in collagen gel	Vasculogenic TNT_EFF_	+	Vasculogenic fibroblasts in vitro, chimeric capillary formation in vivo	VEGFR2, PECAM1, CDH5, COL1A2, VWF, TET1/2/3, 5-hmC, lectin, THY1, S100A4, VIM, COL1A1, FBLN1	Ischemic wounds	^140^
Keratinocyte, HEK	Sweat gland cells	Skin	Small molecule cocktail; then transplant	CHIR99021, PMA, forskolin, VPA, tranylcypromine, A83-01	+	Functional sweat gland cells in vitro, promoted wound closure, and regenerated sweat glands in vivo	CK1, CK10, CDH3, LHX2, SSEA-4, CK14	Skin wounds	^516^
Fibroblast, MEF	Chondrocytes	Cartilage	Replication-defective and persistent Sendai virus (SeVdp) transduction; then transplant	Sox9_H131A/K398A_, Klf4, c-Myc	+	Cartilage-like tissue formed in vivo	COL2A1, COL11A2, ACAN	Osteoarthritis	^879^

**Table 5:** In vivo viral direct reprogramming and functional outcome Outcome cell types listed do not necessarily qualify to be a fate change from the starting cell type. A fate-change call must be supported by evidence demonstrating that the outcome/product cell types have completely lost all characteristics (transcriptomic signature that would identify the product cell as a variant of the parent cell as determined by scRNA sequencing) of the parental cell while acquiring novel characteristics of the listed outcome/product cell. In the absence of such evidence, claimed transitions may be viewed as a form of state change of the parental cell, which may be transient until otherwise proven.

Target Site	Intended Reprogrammed Cell Type	Organ/Tissue Target	Method	Reprogramming Molecule/Factor	Morphology	Functionality	Marker	Disease State	Reference
Peri-infarct myocardium	Cardiomyocyte	Heart	Lentivirus injection	miRNA 1, 133, 208, and 499	−	Cardiomyocytes	TNNT2, *α*MHC, CFP	Cardiac injury	^629^
Peri-infarct myocardium	Cardiomyocyte	Heart	Retrovirus injection	Gata4, Mef2c, Tbx5	+	Calcium transients, increased cardiac output	*α*-Actinin, *β*-galactosidase, N-cadherin, CX43, MYL7, SCN5a, SLC8a1, RYR2, MYH6, MYL2, TNNT2, ACTC1, PLN	Myocardial infarction	^852^
Left striatum	Neuroblast	Brain	Lentivirus injection; intraperitoneal small molecule injection	miR-302/367 GFP-tagged; VPA	−	GFP-neuroblast detection	GFAP, OLIG2, PLP, DCX, TUJ1, NeuN	Neuronal degeneration and injury	^534^
Peri-infarct myocardium	Cardiomyocyte	Heart	Lentivirus injection	miRNAs 1, 133, 208, or 499	+	Action potentials, excitation contraction, improved cardiac function	TNNT2, CX43, *α*-actinin	Myocardial infarction	^881^
Striatum, oligodendrocyte	Neuron	Brain	Oligodendrocyte-targeting AAV intracranial injection	miRNA Ptbp1	+	Action potentials, spontaneous postsynaptic currents	OLIG2, NEUN, DARRP32, parvalbumin,	Cortical injury	^882^
Peri-infarct myocardium	Cardiomyocyte	Heart	Adenovirus or lentivirus injection	Gata4, Mef2c, Tbx5	−	Cardiomyocyte generation, improved ventricular function and ejection fraction	TNNT2, *α*-actinin, MYH7, Vimentin	Myocardial infarction	^635^
Striatum	Neuron	Brain	AAV injection; Ng2 Cre mice	Ascl1, Lmx1a, Nurr1	+	Action potential, spontaneous postsynaptic activity	Parvalbumin, PV, GAD65/67	Neurologic diseases	^883^
Striatum	Neuron	Brain	Lentivirus injection	NeuroD1, Ascl1, Lmx1, miR218	+	Action potential and improved motor function	MAP2, TUJ1, TH, RBFOX3, NR4A2, PBX1	PD	^636^
Infarcted myocardium	Cardiomyocyte	Heart	Sendai virus injection	Gata4, Mef2c, Tbx5	+	Reduced fibrosis, improved ejection fraction and fractional shortening of left ventricle	TNNT2, *α*-actinin	Myocardial infarction	^643^
Spinal cord parenchyma	Neural cells	Spinal cord	Lentivirus injection	Zfp521	+	Spinal cord regeneration, improved behavioral and locomotor assessment	O4, CD86, NEUN, TUJ1, MAP2	SCI	^647^
Ipsilateral striatum	Neuron	Brain	Retrovirus injection	Ascl1, Sox2, NeuroD1	+	Long processes, synapse-like structures	NEUN, IBA1, GFAP, PDGFR*α*, DCX	Ischemic stroke	^884^
Intracortical	Neuron	Brain	Retrovirus intracortical injection	Ngn2, Bcl-2	+	Neuroblasts and mature neurons	NEUN, GFAP	Ischemic stroke	^885^
Spinal cord dorsal horn	Neuron	Spinal cord	AAV injection	NeuroD1	+	Exhibited action potentials, synaptic response	TLX3, NEUN, PAX2, CAMKII, cFOS	SCI	^886^
Motor cortex	Neuron	Brain	AAV stereotactic infusion pump	NeuroD1	+	Increased neuronal density and regeneration	TBR1, NEUN, GFAP, IBA1	Ischemic stroke	^887^
Peri-infarct myocardium	Cardiomyocyte	Heart	Lentivirus transduction; Tg mice	Gata4, Mef2c, shRNA Atg5; Becn1 haploinsufficient mice	+	Reduced scar size, improved cardiac ejection fraction and fraction shortening	*α*MHC, TNNT2, ACTC1, GJA1, SCN5a, KCNA5, *β*-catenin	Myocardial infarction	^652^
Peri-infarct myocardium	Cardiomyocyte	Heart	Sendai virus injection	Gata4, Mef2c, Tbx5	−	Reduced fibrosis, improved myocardial contraction and left ventricle ejection fraction	TNNT2, COL1, tdTomato-cardiac fibroblasts	Myocardial infarction	^888^
Hippocampus	Neuron	Brain	Retrovirus injection	Ascl, Dlx2	+	Action potential, synaptic connections, seizure reduction	DCX, NEUN, BrdU, IBA1, GFAP, OLIG2, NPY, VIP, SST	Epilepsy	^874^
Dorsal midbrain or spinal cord lumbar L1 to L2	Neuron	Brain and spinal cord	AAV stereotaxic injection, microinfusion pump	Neurog2	+	Exhibit action potentials, synapse formation, host circuit integration	NEUN, VGLUT2, Parvalbumin	Neuronal injury and disease	^889^
Spinal parenchyma	Neuron	Spinal cord	Lentivirus injection (NG2 promoter)	Sox2	−	Diverse neuronal subtypes, synaptic connections, reduced glial scarring, promoted functional recovery	DCX, MAP2, SYN1, NEUN, VGLUT2, GAD6, VGAT, GLYT2	SCI	^890^
Dorsal spinal cord parenchyma T9-T11	Neuron	Spinal cord	AAV-CRISPRa injection	VP64 (Ngn2, Isl1 activator)	+	Axonal projection to sciatic nerve	tdTomato-astrocytes, GFAP, NEUN, HB9, ChAT, GAD1,	SCI	^657^
Cerebral sensorimotor cortex	Neuron	Brain	Lentivirus injection	NeuroD1	+	Exhibit action potentials; partial rescue of cortical circuits, psychological, motor, and sensorimotor function	NEUN, VGLUT, TH, BDNF, FGF10	Ischemic stroke	^659^
Striatum	Neural progenitor cells	Brain	Lentivirus injection	DLX2	+	Neural projenitor cells differentiated into multiple neuronal subtypes	ASCL1, NEUN, KI67, DCX, CTIP2, ALDH1L1, ALDOC, CTIP2, SOX10, OLIG2, MBP	Neural degeneration and injury	^474^
Spinal parenchyma T8	Neuron	Spinal cord	Lentiviral intraspinal injection; or small molecule intraperitoneal injection DAPT	shRNA NOTCH1; or small molecule inhibitor DAPT	+	Synapse formation	DCX, TUJ1, MAP2, NEUN, GAD6, SYN1	Neuronal degeneration and injury	^489^
Middle ear	Cochlear hair cells	Skin	Small molecule and adenovirus injection	VPA, LiCl, FSK; siRNA Fir, siRNA Mxi1	+	Hair cell regeneration	ATOH1, SOX2, ESPN	Hearing loss	^509^
Infarcted myocardium	Cardiomyocyte	Heart	AAV injection	Myocd, Ascl1, miR-133	−	Improved left ventricle ejection fraction and stroke volume	TNNT2	Myocardial infarction	^667^
Cerebral cortex	Immature neuron	Brain	Lentivirus or AAV injection; small molecule	miRNA-124; ISX9	+	Immature neuron survival >8 wk in cortex	GFAP, NEUN, DCX, TBR1, MASH1, VGLUT2, PV, GPHN	Neuronal degeneration and injury	^668^
Spinal cord T9-T10	Neuron	Spinal cord	AAV-shRNA or ASO injection	shRNA-PTB or antisense-oligo-PTB (ASO)		Improved motor function (ASO/shRNA), reduced glial scar density and apoptosis (ASO)	ChAT, NEUN, MAP2, GFAP	SCI	^669^
Cerebral cortex	Neuron	Brain	Retrovirus injection	Fezf2, Lmo4, Neurog2, Bcl2	+	Layer V–like neurons; increased dendrite branch points, length, and number	CTIP2, DCX, NEUN	Neuronal degeneration	^891^
Hippocampus	Neuron	Brain	AAV injection	NeuroD1	+	Repaired synapses; increased dendritic spine length; and density; promoted neurogenic microenvironment	Ly6X, C3, PTX3, KI-67, Nestin, SOX2, NEUN, DCX, PSD95, SYN, IBA-1, iNOS	Subarachnoid hemorrhage	^892^
Cochleae	Cochlear hair cell	Ear	AAV injection	Gfi1, Pou4f3, Atoh1, Six1	+	Hair cell like cell regeneration, weak calcium fluctuation	Supervillin, ZEB1, CHD7, ANKRD6, OTOF, SLC2A5, TMC1, MYO7A	Hearing loss	^893^
Cerebral cortex	Neuron	Brain	AAV injection	shRNA-PTB	+	Improved motor function; vascular remodeling	NEUN, MAP2, GFAP, VGLUT1, CD31, IBA1	Ischemic stroke	^894^
Fibroblast (Col1a1-targeted)	Hepatocytes	Liver	AAV CRISPRa tail vein injection	gRNA: Gata4, Foxa3	+	Reduced fibrosis and liver injury marker alanine transaminase	FAH, MUP, *α*-SMA	Liver fibrosis	^672^
Spinal cord C5	Neuron	Spinal cord	Lentivirus injection	Sox2/p75-2	+	Corticospinal tract and serotonin axonal regeneration	GFAP, serotonin, CD68	SCI	^895^

## Data Availability

This review article contains neither newly generated data nor data analysis.

## Significance Statement:

Breakthrough technologies have transformed our understanding of cell and tissue biology, uncovering that cells and tissues possess remarkable adaptability and fluidity in their roles. This revelation has opened up exciting possibilities in regenerative medicine, where emerging drug therapies aim to harness and reprogram cells to repair or regenerate damaged tissues. An emerging class of medical drugs will activate the body’s natural regenerative abilities, offering the potential to restore tissue function lost due to aging, injury, or disease.

## Abbreviations

AAV: adeno-associated virus
ABM: Ascl1, Brn2 and Myt1l
AD: Alzheimer disease
ADRC: adipose-derived regenerative cell
AI: artificial intelligence
ALS: amyotrophic lateral sclerosis
AMD: age-related macular degeneration
ASO: antisense oligonucleotide
ATMP: advanced therapy medicinal product
BBB: blood—brain barrier
BM: basement membrane
BDNF: brain-derived neurotrophic factor
BP-NCD: branched polyethyleneimine coated nitrogen-enriched carbon dots
BrdU: Bromodeoxyuridine
CD-AOF: chemically defined animal origin—free
CiPSC: chemically induced pluripotent stem cell
CNS: central nervous system
2D: 2-dimensional
3D: 3-dimensional
DMD: Duchenne muscular dystrophy
ECM: extracellular matrix
EGR2: early growth response protein 2
EMT: epithelial-to-mesenchymal transition
EPI: epiblast
ESC: embryonic stem cell
EV: extracellular vesicle
FAD: familial Alzheimer disease
FDA: US Food and Drug Administration
FGF: fibroblast growth factor
FNLM: FH-peptide-modified neutrophil-mimicking membrane
GMT: GATA4, MEF2C and TBX5
HCV: hepatitis C virus
HDF: human dermal fibroblast
hESC: human embryonic stem cell
HFF: human foreskin fibroblast
HIP: hypoimmune induced pluripotent stem cell
hiPSC: human induced pluripotent stem cell
hPSC: human pluripotent stem cell
HLA: human leukocyte antigen
HSC: hematopoietic stem cell
hUC: human urine cell
iCM: induced cardiomyocyte
ICM: inner cell mass
iHep: induced hepatocyte
IL: interleukin
ILC: innate lymphoid cell
iN: induced neuron
iPSC: induced pluripotent stem cell
iPSC-CM: induced pluripotent stem cell—derived cardiomyocyte
IRD: inherited retinal disease
LQTS: long-QT syndrome
MEF: mouse embryonic fibroblast
MG: Müller glia
MI: myocardial infarction
ML: machine learning
miR: microRNA
MPS: microphysiological system
MSC: mesenchymal stem cell
MST: MYOCD, SMAD6 and TBX20
MT: MEF2C and TBX5
mtDNA: mitochondrial DNA
MuSC: muscle stem cell
NCC: neural crest cell
NeuroD1: neuronal differentiation 1
NK: natural killer
NMJ: neuromuscular junction
NSC: neural stem cell
OCA: oculocutaneous albinism
OSK: Oct3/4, Sox2, Klf4
OSKM: Oct3/4, Sox2, Klf4, c-Myc
OPC: oligodendrocyte progenitor cell
PBMC: peripheral blood mononuclear cells
PD: Parkinson disease
PE: primitive endoderm
PNS: peripheral nervous system
RepSox: E-616452, ALK5 inhibitor
RGC: retinal ganglion cell
RMAT: Regenerative Medicine Advanced Therapy
RPE: retinal pigment epithelium
SCI: spinal cord injury
SCNT: somatic cell nuclear transfer
SMA: spinal muscular atrophy
SVZ: subventricular zone
T1D: type 1 diabetes
TE: trophectoderm
TGF: transforming growth factor
TNF-*α*: tumor necrosis factor alpha
TNT: tissue nanotransfection
U-iIO: human urine cell—derived induced intestinal organoid
VEGF: vascular endothelial growth factor
VF: vasculogenic fibroblast
VKH: Vogt-Koyanagi-Harada
VPA: valproic acid
WAT: white adipose tissue
